# Structure–Activity Relationship Studies of Tetracyclic Pyrrolocarbazoles Inhibiting Heterotetrameric Protein Kinase CK2

**DOI:** 10.3390/molecules30010063

**Published:** 2024-12-27

**Authors:** Lukas Kröger, Sebastian Borgert, Miriam Lauwers, Michaela Steinkrüger, Joachim Jose, Markus Pietsch, Bernhard Wünsch

**Affiliations:** 1 Institut für Pharmazeutische und Medizinische Chemie, Universität Münster, Corrensstraße 48, D-48149 Münster, Germany; lukas_kroeger@yahoo.de (L.K.); sebi.borgert@gmx.de (S.B.); joachim.jose@uni-muenster.de (J.J.); 2 Institutes I & II of Pharmacology, Center of Pharmacology, Faculty of Medicine and University Hospital Cologne, University of Cologne, Gleueler Straße 24, D-50931 Cologne, Germany; miriam.lauwers@gmail.com (M.L.); michaela.steinkrueger@gmail.com (M.S.); markus.pietsch@th-koeln.de (M.P.); 3 Faculty of Applied Natural Sciences, TH Köln-University of Applied Sciences, Campus Leverkusen, Campusplatz 1, D-51379 Leverkusen, Germany

**Keywords:** CK2, protein kinase, heterotetrameric enzyme, protein–protein interaction, enzyme inhibition, Levy reaction, stereochemistry, structure–activity relationships, microscale thermophoresis, capillary electrophoresis, surface enlargement

## Abstract

The serine/threonine kinase CK2 (formerly known as casein kinase II) plays a crucial role in various CNS disorders and is highly expressed in various types of cancer. Therefore, inhibiting this key kinase could be promising for the treatment of these diseases. The CK2 holoenzyme is formed by the recruitment of two catalytically active CK2α and/or CK2α′ subunits by a regulatory CK2β dimer. Starting with the lead furocarbazole W16 (**4**) inhibiting the CK2α/CK2β interaction, analogous pyrrolocarbazoles were prepared and tested for their protein–protein interaction inhibition (PPII). The key step of the synthesis was a multicomponent Levy reaction of 2-(indolyl)acetate **6**, benzaldehydes **7**, and *N*-substituted maleimides **8**. Targeted modifications were performed by the saponification of the tetracyclic ester **9a**, followed by the coupling of the resulting acid **10** with diverse amines. The replacement of the O-atom of the lead furocarbazole **4** by an N-atom in pyrrolocarbazoles retained or even increased the inhibition of the CK2α/CK2β interaction. The large benzyloxazolidinyl moiety of **4** could be replaced by smaller *N*-substituents without the loss of the PPII. The introduction of larger substituents at the 2-position and/or at *p*-position of the phenyl moiety at the 10-position to increase the surface for the inhibition of the PPI did not enhance the inhibition of the CK2α/CK2β association. The strong inhibition of the CK2α/CK2β association by the histidine derivative (+)-**20a** (*K*_i_ = 6.1 µM) translated into a high inhibition of the kinase activity of the CK2 holoenzyme (CK2α_2_β_2_, IC_50_ = 2.5 µM). Thus, **20a** represents a novel lead compound inhibiting CK2 via the inhibition of the association of the CK2α and Ck2β subunits.

## 1. Introduction

Human serine/threonine kinase CK2 (formerly known as casein kinase II) was identified 70 years ago as the first enzyme able to phosphorylate proteins [1]. The constitutively active CK2 can transfer phosphate moieties from ATP or GTP to more than 500 target proteins [2,3,4]. Due to its broad substrate scope, it is involved in several physiological and pathophysiological processes, including the regulation of the cell cycle, cell movement, cell proliferation, apoptosis, and CNS activity [3,4,5,6,7,8].

The heterotetrameric CK2 holoenzyme is formed by the recruitment of two CK2α and/or CK2α′ subunits to a CK2β dimer [9]. The catalytically active CK2α (CK2α′) subunits contain an ATP binding site each and are responsible for phosphate transfer to the target proteins [9]. The regulatory CK2β subunits stabilize the heterotetrameric holoenzyme and modulate its reactivity and substrate selectivity [9,10,11]. However, the CK2α (CK2α′) subunits alone also show kinase activity. Within the cell, the heterotetrameric holoenzyme and its monomeric CK2α (CK2α′) and CK2β subunits are in a dynamic equilibrium, which contributes to the control of the enzymatic activity in the cell [12].

The kinase CK2 is involved in various central nervous system (CNS) disorders. Recently, it was shown that Okur–Chung neurodevelopmental syndrome (OCNDS) [13] and Poirier–Bienvenu neurodevelopmental syndrome (POBINDS) [14] are related to mutations in the genes for the CK2α and CK2β subunits. Furthermore, increased levels of CK2 have been detected in neurons with pathological neurofibrillary tangles (Alzheimer’s disease) [15,16], and CK2 was identified to contribute to the formation of Levy bodies during the development of Parkinson’s disease [17,18]. In addition to CNS disorders, increased levels of CK2 inducing cell proliferation and inhibiting apoptosis were observed in several types of cancer [19,20,21,22]. Although widely expressed, CK2 represents a promising target for the development of novel therapies to combat cancer, since non-cancer cells appear to be less sensitive to CK2 inhibition with respect to cell proliferation and apoptosis [23,24].

**Figure 1:**
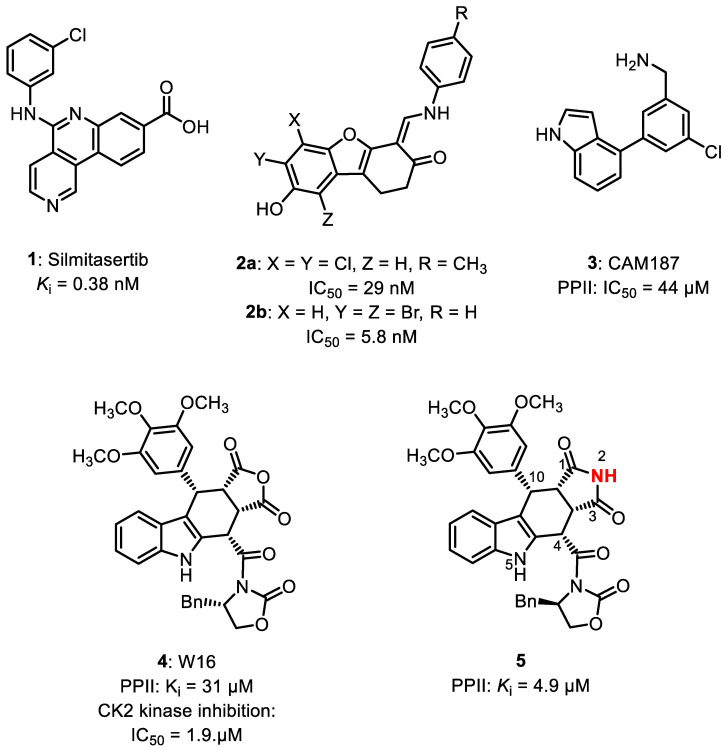
Silmitasertib (**1**, CX-4945) and dibenzofurans **2** inhibit CK2 in an ATP competitive manner. CAM187 (**3**) interacts selectively with the CK2α subunit, inhibiting its association with the CK2β subunit but barely interfering with substrate phosphorylation. In contrast, the tetracyclic compounds **4** (W16) and **5** prevent the CK2α/CK2β association and modulate the kinase activity.

Several CK2 inhibitors differing in efficiency and selectivity have been developed as potential new antitumor drugs [25,26,27]. The tricyclic carboxylic acid silmitasertib (**1**, CX-4945, Figure 1) targeting the ATP binding site of the CK2α (CK2α′) subunit [28,29] has been clinically evaluated for the treatment of cholangiocarcinoma (bile duct cancer) [30]. Dibenzofurans **2** were shown to strongly inhibit CK2 in an ATP competitive manner. Whereas the dichloro derivative **2a** revealed an IC_50_ value of 29 nM [31], the regioisomeric dibromo derivative **2b** was 5-fold more potent (IC_50_ = 5.8 nM), highly selective for CK2, and showed antiproliferative activity against a human prostate tumor cell line [32]. However, ligands interacting with the ATP binding site usually suffer from lower selectivity, since all other protein kinases contain a similarly structured ATP binding site as well [33,34].

In another approach, the association of CK2α and CK2β subunits is suppressed by protein–protein interaction inhibitors (PPIIs). Thus, peptide-based PPIIs such as the cyclic tridecapeptide Pc (13 amino acids) and its derivatives prevent the formation of the hetero-tetrameric CK2 holoenzyme by the inhibition of the CK2α/CK2β association [35,36,37]. In contrast to peptides, the 4-phenylindole CAM187 (**3**, Figure 1) is hydrolytically stable and blocks the CK2α/CK2β interaction (IC_50_ = 44 µM) by binding at the CK2α subunit, but not ATP competitively [38]. Although **3** could inhibit the CK2α/CK2β association, the kinase activity of CK2 was not significantly affected [38].

In addition to peptides and CAM187 (**3**), W16 (**4**, Figure 1) with a tetracyclic furocarbazole scaffold was reported to inhibit the CK2α/CK2β interaction, with an IC_50_ value of 30–40 µM, and the kinase activity of the CK2α subunit, with an IC_50_ value of 20 µM [39]. We recently confirmed these inhibitory activities of W16 by the determination of a *K*_i_ value of 31 µM and an IC_50_ value of 1.9 µM, respectively (see below). In this context, we also reported on the replacement of the furan ring of **4** by a pyrrole ring, leading to an increased inhibition of the CK2α/CK2β association. As an example, the (3a*S*,4*S*,10*S*,10a*S*)-configured pyrrolocarbazole **5** exhibited a *K*_i_ value of 4.9 µM [40]. Since the lead compounds **4** and **5** inhibit protein–protein interactions, we planned to extend their surface to increase their interactions with the rather flat protein surface. In particular, substituents at the imide N-atom in the 2-position, at the carboxamide in the 4-position, and at the phenyl moiety in the 10-position should be modified. The designed extended pyrrolocarbazoles do not fulfill the rule of five defined by Lipinski et al. [41], as their molecular weight exceeds the upper limit of 500 Da. However, in contrast to drugs addressing the structured binding pockets of enzymes or receptors, protein–protein interaction inhibitors have to interact with a large and rather flat surface of one protein. Therefore, we planned to extend the pyrrolocarbazole system in three directions to enlarge the contacts with the protein surface.

## 2. Results and Discussion

### 2.1. Synthesis of Tetracyclic Pyrrolocarbazoles

**Scheme 1:**
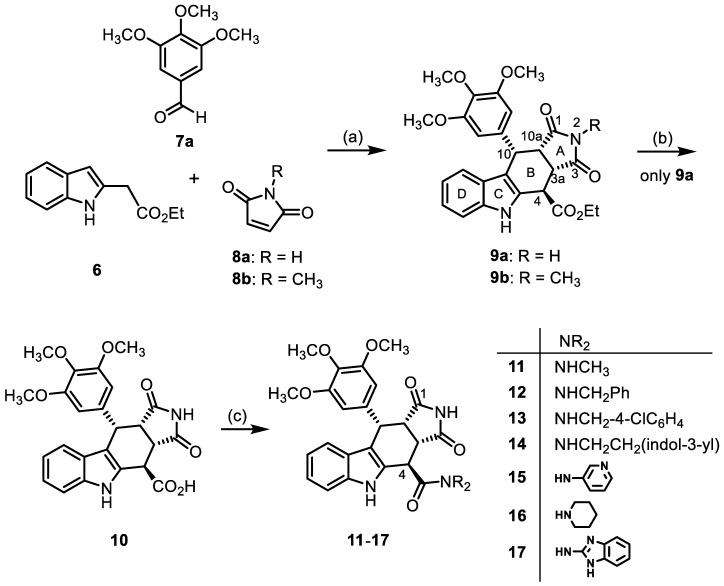
Synthesis of racemic *cis*,*cis*,*trans*-configured pyrrolocarbazole derivatives with various 4-carboxamides. Reagents and reaction conditions: (a) CuSO_4_.5H_2_O, *o*-xylene, reflux (pressure-resistant Schlenk tube), 16 h, 79% (**9a**), 83% (**9b**). (b) **9a**: NaOH, H_2_O, THF, rt, 30 min, 96%. (c) R_2_NH, COMU^®^, DIPEA, THF or EtOAc, 0–20 °C, 1–4 h, 49% (**11**), 84% (**12**), 71% (**13**), 52% (**14**), 12% (**15**), 66% (**16**), 23% (**17**). The formulas **9**–**17** show only the relative configuration of one enantiomer of the racemic mixtures.

The tetracyclic framework was prepared by a three-component Levy reaction [42,43]. In a domino reaction, 2-(indolyl)acetate **6** reacted first with trimethoxybenzaldehyde (**7a**) to form a diene, which underwent a Diels–Alder reaction with maleimides **8**, diastereoselectively providing the *cis*,*cis*,*trans*-configured pyrrolocarbazoles **9a** and **9b** in 79% and 83% yields, respectively. The Diels–Alder reaction with maleimides led to the kinetically favored *cis*,*cis*,*cis*-configured diastereomers, which epimerized at C-4 in the presence of CuSO_4_ in refluxing *o*-xylene to give the thermodynamically favored *cis*,*cis*,*trans*-configured diastereomers **9a** and **9b** [40,43] (Scheme 1).

In order to obtain diverse ligands, the ester **9a** was hydrolyzed with NaOH and the resulting acid **10** [40] was coupled with various primary and secondary amines in the presence of the coupling agent COMU^®^ [44]. Thus, a diverse set of secondary and tertiary amides **11**–**17** was obtained. The low yields of **15** and **17** were due to purification issues (Scheme 1).

**Scheme 2:**
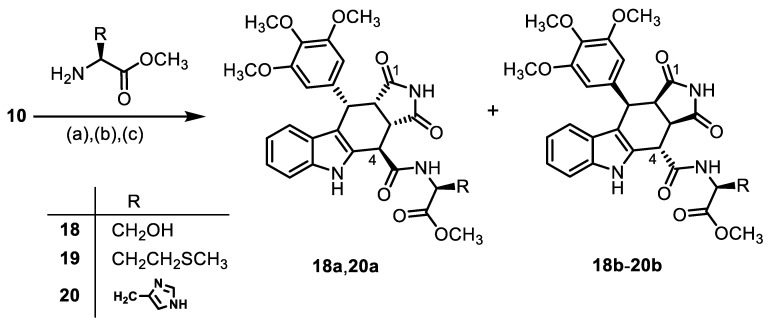
Synthesis of enantiomerically pure *cis*,*cis*,*trans*-configured pyrrolocarbazole derivatives **18**–**20** coupled with amino acids. Reagents and reaction conditions: (a) COMU^®^, DIPEA, DMF, 0 °C, 2 h, 27% (**18a**), 27% (**18b**). (b) CDI, CH_2_Cl_2_, rt, 48 h, 22% (**19b**). (c) COMU^®^, DIPEA, THF, rt, 16 h, 20% (**20a**.HCl), 29% (**20b**). The formulas **18**–**20** show the absolute configuration of enantiomerically pure pyrrolocarbazoles.

COMU^®^ coupling of the acid **10** with enantiomerically pure primary amines derived from amino acids led to diastereomeric secondary amides **18a**,**b**–**20a**,**b**. After the separation of diastereomers, the enantiomerically pure secondary amides **18a**,**b**, **19b**, and **20a**,**b** were isolated (Scheme 2).

**Scheme 3:**
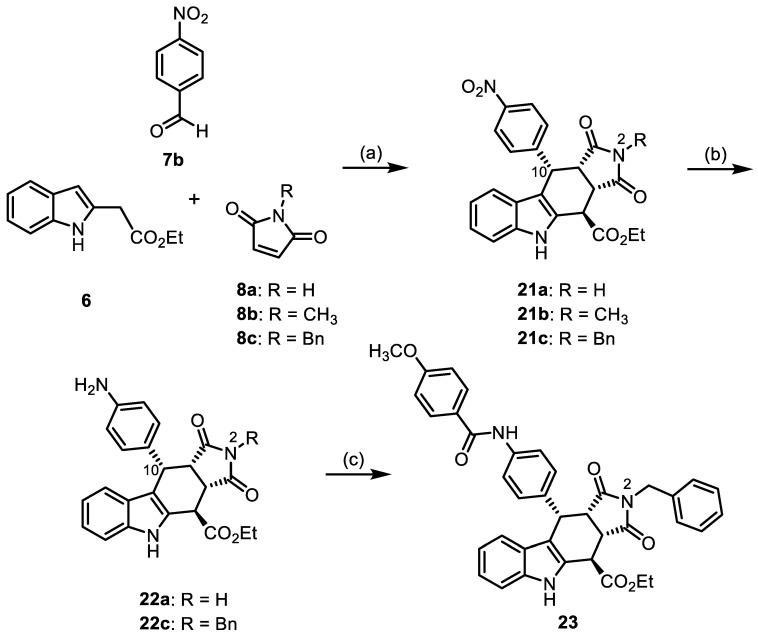
Synthesis of racemic *cis*,*cis*,*trans*-configured pyrrolocarbazole derivatives with extended π-systems. Reagents and reaction conditions: (a) CuSO_4_.5H_2_O, *o*-xylene, 180 °C (pressure-resistant Schlenk tube), 24–40 h, 25% (**21a**), 40% (**21b**), 40% (**21c**). (b) Zn, THF, NH_4_Cl, rt, 2 h, 62% (**22a**.HCl), 48% (**22c**.HCl). (c) 4-Methoxybenzoyl chloride, DIPEA, CH_2_Cl_2_, rt, 2 h, 82%. The formulas **21–23** show only the relative configuration of one enantiomer of the racemic mixtures.

In order to increase potential protein–protein interactions, the tetracyclic pyrrolocarbazole framework was expanded at the 2-position (imide N-atom) and at the phenyl moiety in the 10-position. For this purpose, the Levy reaction was performed with 2-(indolyl)acetate **6**, 4-nitrobenzaldehyde (**7b**) and maleimides **8a**-**c**, bearing various substituents at the N-atom, to obtain the *cis*,*cis*,*trans*-configured pyrrolocarbazoles **21a**–**c** in 25–40% yields. After the reduction of the NO_2_ moiety with Zn/NH_4_Cl, the primary amine **22c** was acylated with 4-methoxybenzoyl chloride to afford the amide **23** in an 82% yield. In the benzamide **23**, the tetracyclic pyrrolocarbazole framework was extended at the 2- and 10-positions (Scheme 3). 

### 2.2. Pharmacological Evaluation

#### 2.2.1. Inhibition of the CK2α/CK2β Interaction

A microscale thermophoresis (MST) assay was used to determine the interaction of the CK2α and CK2β subunits. In this assay, a constant concentration of the fluorescently labeled CK2β subunit was added to increasing concentrations of the CK2α subunit and the thermophoretic shift was recorded, respectively. These experiments resulted in a dissociation constant (*K*_D_ value) of 11 nM for the CK2α/CK2β interaction, which is in agreement with reported values [40,45]. The same experiment was performed in the presence of 20, 50, or 100 µM of the test compounds (see Table 1). An increased *K*_D_′ value in the presence of the test compounds was evaluated as the inhibition of the association of the CK2α and CK2β subunits. The increased *K*_D_′ value was then transformed together with the employed concentration of the test compound into the *K*_i_ value for the inhibition of the CK2α/CK2β association (formula see Supporting Information). The recorded *K*_D_ and *K*_D_′ values, as well as the calculated *K*_i_ values for the tetracyclic pyrrolocarbazoles **9**–**23** and the lead compound W16 (**4**), are displayed in Table 1.

Table 1 shows that the PPII of several prepared pyrrolocarbazoles exceeded the activity of the lead compound **4**, and some compounds even revealed *K*_i_ values below 10 µM. Particularly high activity with *K*_i_ values below 7 µM was observed for the racemic acid (±)-**10** (*K*_i_ = 1.9 µM), *N*-methylcarboxamide (±)-**11** (*K*_i_ = 6.0 µM), the 2-(indolyl)ethyl derivative (±)-**14** (*K*_i_ = 3.8 µM), and the enantiomerically pure histidine derivative (+)-**20a** (*K*_i_ = 6.1 µM). These results indicate that the large benzyloxazolidinyl substituent of the lead compound **4** is not essential to achieve a high inhibition of the CK2α/CK2β association. Smaller substituents at the carboxamide in the 4-position, such as the small methyl moiety of **11**, also lead to a high PPII. However, an ester moiety instead of the carboxamide in the 4-position (e.g., **9**, **21**–**23**) is not appropriate to strongly inhibit the CK2α/CK2β association. An extension at the 2-position with an additional benzyl moiety, as in **22c**, or an extension of the phenyl moiety at the 10-postion, as in **23**, did not result in a significant inhibition of the CK2α/CK2β association.

#### 2.2.2. Inhibition of the Enzymatic Activity

The inhibition of the catalytic activity of the CK2 holoenzyme (CK2α_2_β_2_) was determined in an enzyme assay. In brief, the CK2 holoenzyme was incubated with the decapeptide RRRDDDSDDD and ATP. The amount of phosphorylated peptide (Ser7) was recorded by capillary electrophoresis (CE) [40,46]. In this assay, the lead compound W16 (**4**) inhibited the CK2 activity with an IC_50_ value of 1.9 µM [40].

In a first screening with a test compound concentration of 10 µM, only the histidine derivative (+)-**20a** exhibited more than 50% enzyme inhibition, indicating an IC_50_ value below 10 µM. Therefore, concentration-dependent CK2 inhibition was recorded only for (+)-**20a**, resulting in an IC_50_ value of 2.5 µM, which is close to the IC_50_ value of **4**. Thus, the strong inhibition of the CK2α/CK2β association (*K*_i_ = 6.1 µM) by the histidine derivative (+)-**20a** was transferred into a strong inhibition of the enzymatic activity of the CK2 holoenzyme (IC_50_ = 2.5 µM).

## 3. Conclusions

In order to study the relationships between the structural features and the inhibition of the interactions between the CK2α and CK2β subunits of the protein kinase CK2, a series of pyrrolocarbazoles was prepared. The thermodynamically favored *cis*,*cis*,*trans*-configured pyrrolocarbazoles were obtained by a multicomponent Levy reaction as the key step allowing for modifications at the 2-, 4-, and 10-positions of the tetracyclic framework. The replacement of the O-atom of the furocarbazole lead compound W16 (**4**) by an N-atom retained or even increased the inhibition of the CK2α/CK2β association. Smaller N-substituents of the carboxamide in the 4-position instead of the large benzyloxazolidinyl substituent of the lead compound **4** were well tolerated and increased the PPII, as shown for the *N*-methylcarboxamide **11** (*K*_i_ = 6.0 µM), the *N*-indolylethylcarboxamide **14** (*K*_i_ = 3.8 µM), and the histidine derivative (+)-**20a** (*K*_i_ = 6.1 µM). Additional substituents at the imide N-atom in the 2-position or in the *p-*position at the phenyl moiety in the 10-position to increase the interactions with the protein surface did not lead to an increased inhibition of the CK2α/CK2β association. The inhibition of the CK2α/CK2β association of the histidine derivative (+)-**20a** translated into an inhibition of the kinase activity of the CK2 holoenzyme (IC_50_ = 2.5 µM), which is in the same range as the CK2 inhibition of the furocarbazole lead compound **4**.

## 4. Experimental Part

### 4.1. Chemistry, General Methods

Oxygen- and moisture-sensitive reactions were carried out under nitrogen dried with silica gel with a moisture indicator (orange gel, VWR, Darmstadt, Germany) and in dry glassware (Schlenk flask or Schlenk tube). The temperature was controlled with dry ice/acetone (−78 °C), ice/water (0 °C), a Cryostat (Julabo TC100E-F, Seelbach, Germany), a magnetic stirrer MR 3001 K (Heidolph, Schwalbach, Germany), or am RCT CL (IKA, Staufen, Germany), together with the temperature controller EKT HeiCon (Heidolph) or VT-5 (VWR) and PEG or a silicone bath. All solvents were of analytical- or technical-grade quality. *o*-Xylene and toluene were dried with molecular sieves (3 Å). Demineralized water was used. Thin-layer chromatography (tlc) was used with tlc silica gel 60 F_254_ on aluminum sheets (VWR). Flash chromatography (fc) was used with silica gel 60, 40–63 µm (VWR) with parentheses including the diameter of the column (Ø), length of the stationary phase (l), fraction size (v), and eluent. Automated flash chromatography used Isolera^TM^ Spektra One (Biotage^®^, Global Go-to Separations Company, Stockholm, Sweeden), with parentheses including the cartridge size, flow rate, eluent, and a fractions size always of 20 mL. The melting point system MP50 (Mettler Toledo, Gießen, Germany) was open-capillary and uncorrected. A MicroTOFQII mass spectrometer (Bruker Daltonics, Bremen, Germany) was used, and deviations of the exact masses found from the calculated exact masses were 5 mDa or less; the data were analyzed with Data-Analysis^®^ (Bruker Daltonics). NMR spectra were recorded in deuterated solvents on Agilent DD2 400 MHz and 600 MHz spectrometers (Agilent, Santa Clara, CA, USA); chemical shifts (*δ*) are reported in parts per million (ppm) against the reference substance tetramethylsilane and were calculated using the solvent residual peak of the undeuterated solvent. Coupling constants are given with a 0.5 Hz resolution. The assignment of ^1^H and ^13^C NMR signals was supported by 2-D NMR techniques where necessary. Am FT/IR Affinity^®^-1 spectrometer (Shimadzu, Düsseldorf, Germany) using ATR technique was utilized.

### 4.2. HPLC Method for the Determination of the Purity

Equipment 1, as follows: Pump: L-7100, degasser: L-7614, autosampler: L-7200, UV detector: L-7400, interface: D-7000, data transfer: D-line, and data acquisition: HSM-Software (all from Merck Hitachi, Darmstadt, Germany). Equipment 2, as follows: Pump: LPG-3400SD, degasser: DG-1210, autosampler: ACC-3000T, UV-detector: VWD-3400RS, interface: DIONEX UltiMate 3000, and data acquisition: Chromeleon 7 (equipment and software from Thermo Fisher Scientific, Lauenstadt, Germany). The column was as follows: LiChrospher^®^ 60 RP-select B (5 µm), LiChroCART^®^ 250–4 mm cartridge. The flow rate was 1.0 mL/min, the injection volume was 5.0 µL, and detection was performed at λ = 210 nm. The solvents were as follows: A: demineralized water with 0.05% (*V*/*V*) trifluoroacetic acid, B: CH_3_CN with 0.05% (*V*/*V*) trifluoroacetic acid; gradient elution (% A): 0–4 min: 90%; 4–29 min: gradient from 90% to 0%; 29–31 min: 0%; 31–31.5 min: gradient from 0% to 90%; 31.5–40 min: 90%.

### 4.3. Synthetic Procedures

**Ethyl (3a*RS*,4*SR*,10*RS*,10a*RS*)-1,3-dioxo-10-(3,4,5-trimethoxyphenyl9a)-1,2,3,3a,4,5,10,10a-octahydropyrrolo[3,4-*b*]carbazole-4-carboxylate () [40]**

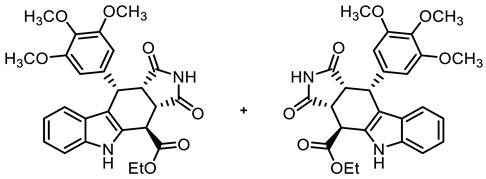

Under N_2_, indolylacetate **6** (203 mg, 1.00 mmol), maleimide (**8a**, 292 mg, 3.01 mmol) and 3,4,5-trimethoxybenzaldehyde (**7a**, 296 mg, 1.51 mmol) were dissolved in dry *o*-xylene (10 mL) in a pressure-resistant Schlenk tube. Crushed CuSO_4_ ∙ 5 H_2_O (25.2 mg, 0.10 mmol) was added to the solution and the mixture was heated to reflux for 16 h (oil bath temperature of 185 °C). After cooling to room temperature, the mixture was filtered and the filter was washed with CH_2_Cl_2_ (3 × 10 mL). The filtrate was concentrated in vacuo and the residue was purified by flash column chromatography (ethyl acetate/cyclohexane = 45:55, Ø 4 cm, h = 16 cm, v = 30 mL). Yellow solid, mp 220 °C, yield of 379 mg (79%). C_26_H_26_N_2_O_7_ (478.5). TLC (ethyl acetate/CH_2_Cl_2_ = 3:7): R_f_ = 0.38.
Purity (HPLC): 97.4%, (t_R_ = 19.3 min). ^1^H NMR (600 MHz, DMSO-*d_6_*): δ (ppm) = 1.31 (t, *J* = 7.1 Hz, 3H, OCH_2_C*H_3_*), 3.53–3.57 (m, 1H, 10a-H), 3.57 (s, 3H, 4-OCH_3_), 3.61 (s, 6H, 3-OCH_3_, 5-OCH_3_), 4.05 (dd, *J* = 9.0/3.4 Hz, 1H, 3a-H), 4.20–4.33 (m, 2H, OC*H_2_*CH_3_), 4.49 (d, *J* = 3.5 Hz, 1H, 4-H), 4.74 (d, *J* = 8.3 Hz, 1H, 10-H), 6.21 (s, 2H, 2-H_TMP_, 6-H_TMP_), 6.88 (ddd, *J* = 8.0/7.0/1.0 Hz, 1H, 8-H), 7.05 (ddd, *J* = 8.1/6.9/1.2 Hz, 1H, 7-H), 7.20 (dd, *J* = 8.0/1.1 Hz, 1H, 9-H), 7.37 (dt, *J* = 8.2/0.9 Hz, 1H, 6-H), 10.92 (s, 2H, 2-H, 5-H). ^13^C NMR (151 MHz, DMSO-*d_6_*): δ (ppm) = 14.0 (1C, OCH_2_*C*H_3_), 36.9 (1C, C-4), 38.0 (1C, C-10), 42.5 (1C, C-3a), 45.5 (1C, C-10a), 55.6 (2C, 3-OCH_3_ 5-OCH_3_), 59.9 (1C, 4-OCH_3_), 61.7 (1C, O*C*H_2_CH_3_), 106.0 (2C, C-2_TMP_, 6-C_TMP_), 111.3 (1C, C-9b), 111.5 (1C, C-6), 118.4 (1C, C-9), 118.7 (1C, C-8), 121.6 (1C, C-7), 125.4 (1C, C-9a), 128.5 (1C, C-4a), 136.2 (1C, C-4_TMP_), 136.4 (1C, C-1_TMP_), 136.6 (1C, C-5a), 152.1 (2C, C-3_TMP,_ C-5_TMP_), 171.1 (1C, C-4-*C*=O), 177.7 (1C, C-1), 179.5 (1C, C-3). Exact mass (APCI): *m/z* = 479.1785 (calcd. 479.1813 for C_26_H_27_N_2_O_7_ [MH]^+^). IR (neat): ṽ [cm^−1^] = 2987 (w, C-H_aliph_.), 1775 (w, C=O), 1709 (s, C=O), 1119 (s, C-O), 752 (m, C-H_arom._).

**Ethyl (3a*RS*,4*SR*,10*RS*,10a*RS*)-2-methyl-1,3-dioxo-10-(3,4,5-trimethoxyphenyl)-1,2,3,3a,4,5,10,10a-octahydropyrrolo[3,4-*b*]carbazole-4-carboxylate (9b)**

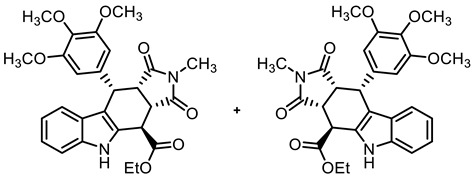

Under N_2_, indolylacetate **6** (3.01 g, 14.8 mmol), *N*-methylmaleimide (**8b**, 5.00 g, 45.0 mmol), and 3,4,5-trimethoxybenzaldehyde (**7a**, 4.42 g, 22.5 mmol) were dissolved in dry *o*-xylene (100 mL). Crushed CuSO_4_ ∙ 5 H_2_O (351 mg, 1.41 mmol) was added to the solution and the mixture was heated to reflux for 16 h. After cooling to -20 °C, the mixture was filtered and the obtained solid was washed with water (3 × 25 mL) and *n*-pentane (5 × 25 mL) to give the product without further purification. Colorless solid, mp 215 °C, yield of 6.32 g (83%). C_27_H_28_N_2_O_7_ (492.5). TLC (ethyl acetate/cyclohexane = 1:1): R_f_ = 0.41. Purity (HPLC): 97.3%, (t_R_ = 20.5 min). ^1^H NMR (600 MHz, DMSO-*d_6_*): δ (ppm) = 1.30 (t, *J* = 7.1 Hz, 3H; OCH_2_C*H_3_*), 2.35 (s, 3H, NC*H_3_*), 3.55 (s, 3H, 4-OCH_3_), 3.61 (s, 6H, 3-OCH_3_, 5-OCH_3_), 3.63 (t, *J* = 8.2 Hz, 1H, 10a-H), 4.06 (dd, *J* = 8.7/3.3 Hz, 1H, 3a-H), 4.21–4.33 (m, 2H, OC*H_2_*CH_3_), 4.57 (d, *J* = 3.0 Hz, 1H, 4-H), 4.76 (d, *J* = 8.0 Hz, 1H, 10-H), 6.13 (s, 2H, 2-H_TMP_, 6-H_TMP_), 6.88 (ddd, *J* = 7.9/7.0/1.0 Hz, 1H, 8-H), 7.05 (ddd, *J* = 8.2/7.0/1.2 Hz, 1H, 7-H), 7.19 (d, *J* = 7.9 Hz, 1H, 9-H), 7.38 (dt, *J* = 8.0/0.9 Hz, 1H, 6-H), 10.96 (s, 1H, 5-H). ^13^C NMR (151 MHz, DMSO-*d_6_*): δ (ppm) = 14.0 (1C, OCH_2_*C*H_3_), 23.7 (1C, NC*H_3_*), 36.6 (1C, C-4), 38.0 (1C, C-10), 41.4 (1C, C-3a), 44.7 (1C, C-10a), 55.7 (2C, 3-OCH_3_, 5-OCH_3_), 59.9 (1C, 4-OCH_3_), 61.8 (1C, OCH_2_*C*H_3_), 106.0 (2C, C-2_TMP_, C-6_TMP_), 110.8 (1C, C-9b), 111.5 (1C, C-6), 118.4 (1C, C-9), 118.7 (1C, C-7), 121.7 (1C, C-8), 125.4 (1C, C-9a), 128.4 (1C, C-4a), 136.1 (1C, C-1_TMP_), 136.3 (1C, C-4_TMP_), 136.7 (1C, C-5a), 152.1 (2C, C-3_TMP_, C-5_TMP_), 171.0 (1C, C-4-*C*=O), 176.4 (1C, C-1), 178.0 (1C, C-3). Exact mass (ESI): *m/z* = 493.1958 (calcd. 493.1969 for C_27_H_29_N_2_O_7_ [MH]^+^). IR (neat): ṽ [cm^−1^] = 2970 (w, C-H_aliph_.), 1743,1701 (s, C=O), 1180, 1115 (s, C-O).

**(3a*RS*,4*SR*,10*RS*,10a*RS*)-1,3-Dioxo-10-(3,4,5-trimethoxyphenyl)-1,2,3,3a,4,5,10,10a-octahydropyrrolo[3,4-*b*]carbazole-4-carboxylic acid (10) [40]**

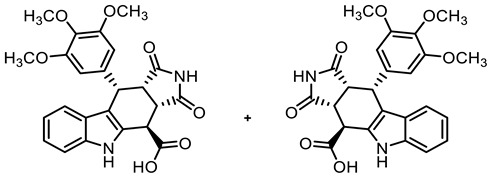

A solution of NaOH (2.60 g, 6.50 mmol) in water (50 mL) was added to a solution of ester **9a** (5.06g, 10.56 mmol) in THF (50 mL). The mixture was stirred for 30 min at room temperature. Ethyl acetate (100 mL) was added and the phases were separated. The aqueous layer was washed with ethyl acetate (3 × 50 mL) to remove non-acidic impurities. HCl solution (2 M in water, 30 mL) was added to the aqueous layer and the resulting suspension was extracted with ethyl acetate (3 × 50 mL). The combined organic layers of the last extraction step were dried (Na_2_SO_4_), filtered, and concentrated in vacuo to obtain the product without further purification. Yellow solid, mp 162 °C, yield of 4.58 g (96%). C_24_H_22_N_2_O_7_ (450.4). TLC (MeOH/CH_2_Cl_2_ = 15:85 + 0.1% acetic acid): R_f_ = 0.33. Purity (HPLC): 94.7%, (t_R_ = 16.5 min). ^1^H NMR (600 MHz, DMSO-*d_6_*): δ (ppm) = 3.54 (t, *J* = 8.4 Hz, 1H, 10a-H), 3.56 (s, 3H, 4-OCH_3_), 3.62 (s, 6H, 3-OCH_3_, 5-OCH_3_), 4.09 (dd, *J* = 9.0/3.6 Hz, 1H, 3a-H), 4.41 (d, *J* = 3.6 Hz, 1H, 4-H), 4.72 (d, *J* = 8.2 Hz, 1H, 10-H), 6.22 (s, 2H, 2-H_TMP_, 6-H_TMP_), 6.87 (ddd, *J* = 7.8/6.8/0.9 Hz, 1H, 8-H), 7.02 (ddd, *J* = 8.2/7.0/1.2 Hz, 1H, 7-H), 7.21 (d, *J* = 7.9 Hz, 1H, 9-H), 7.39 (d, *J* = 8.1 Hz, 1H, 6-H), 10.89 (s, 1H, 2-H), 10.92 (s, 1H, 5-H), 13.40 (s, 1H, COO*H*). ^13^C NMR (151 MHz, DMSO-*d_6_*): δ (ppm) = 37.0 (1C, C-4), 38.1 (1C, C-10), 42.3 (1C, C-3a), 45.5 (1C, C-10a), 55.6 (2C, 3-OCH_3_, 5-OCH_3_), 59.9 (1C, 4-OCH_3_), 106.0 (2C, C-2_TMP_, C-6_TMP_), 111.1 (1C, C-9b), 111.6 (1C, C-6), 118.2 (1C, C-9), 118.6 (1C, C-8), 121.4 (1C, C-7), 125.4 (1C, C-9a), 128.9 (1C, C-4a), 136.1 (1C, C-4_TMP_), 136.5 (1C, C-1_TMP_), 136.6 (1C, C-5a), 152.1 (2C, C-3_TMP_, C-5_TMP_), 172.4 (1C, *C*OOH), 177.8 (1C, C-1), 179.9 (1C, C-3). Exact mass (ESI): *m/z* = 451.1477 (calcd. 451.1500 for C_24_H_23_N_2_O_7_ [MH]^+^). IR (neat): ṽ [cm^−1^] = 2939, 2835 (m, C-H_aliph_.), 1709 (s, C=O), 1119 (m, C-O), 745 (m, C-H_arom._).

**(3a*RS*,4*SR*,10*RS*,10a*RS*)-*N*-Methyl-1,3-dioxo-10-(3,4,5-trimethoxyphenyl)-1,2,3,3a,4,5,10,10a-octahydropyrrolo[3,4-*b*]carbazole-4-carboxamide (11)**

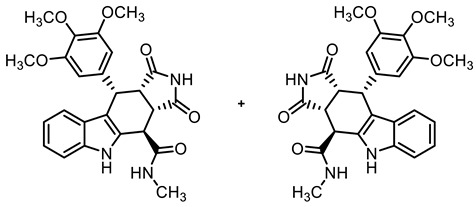

Carboxylic acid **10** (212 mg, 0.47 mmol), COMU^®^ (242 mg, 0.56 mmol), and DIPEA (140 mg, 1.08 mmol) were dissolved in dry THF (15 mL) at 0 °C. Then, a solution of methylamine (17.6 mg, 0.56 mmol) in THF (2 mL) was added dropwise. The mixture was stirred for 2 h at 0 °C. After the completion of the transformation, the solution was allowed to warm to ambient temperature and ethyl acetate (30 mL) was added. The mixture was extracted with HCl solution (1 M in water, 2 × 30 mL) and saturated NaCl solution. The organic layer was dried (Na_2_SO_4_), filtered, and concentrated in vacuo. The residue was purified by automatic flash column chromatography (cartridge: SNAP 50 g, flow rate of 50 mL/min, ethyl acetate) to yield a yellow solid. For further purification, the obtained solid was dissolved in ethyl acetate (50 mL) and extracted with NaHCO_3_ solution (0.1 M in water, 3 × 50 mL). The organic layer was dried (Na_2_SO_4_), filtered, and the solvent was removed in vacuo. Yellow solid, mp 215 °C, yield of 106 mg (49%). C_25_H_25_N_3_O_6_ (463.5). TLC (ethyl acetate): R_f_ = 0.31. Purity (HPLC): 95.6%, (t_R_ = 16.0 min). ^1^H NMR (600 MHz, DMSO-*d_6_*): δ (ppm) = 2.81 (d, *J* = 4.6 Hz, 3H, NHC*H_3_*), 3.52 (dd, *J* = 9.6/7.9 Hz, 1H, 10a-H), 3.55 (s, 3H, 4-OCH_3_), 3.65 (s, 6H, 3-OCH_3_, 5-OCH_3_), 4.00 (dd, *J* = 9.5/5.0 Hz, 1H, 3a-H), 4.36 (d, *J* = 5.0 Hz, 1H, 4-H), 4.74 (d, *J* = 7.9 Hz, 1H, 10-H), 6.27 (s, 2H, 2-H_TMP_, 6-H_TMP_), 6.90 (ddd, *J* = 8.0/7.0/1.0 Hz, 1H, 8-H), 7.03 (ddd, *J* = 8.1/7.0/1.1 Hz, 1H, 7-H) 7.34–7.36 (m, 1H, 9-H), 7.36–7.37 (m, 1H, 6-H), 8.61 (q, *J* = 4.6 Hz, 1H, NH_amide_), 10.67 (s, 1H, 2-H), 10.88 (s, 1H, 5-H). ^13^C NMR (151 MHz, DMSO-*d_6_*): δ (ppm) = 26.3 (1C, NH*C*H_3_), 38.1 (1C, C-10), 38.6 (1C, C-4), 42.6 (1C, C-3a), 46.2 (1C, C-10a), 55.7 (2C, 3-OCH_3_, 5-OCH_3_), 59.9 (1C, 4-OCH_3_), 105.7 (2C, C-2_TMP_, C-6_TMP_), 111.4 (1C, C-9b), 111.6 (1C, C-6), 117.8 (1C, C-9), 118.6 1C, C-8), 121.2 (1C, C-7), 125.5 (1C, C-9a), 131.3 (1C, C-4a), 136.1 (1C, C-4_TMP_), 136.3 (1C, C-1_TMP_), 137.0 (1C, C-5a), 152.2 (1C, C-3_TMP_, C-5_TMP_), 170.5 (1C, *C*O_amide_), 178.1 (1C, C-1), 180.4 (1C, C-3). Exact mass (ESI): *m/z* = 464.1818 (calcd. 451.1816 for C_25_H_25_N_3_O_6_ [MH]^+^). IR (neat): ṽ [cm^−1^] = 2978, 2889 (m, C-H_aliph_.), 1697, 1678 (s, C=O), 1119 (m, C-O), 741 (m, C-H_arom._).

**(3a*RS*,4*SR*,10*RS*,10a*RS*)-*N*-Benzyl-1,3-dioxo-10-(3,4,5-trimethoxyphenyl)-1,2,3,3a,4,5,10,10a-octahydropyrrolo[3,4-*b*]carbazole-4-carboxamide (12)**

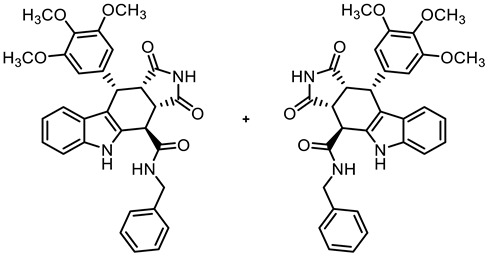

Carboxylic acid **10** (135 mg, 0.30 mmol), COMU^®^ (168 mg, 0.39 mmol), and DIPEA (90.6 mg, 0.70 mmol) were dissolved in dry THF (5 mL) at room temperature. Then, a solution of benzylamine (42.1 mg, 0.39 mmol) in THF (2 mL) was added dropwise. The mixture was stirred for 2 h at room temperature. After the completion of the transformation, ethyl acetate (50 mL) was added. The mixture was extracted successively with NaHCO_3_ solution (0.1 M in water, 3 × 25 mL), HCl solution (1 M in water, 3 × 25 mL), and saturated NaCl solution (3 × 25 mL). The organic layer was dried (Na_2_SO_4_), filtered, and concentrated in vacuo. The residue was purified by flash column chromatography (ethyl acetate/cyclohexane = 6:4, Ø 2 cm, h = 18 cm, v = 10 mL). Yellow solid, mp 157 °C, yield of 136 mg (84%). C_31_H_29_N_3_O_6_ (539.6). TLC (ethyl acetate/cyclohexane = 6:4): R_f_ = 0.41. Purity (HPLC): 96.9%, (t_R_ = 19.3 min). ^1^H NMR (600 MHz, DMSO-*d_6_*): δ (ppm) = 3.56 (s, 3H, 4-OCH_3_), 3.57 (dd, *J* = 9.5/8.0 Hz, 1H, 10a-H), 3.64 (s, 6H, 3-OCH_3_, 5-OCH_3_), 4.03 (dd, *J* = 9.0/4.9 Hz, 1H, 3a-H), 4.26 (dd, *J* = 15.2/4.2 Hz, 1H, PhC*H_2_*NH), 4.48 (d, *J* = 5.0 Hz, 1H, 4-H), 4.75 (dd, *J* = 15.3/7.3 Hz, 1H, PhC*H_2_*NH), 4.76 (d, *J* = 8.1 Hz, 1H, 10-H), 6.29 (s, 2H, 2-H_TMP_, 6-H_TMP_), 6.91 (ddd, *J* = 7.9/7.0/1.0 Hz, 1H, 8-H), 7.04 (ddd, *J* = 8.2/7.0/1.2 Hz, 1H, 7-H), 7.28 (tt, *J* = 7.3/1.7 Hz, 1H, 4-H_benzyl_), 7.33–7.39 (m, 4H, 6-H, 9-H, 3-H_benzyl_, 5-H_benzyl_), 7.40–7.42 (m, 2H, 2-H_benzyl_, 6-H_benzyl_), 9.09 (dd, *J* = 7.3/4.3 Hz, 1H, NH_amide_), 10.73 (s, 1H, 2-H), 10.91 (s, 1H, 5-H). ^13^C NMR (151 MHz, DMSO-*d_6_*): δ (ppm) = 38.1 (1C, C-10), 38.7 (1C, C-4), 42.9 (1C, C-3a), 43.1 (1C, Ph*C*H_2_NH), 46.1 (1C, C-10a), 55.7 (2C, 3-OCH_3,_ 5-OCH_3_), 59.9 (1C, 4-OCH_3_), 105.8 (2C, C-2_TMP_, C-6_TMP_), 111.3 (1C, C-9b) 111.7 (1C, C-6), 117.9 (1C, C-9), 118.7 (1C, C-8), 121.3 (1C, C-7), 125.5 (1C, C-9a), 126.9 (1C, C-4_benzyl_), 127.5 (2C, C-2_benzyl_, C-6_benzyl_), 128.3 (2C, C-3_benzyl_, C-5_benzyl_), 131.2 (1C, C-4a), 136.1 (1C, C-4_TMP_), 136.3 (1C, C-1_TMP_), 137.0 (1C, C-5a), 139.0 (1C, C-1_benzyl_), 152.2 (2C, C-3_TMP_, C-5_TMP_), 170.4 (1C, CO_amide_), 178.1 (1C, C-1), 180.3 (1C, C-3). Exact mass (ESI): *m/z* = 540.2125 (calcd. 540.2129 for C_31_H_30_N_3_O_6_ [MH]^+^). IR (neat): ṽ [cm^−1^] = 2978, 2889 (s, C-H_aliph_.), 1712 (s, C=O), 1153,1122 (s, C-O), 741 (m, C-H_arom._).

**(3a*RS*,4*SR*,10*RS*,10a*RS*)-*N*-(4-Chlorobenzyl)-1,3-dioxo-10-(3,4,5-trimethoxyphenyl)-1,2,3,3a,4,5,10,10a-octahydropyrrolo[3,4-*b*]carbazole-4-carboxamide (13)**

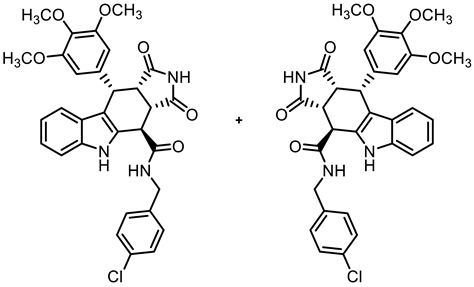

Carboxylic acid **10** (135 mg, 0.30 mmol), COMU^®^ (155 mg, 0.36 mmol), and DIPEA (86.1 mg, 0.67 mmol) were dissolved in dry THF (10 mL) at 0 °C. Then, a solution of 4-chlorobenzylamine (51.2 mg, 0.36 mmol) in THF (2 mL) was added dropwise. The mixture was stirred for 30 min at 0 °C and 1 h at room temperature. After the completion of the transformation, ethyl acetate (30 mL) was added. The mixture was extracted with HCl solution (1 M in water, 5 × 30 mL) and saturated NaCl solution (2 × 25 mL). The organic layer was dried (Na_2_SO_4_), filtered, and concentrated in vacuo. The residue was adsorbed onto silica and purified by automatic flash column chromatography (cartridge: SNAP 50g, flow rate of 50 mL/min, ethyl acetate/cyclohexane = 30:70 → 0:100). Yellow solid, mp 204 °C, yield of 123 mg (71%). C_31_H_28_ClN_3_O_6_ (574.0). TLC (ethyl acetate/cyclohexane = 6:4): R_f_ = 0.38. Purity (HPLC): 96.9%, (t_R_ = 20.3 min). ^1^H NMR (400 MHz, DMSO-*d_6_*): δ (ppm) = 3.56 (s, 3H, 4-OCH_3_), 3.56 (dd, *J* = 9.6/8.0 Hz, 1H, 10a-H), 3.64 (s, 6H, 3-OCH_3_, 5-OCH_3_), 4.00 (dd, *J* = 9.5/4.9 Hz, 1H, 3a-H), 4.25 (dd*, J* = 15.4/4.4 Hz, 1H, PhC*H_2_*NH), 4.46 (d, *J* = 4.9 Hz, 1H, 4-H), 4.71 (dd, *J* = 15.4/7.1 Hz, 1H, Ph*CH_2_*NH), 4.76 (d, *J* = 8.0 Hz, 1H, 10-H), 6.28 (s, 2H, 2-H_TMP_, 6-H_TMP_), 6.91 (ddd, *J* = 8.0/7.0/1.0 Hz, 1H, 8-H), 7.04 (ddd, *J* = 8.2/7.0/1.2 Hz, 1H, 7-H), 7.34 (d, *J* = 7.8 Hz, 1H, 9-H), 7.37 (d, *J* = 8.0 Hz, 1H, 6-H), 7.40–7.45 (m, 4H, 2-H_benzyl_, 3-H_benzyl_, 5-H_benzyl_, 6-H_benzyl_), 9.11 (dd, *J* = 7.1/4.5 Hz, 1H, NH_amide_), 10.75 (s, 1H, 2-H), 10.92 (s, 1H, 5-H). ^13^C NMR (101 MHz, DMSO-*d_6_*): δ (ppm) = 38.0 (1C, C-10), 38.7 (1C, C-4), 42.4 (1C, Ph*C*H_2_NH), 42.9 (1C, C-3a), 46.1 (1C, C-10a), 55.7 (2C, 3-OCH_3,_ 5-OCH_3_), 59.9 (1C, 4-OCH_3_), 105.8 (2C, C-2_TMP_, C-6_TMP_), 111.3 (1C, C-9b) 111.7 (1C, C-6), 118.0 (1C, C-9), 118.7 (1C, C-8), 121.3 (1C, C-7), 125.5 (1C, C-9a), 128.2 (2C, C-2_benzyl_, C-6_benzyl_), 129.3 (2C, C-3_benzyl_, C-5_benzyl_), 131.1 (1C, C-4a), 131.4 (1C, C-4_benzyl_), 136.1 (1C, C-4_TMP_), 136.3 (1C, C-1_TMP_), 137.0 (1C, C-5a), 138.1 (1C, C-1_benzyl_), 152.2 (2C, C-3_TMP_, C-5_TMP_), 170.5 (1C, CO_amide_), 178.1 (1C, C-1), 180.3 (1C, C-3). Exact mass (ESI): *m/z* = 574.1730 (calcd. 574.1739 for C_31_H_29_ClN_3_O_6_ [MH]^+^). IR (neat): ṽ [cm^−1^] = 2970, 2940 (s, C-H_aliph_.), 1717, 1682 (s, C=O), 1173, 1126 (s, C-O), 745 (m, C-H_arom._).

**(3a*RS*,4*SR*,10*RS*,10a*RS*)-*N*-[2-(Indol-3-yl)ethyl]-1,3-dioxo-10-(3,4,5-trimethoxyphenyl)-1,2,3,3a,4,5,10,10a-octahydropyrrolo[3,4-*b*]carbazole-4-carboxamide (14)**

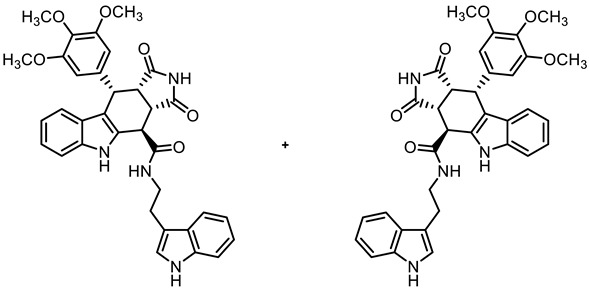

Carboxylic acid **10** (136 mg, 0.30 mmol), COMU^®^ (154 mg, 0.36 mmol), and DIPEA (117 mg, 0.90 mmol) were dissolved in ethyl acetate (10 mL) at room temperature. Then, 2-(indol-3-yl)ethanamine (58.4 mg, 0.36 mmol) was added to the solution. The mixture was stirred for 4 h at room temperature. After the completion of the transformation, ethyl acetate (30 mL) was added. The mixture was extracted with HCl solution (1 M in water, 3 × 20 mL) and saturated NaCl solution (2 × 25 mL). The organic layer was dried (Na_2_SO_4_), filtered, and concentrated in vacuo. The residue was purified by automatic flash column chromatography (cartridge: SNAP 50 g, flow rate of 50 mL/min, ethyl acetate/cyclohexane = 35:65 → 0:100) to obtain a yellow solid. For further purification, the solid was recrystallized (ethyl acetate/cyclohexane), filtered, and washed with cold ethanol (2 × 10 mL). Colorless solid, mp 182 °C, yield of 93 mg (52%). C_34_H_32_N_4_O_6_ (592.7). TLC (ethyl acetate/cyclohexane = 7:3): R_f_ = 0.33. Purity (HPLC): 99.4%, (t_R_ = 19.5 min). ^1^H NMR (400 MHz, DMSO-*d_6_*): δ (ppm) = 3.01 (t, *J* = 7.6 Hz, 2H, NHCH_2_C*H_2_*), 3.40–3.47 (m, 1H, NHC*H_2_*CH_2_), 3.53 (dd, *J* = 9.5/8.0 Hz, 1H, 10a-H), 3.56 (s, 3H, 4-OCH_3_), 3.65 (s, 6H, 3-OCH_3_, 5-OCH_3_), 3.62–3.68 (m, 1H, NHC*H_2_*CH_2_), 3.99 (dd, *J* = 9.4/4.6 Hz, 1H, 3a-H), 4.42 (d, *J* = 4.6 Hz, 1H, 4-H), 4.74 (d, *J* = 8.0 Hz, 1H, 10-H), 6.27 (s, 2H, 2-H_TMP_, 6-H_TMP_) 6.89 (ddd, *J* = 7.9/7.0/1.0 Hz, 1H, 8-H), 7.00 (ddd, *J* = 7.9/7.0/1.1 Hz, 1H, 5-H_indolyl_), 7.02 (ddd, *J* = 8.2/7.0/1.2 Hz, 1H, 7-H), 7.09 (ddd, *J* = 8.1/6.9/1.2 Hz, 1H, 6-H_indolyl_), 7.24 (d, *J* = 2.2 Hz, 1H, 2-H_indolyl_), 7.31 (dt, *J* = 8.2/0.8 Hz, 1H, 6-H), 7.32 (d, *J* = 8.0 Hz, 1H, 9-H), 7.36 (dt, *J* = 8.1/0.9 Hz, 1H, 7-H_indolyl_), 7.62 (d, *J* = 7.9 Hz, 1H, 4-H_indolyl_), 8.73 (t, *J* = 5.6 Hz, 1H, NH_amide_), 10.42 (s, 1H, 5-H), 10.87 (d, *J* = 2.4 Hz, 1H, 1-H_indolyl_), 10.89 (s, 1H, 2-H). ^13^C NMR (101 MHz, DMSO-*d_6_*): δ (ppm) = 25.0 (1C, NHCH_2_*C*H_2_), 38.1 (1C, C-10), 38.5 (1C, C-4), 40.3 (1C, NH*C*H_2_CH_2_), 42.7 (1C, C-3a), 46.1 (1C, C-10a), 55.7 (2C, 3-OCH_3_, 5-OCH_3_), 59.9 (1C, 4-OCH_3_), 105.9 (2C, C-2_TMP_, C-6_TMP_), 111.4 (1C, C-6), 111.4 (1C, C-7_indolyl_), 111.6 (1C, C-9b), 111.7 (1C, C-3_indolyl_), 117.9 (1C, C-9), 118.3 (1C, C-5_indolyl_), 118.3 (1C, C-4_indolyl_), 118.7 (1C, C-8), 121.0 (1C, C-6_indolyl_), 121.3 (1C, C-7), 122.7 (1C, C-2_indolyl_), 125.6 (1C, C-9a), 127.2 (1C, C-3a_indolyl_), 131.2 (1C, C-4a), 136.1 (1C, C-4_TMP_), 136.2 (1C, C-5a), 136.3 (1C, C-7a_indolyl_), 136.9 (1C, C-1_TMP_), 152.2 (2C, C-3_TMP_, C-5_TMP_), 170.1 (1C, CO_amide_), 178.1 (1C, C-1), 180.3 (1C, C-3). Exact mass (ESI): *m/z* = 593.2370 (calcd. 593.2395 for C_34_H_33_N_4_O_6_ [MH]^+^). IR (neat): ṽ [cm^−1^] = 2978, 2940 (s, C-H_aliph_.), 1701 (s, C=O), 1177, 1123 (s, C-O), 733 (s, C-H_arom._).

**(3a*RS*,4*SR*,10*RS*,10a*RS*)-1,3-Dioxo-*N*-(pyridin-3-yl)-10-(3,4,5-trimethoxyphenyl)-1,2,3,3a,4,5,10,10a-octahydropyrrolo[3,4-*b*]carbazole-4-carboxamide (15)**

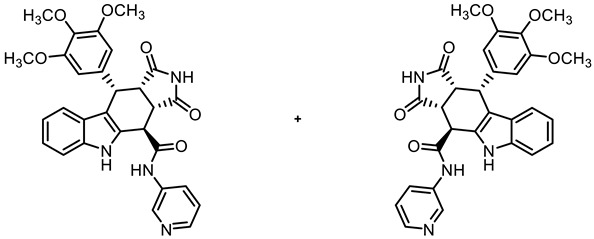

Carboxylic acid **10** (135 mg, 0.30 mmol), COMU^®^ (155 mg, 0.36 mmol), and DIPEA (85.5 mg, 0.66 mmol) were dissolved in dry THF (10 mL) at room temperature. Then, pyridin-3-amine (37.7 mg, 0.40 mmol) was added to the solution. The mixture was stirred for 12 h at room temperature. After the completion of the transformation, ethyl acetate (50 mL) was added. The mixture was extracted with HCl solution (1 M in water, 5 × 20 mL) and saturated NaCl solution (2 × 20 mL). The organic layer was dried (Na_2_SO_4_), filtered, and concentrated in vacuo. The residue was purified by automatic flash column chromatography (cartridge: SNAP 50g, flow rate of 50 mL/min, MeOH/CH_2_Cl_2_ = 0:100 → 10:90). Colorless solid, mp 198 °C, yield of 21 mg (12%). C_29_H_26_N_4_O_6_ (526.5). TLC (MeOH/CH_2_Cl_2_ = 5:95): R_f_ = 0.14. Purity (HPLC): 97.6%, (t_R_ = 15.7 min). ^1^H NMR (400 MHz, DMSO-*d_6_*): δ (ppm) = 3.57 (s, 3H, 4-OCH_3_), 3.60 (dd, *J* = 9.6/8.0 Hz, 1H, 10a-H), 3.68 (s, 6H, 3-OCH_3_, 5-OCH_3_), 4.14 (dd, *J* = 9.5/4.9 Hz, 1H, 3a-H), 4.67 (d, *J* = 5.0 Hz, 1H, 4-H), 4.81 (d, *J* = 7.9 Hz, 1H, 10-H), 6.31 (s, 2H, 2-H_TMP_, 6-H_TMP_), 6.92 (ddd, *J* = 7.9/7.1/1.0 Hz, 1H, 8-H), 7.04 (ddd, *J* = 8.2/7.0/1.2 Hz, 1H, 7-H), 7.34 (d, *J* = 8.1 Hz, 1H, 6-H), 7.40 (d, *J* = 7.9 Hz, 1H, 9-H), 7.45 (dd, *J* = 8.3/4.7 Hz, 1H, 5-H_pyridinyl_), 8.22 (ddd, *J* = 8.3/2.6/1.5 Hz, 1H, 6-H_pyridinyl_), 8.36 (dd, *J* = 4.7/1.5 Hz, 1H, 4-H_pyridinyl_), 8.93 (d, *J* = 2.5 Hz, 1H, 2-H_pyridinyl_), 10.95 (s, 1H, 5-H), 10.97 (s, 1H, 2-H), 11.02 (s, 1H, NH_amide_). ^13^C NMR (101 MHz, DMSO-*d_6_*): δ (ppm) = 38.0 (1C, C-10), 42.8 (1C, C-3a), 46.0 (1C, C-10a), 55.8 (2C, 3-OCH_3_, 5-OCH_3_), 59.9 (1C, 4-OCH_3_), 105.9 (2C, C-2_TMP_, C-6_TMP_), 111.3 (1C, C-9b), 112.0 (1C, C-6), 118.0 (1C, C-9), 118.8 (1C, C-8), 121.5 (1C, C-7), 123.7 (1C, C-5_pyridinyl_), 125.4 (1C, C-9a), 126.7 (1C, C-6_pyridinyl_), 130.4 (1C, C-4a), 135.9 (1C, C-3_pyridinyl_), 136.2 (1C, C-4_TMP_), 136.4 (1C, C-5a), 137.0 (1C, C-1_TMP_), 141.2 (1C, C-2_pyridinyl_), 144.5 (1C, C-4_pyridinyl_), 152.3 (2C, C-3_TMP_, C-5_TMP_), 169.7 (1C, CO_amide_), 178.0 (1C, C-1), 180.3 (1C, C-3). The signal for C-4 C-atom is overlaid by the DMSO signals at 39.5 ppm. Exact mass (ESI): *m/z* = 527.1919 (calcd. 527.1925 for C_29_H_27_N_4_O_6_ [MH]^+^). IR (neat): ṽ [cm^−1^] = 2978, 2932 (s, C-H_aliph_.), 1709 (s, C=O), 1173, 1123 (s, C-O), 745 (s, C-H_arom._).

**(3a*RS*,4*SR*,10*RS*,10a*RS*)-4-[(Piperidin-1-yl)carbonyl]-10-(3,4,5-trimethoxyphenyl)-4,5,10,10a-tetrahydropyrrolo[3,4-*b*]carbazole-1,3(2*H*,3a*H*)-dione (16)**

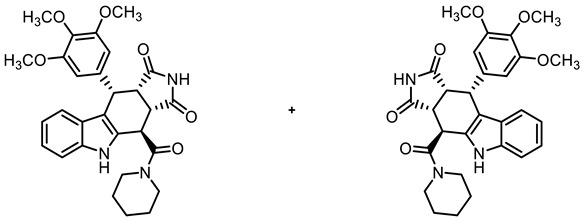

Carboxylic acid **10** (136 mg, 0.30 mmol), COMU^®^ (168 mg, 0.39 mmol), and DIPEA (89.6 mg, 0.69 mmol) were dissolved in dry THF (10 mL) at 0 °C. Then, a solution of piperidine (32.2 mg, 0.38 mmol) in THF (3 mL) was added dropwise. The mixture was stirred for 2 h at 0 °C. After the completion of the transformation, the solution was allowed to warm to ambient temperature and ethyl acetate (30 mL) was added. The mixture was extracted with NaHCO_3_ solution (0.1 M in water, 3 × 25 mL), HCl solution (1 M in water, 3 × 25 mL), and saturated NaCl solution (1 × 25 mL). The organic layer was dried (Na_2_SO_4_), filtered, and concentrated in vacuo. The residue was purified by flash column chromatography (ethyl acetate/cyclohexane = 6:4 → 7:3, Ø 2 cm, h = 25 cm, v = 10 mL). Colorless solid, mp 293 °C, yield of 103 mg (66%). C_29_H_31_N_3_O_6_ (517.6). TLC (ethyl acetate/cyclohexane = 6:4): R_f_ = 0.29. Purity (HPLC): 96.7%, (t_R_ = 18.4 min). ^1^H NMR (600 MHz, DMSO-*d_6_*): δ (ppm) = 1.43–1.74 (m, 6H, N(CH_2_C*H_2_*)_2_C*H_2_*), 3.22 (ddd, *J* = 12.8/9.7/3.0 Hz, 1H, N(C*H_2_*CH_2_)_2_CH_2_), 3.51 (dd, *J* = 9.3/8.0 Hz, 1H, 10a-H), 3.57 (s, 3H, 4-OCH_3_), 3.59–3.62 (m, 1H, N(C*H_2_*CH_2_)_2_CH_2_), 3.63 (s, 6H, 3-OCH_3_, 5-OCH_3_), 3.92 (dt, *J* = 13.7/4.2 Hz, 1H, N(C*H_2_*CH_2_)_2_CH_2_), 3.97 (dd, *J* = 9.1/4.3 Hz, 1H, 3a-H), 3.95–4.02 (m, 1H, N(C*H_2_*CH_2_)_2_CH_2_), 4.76 (d, *J* = 8.3 Hz, 1H, 10-H), 4.77 (d, *J* = 4.0 Hz, 1H, 4-H), 6.28 (s, 2H, 2-H_TMP_, 6-H_TMP_), 6.89 (ddd, *J* = 7.9/7.0/1.0 Hz, 1H, 8-H), 7.03 (ddd, *J* = 8.1/7.0/1.2 Hz, 1H, 7-H), 7.27 (dd, *J* = 8.0/1.0 Hz, 1H, 9-H), 7.33 (dd, *J* = 8.1/0.9 Hz, 1H, 6-H), 10.78 (s, 1H, 5-H), 10.91 (s, 1H, 2-H). ^13^C NMR (101 MHz, DMSO-*d_6_*): δ (ppm) = 24.2 (1C, N(CH_2_CH_2_)_2_*C*H_2_) 25.3 (1C, N(CH_2_*C*H_2_)_2_CH_2_), 26.0 (1C, N(CH_2_*C*H_2_)_2_CH_2_), 34.3 (1C, C-4), 38.3 (1C, C-10), 43.3 (1C, N(*C*H_2_CH_2_)_2_CH_2_), 44.0 (1C, C-3a), 46.3 (1C, C-10a), 47.0 (1C, N(*C*H_2_CH_2_)_2_CH_2_), 55.5 (2C, 3-OCH_3_, 5-OCH_3_), 59.9 (1C, 4-OCH_3_), 105.9 (2C, C-2_TMP_, C-6_TMP_), 111.3 (1C, C-6), 111.5 (1C, C-9b), 118.0 (1C, C-9), 118.6 (1C, C-8), 121.2 (1C, C-7), 125.5 (1C, C-9a), 131.1 (1C, C-4a), 136.1 (1C, C-4_TMP_), 136.4 (1C, C-5a), 136.6 (1C, C-1_TMP_), 152.2 (2C, C-3_TMP_, C-5_TMP_), 168.5 (1C, CO_amide_), 178.0 (1C, C-1), 180.2 (1C, C-3). Exact mass (ESI): *m/z* = 518.2280 (calcd. 518.2286 for C_29_H_32_N_3_O_6_ [MH]^+^). IR (neat): ṽ [cm^−1^] = 2978, 2886 (s, C-H_aliph_.), 1709 (s, C=O), 1150, 1119 (s, C-O), 799, 745 (s, C-H_arom._).

**(3a*RS*,4*SR*,10*RS*,10a*RS*)-*N*-(1*H*-Benzimidazol-2-yl)-1,3-dioxo-10-(3,4,5-trimethoxyphenyl)-1,2,3,3a,4,5,10,10a-octahydropyrrolo[3,4-*b*]carbazole-4-carboxamide (17)**

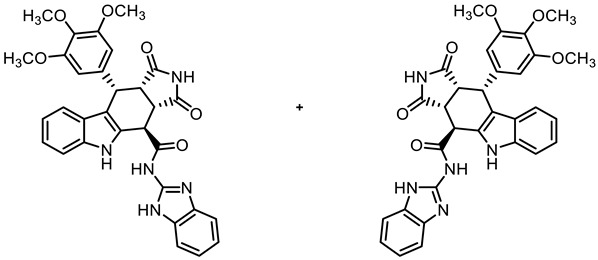

Carboxylic acid **10** (136 mg, 0.30 mmol), COMU^®^ (167 mg, 0.39 mmol), and DIPEA (128 mg, 0.99 mmol) were dissolved in dry THF (10 mL) at room temperature. Then, benzimidazol-2-amine (52.2 mg, 0.39 mmol) was added. The mixture was stirred for 2 h at room temperature. After the completion of the transformation, ethyl acetate (50 mL) was added. The mixture was extracted with saturated NH_4_Cl solution (3 × 25 mL), NaHCO_3_ solution (0.1 M in water, 3 × 25 mL), and saturated NaCl solution (1 × 25 mL). The organic layer was dried (Na_2_SO_4_), filtered, and concentrated in vacuo. The residue was dissolved in ethyl acetate (10 mL), and *n*-hexane (30 mL) was added. The resulting mixture was filtered, and the precipitate was washed with cold Et_2_O. Colorless solid, mp 233 °C, yield of 39 mg (23%). C_31_H_27_N_5_O_6_ (565.6). TLC (ethyl acetate/cyclohexane = 6:4): R_f_ = 0.16. Purity (HPLC): 97.3%, (t_R_ = 17.7 min). ^1^H NMR (600 MHz, DMSO-*d_6_*): δ (ppm) = 3.57 (s, 3H, 4-OCH_3_), 3.65 (dd, *J* = 9.8/7.9 Hz, 1H, 10a-H), 3.70 (s, 6H, 3-OCH_3_, 5-OCH_3_), 4.19 (dd, *J* = 9.7/5.8 Hz, 1H, 3a-H), 4.79 (d, *J* = 5.8 Hz, 1H, 4-H), 4.83 (d, *J* = 8.0 Hz, 1H, 10-H), 6.34 (s, 2H, 2-H_TMP_, 6-H_TMP_), 6.93 (ddd, *J* = 7.9/6.9/1.0 Hz, 1H, 8-H), 7.03 (ddd, *J* = 8.1/6.9/1.2 Hz, 1H, 7-H), 7.11–7.16 (m, 2H, 5-H_BI_, 6-H_BI_), 7.30–7.34 (m, 1H, 6-H), 7.44 (d, *J* = 7.9 Hz, 1H, 9-H), 7.50–7.54 (m, 2H, 4-H_BI_, 7-H_BI_), 11.03 (s, 1H, 5-H), 11.03 (s, 1H, 2-H), 12.20 (s, 2H, NH_amide_, 1-H_BI_). ^13^C NMR (101 MHz, DMSO-*d_6_*): δ (ppm) = 38.1 (1C, C-10), 42.9 (1C, C-3a), 46.1 (1C, C-10a), 55.7 (2C, 3-OCH_3_, 5-OCH_3_), 59.9 (1C, 4-OCH_3_), 105.5 (2C, C-2_TMP_, C-6_TMP_), 111.4 (1C, C-6), 112.1 (1C, C-9b), 117.9 (1C, C-9), 118.8 (1C, C-8), 121.3 (2C, C-5_BI_, C-6_BI_), 121.4 (1C, C-7), 125.4 (1C, C-9a), 130.4 (1C, C-4a), 136.1 (1C, C-4_TMP_), 136.3 (1C, C-5a), 137.1 (1C, C-1_TMP_), 152.3 (2C, C-3_TMP_, C-5_TMP_), 172.0 (1C, CO_amide_), 178.1 (1C, C-1), 180.3 (1C, C-3). Signals for C-3a_BI_, C-4_BI_, C-7_BI_, and C-7a_BI_ C-atoms were not observed in the spectrum. The signal for C-4 C-atom was overlaid by the DMSO signal at 39.5 ppm. Exact mass (ESI): *m/z* = 566.2037 (calcd. 566.2034 for C_31_H_28_N_5_O_6_ [MH]^+^). IR (neat): ṽ [cm^−1^] = 2909, 2835 (w, C-H_aliph_.), 1701 (s, C=O), 1177, 1126 (s, C-O), 798, 745 (s, C-H_arom._).

**Methyl *N*-{[(3a*S*,4*R*,10*S*,10a*S*)-1,3-dioxo-10-(3,4,5-trimethoxyphenyl)-1,2,3,3a,4,5,10,10a-octahydropyrrolo[3,4-*b*]carbazol-4-yl]carbonyl}-(*S*)-serinate ((+)-18a) and methyl *N*-{[(3a*R*,4*S*,10*R*,10a*R*)-1,3-dioxo-10-(3,4,5-trimethoxyphenyl)-1,2,3,3a,4,5,10,10a-octahydropyrrolo[3,4-*b*]carbazol-4-yl]carbonyl}-(*S*)-serinate ((-)-18b)**

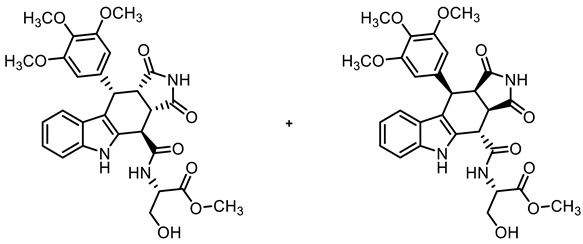

Carboxylic acid **10** (136 mg, 0.30 mmol), COMU^®^ (168 mg, 0.39 mmol), and DIPEA (128 mg, 0.99 mmol) were dissolved in dry DMF (10 mL) at 0 °C. After the addition of (*S*)-serine methyl ester HCl (38.9 mg, 0.25 mmol), the mixture was stirred for 2 h at 0 °C. After the completion of the transformation, the solution was allowed to warm to ambient temperature, and ethyl acetate (30 mL) was added. The mixture was washed with HCl solution (1 M in water, 3 × 25 mL) and saturated NaCl solution (1 × 25 mL). The organic layer was dried (Na_2_SO_4_), filtered, and concentrated in vacuo. The residue was purified by flash column chromatography (ethyl acetate/cyclohexane = 8:2, Ø 2 cm, h = 15 cm, v = 10 mL) to give both diastereomers.
**(+)-18a** [R_f_ = 0.41 (ethyl acetate/cyclohexane = 8:2)]: Yellow solid, mp 174 °C, yield of 37 mg (27%). C_28_H_29_N_3_O_9_ (551.6). Purity (HPLC): 93.7%, (t_R_ = 16.5 min). Specific rotation: ${\left[\alpha \right]}_{D}^{20}=$ +100 (c = 1.7, THF). ^1^H NMR (600 MHz, DMSO-*d_6_*): δ (ppm) = 3.51 (dd, *J* = 9.6/7.8 Hz, 1H, 10a-H), 3.56 (s, 3H, 4-OCH_3_), 3.68 (s, 6H, 3-OCH_3_, 5-OCH_3_), 3.78 (s, 3H, CO_2_CH_3_), 3.78–3.84 (m, 1 H, CHC*H_2_*OH), 3.97 (dt, *J* = 11.0/5.1 Hz, 1H, CHC*H_2_*OH), 4.08 (dd, *J* = 9.6/5.2 Hz, 1H, 3a-H), 4.60–4.69 (m, 1H, C*H*CH_2_OH), 4.67 (d, *J* = 5.3 Hz, 1H, 4-H), 4.76 (d, *J* = 7.8 Hz, 1H, 10-H), 5.41 (t, *J* = 5.2 Hz, 1H, CHCH_2_O*H*), 6.33 (s, 2H, 2-H_TMP_, 6-H_TMP_), 6.95 (ddd, *J* = 7.9/7.0/1.0 Hz, 1H, 8-H), 7.08 (ddd, *J* = 8.1/7.0/1.2 Hz, 1H, 7-H), 7.34 (dt, *J* = 8.0/0.9 Hz, 1H, 6-H), 7.42 (dd, *J* = 7.9/1.0 Hz, 1H, 9-H), 9.35 (d, *J* = 7.9 Hz, 1H), NH_amide_), 10.38 (s, 1H, 5-H), 10.93 (s, 1H, 2-H). ^13^C NMR (151 MHz, DMSO-*d_6_*): δ (ppm) = 38.1 (1C, C-10), 38.2 (1C, C-4), 41.7 (1C, C-3a), 46.3 (1C, C-10a), 52.7 (1C, CO_2_*C*H_3_), 55.3 (1C, *C*HCH_2_OH), 55.8 (2C, 3-OCH_3_, 5-OCH_3_), 59.9 (1C, 4-OCH_3_), 61.0 (1C, CH*C*H_2_OH_)_, 105.8 (2C, C-2_TMP_, C-5_TMP_), 111.0 (1C, C-6), 112.0 (1C, C-9b), 118.1 (1C, C-9), 119.0 (1C, C-8), 121.6 (1C, C-7), 125.6 (1C, C-9a), 131.3 (1C, C-4a), 135.7 (1C, C-5a), 136.2 (1C, C-4_TMP_), 136.9 (1C, C-1_TMP_), 152.3 (2C, C-3_TMP_, C-5_TMP_), 170.3 (1C, *C*O_2_CH_3_), 172.7 (C-4-C=O), 178.1 (1C, C-1), 180.5 (1C, C-3). Exact mass (ESI): *m/z* = 552.1972 (calcd. 552.1977 for C_28_H_30_N_3_O_9_ [MH]^+^). IR (neat): ṽ [cm^−1^] = 3314 (m, OH), 2931, 2847 (w, C-H_aliph_.), 1708, 1674 (s, C=O), 1123 (s, C-O), 745 (s, C-H_arom._).
**(−)-18b** [R_f_ = 0.35 (ethyl acetate/cyclohexane = 8:2)]: Yellow solid, mp 183 °C, yield of 37 mg (27%). C_28_H_29_N_3_O_9_ (551.6). Purity (HPLC): 93.2%, (t_R_ = 16.0 min). Specific rotation: ${\left[\alpha \right]}_{D}^{20}=$ −115 (c = 1.3, THF). ^1^H NMR (600 MHz, DMSO-*d_6_*): δ (ppm) = 3.50 (dd, *J* = 9.5/7.8 Hz, 1H, 10a-H), 3.56 (s, 3H, 4-OCH_3_), 3.66 (s, 6H, 3-OCH_3_, 5-OCH_3_), 3.71 (s, 3H, CO_2_CH_3_), 3.82–3.89 (m, 1H, CHC*H_2_*OH), 3.94 (dt, *J* = 10.8/4.7 Hz, 1H, CHC*H_2_*OH), 4.03 (dd, *J* = 9.7/4.4 Hz, 1H, 3a-H), 4.62 (d, *J* = 4.6 Hz, 1H, 4-H), 4.62–4.65 (m, 1H, C*H*CH_2_OH), 4.75 (d, *J* = 7.8 Hz, 1H, 10-H), 5.83 (t, *J* = 5.3 Hz, 1H, CHCH_2_O*H*), 6.29 (s, 2H, C-2_TMP_, C-6_TMP_), 6.92 (ddd, *J* = 8.0/7.0/1.0 Hz, 1H, 8-H), 7.05 (ddd, *J* = 8.2/6.9/1.2 Hz, 1H, 7-H), 7.30 (dt, *J* = 8.1/0.9 Hz, 1H, 6-H), 7.36 (d, *J* = 7.9 Hz, 1H, 9-H), 9.15 (d, *J* = 8.1 Hz, 1H, NH_amide_), 10.58 (s, 1H, 5-H), 10.90 (s, 1H, 2-H). ^13^C NMR (151 MHz, DMSO-*d_6_*): δ (ppm) = 38.0 (1C, C-10), 38.2 (1C, C-4), 41.9 (1C, C-3a), 46.3 (1C, C-10a), 52.1 (1C, CO_2_*C*H_3_), 55.1 (1C, *C*HCH_2_OH), 55.7 (2C, 3-OCH_3_, 5-OCH_3_), 59.9 (1C, 4-OCH_3_), 60.8 (1C, CH*C*H_2_OH_)_, 105.8 (2C, C-2_TMP_, C-6_TMP_), 111.0 (1C, C-6), 111.7 (1C, C-9b), 118.0 (1C, C-9), 118.8 (1C, C-8), 121.4 (1C, C-7), 125.6 (1C, C-9a), 130.9 (1C, C-4a), 136.0 (1C, C-5a), 136.1 (1C, C-4_TMP_), 136.8 (1C, C-1_TMP_), 152.2 (2C, C-3_TMP_, C-5_TMP_), 170.5 (1C, C-4-*C*=O), 170.6 (*C*O_2_CH_3_), 178.1 (1C, C-1), 180.3 (1C, C-3). Exact mass (ESI): *m/z* = 552.1972 (calcd. 552.1977 for C_28_H_30_N_3_O_9_ [MH]^+^). IR (neat): ṽ [cm^−1^] = 3309 (m, OH), 2927, 2850 (w, C-H_aliph_.), 1708, 1674 (s, C=O), 1123 (s, C-O), 745 (s, C-H_arom._).

**Methyl *N*-{[(3a*R*,4*S*,10*R*,10a*R*)-1,3-dioxo-10-(3,4,5-trimethoxyphenyl)-1,2,3,3a,4,5,10,10a-octahydropyrrolo[3,4-*b*]carbazol-4-yl]carbonyl}-(*S*)-methioninate ((-)-19b)**

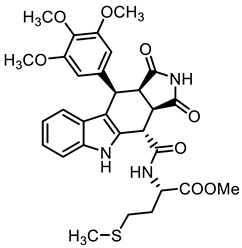

Under N_2_, carboxylic acid **10** (136 mg, 0.30 mmol) and CDI (53.5 mg, 0.33 mmol) were dissolved in dry CH_2_Cl_2_ (10 mL) at room temperature. The mixture was stirred for 2 h. Then, (*S*)-methionine methyl ester HCl (120 mg, 0.60 mmol) was added. The mixture was stirred for 48 h at room temperature. After the completion of the transformation, the resulting suspension was filtered through Celite^®^, followed by rinsing with CH_2_Cl_2_ (40 mL). The filtrate was concentrated in vacuo and the residue was purified by flash column chromatography (ethyl acetate/cyclohexane = 6:4, Ø 2 cm, h = 20 cm, v = 10 mL). Yellow solid, mp 127 °C, yield of 39 mg (22%). C_30_H_33_N_3_O_8_S (595.7). TLC (ethyl acetate/cyclohexane = 6:4): R_f_ = 0.24. Purity (HPLC): 98.1%, (t_R_ = 19.6 min). Specific rotation: ${\left[\alpha \right]}_{D}^{20}=$ −113 (c = 1.2, DMSO). ^1^H NMR (600 MHz, DMSO-*d_6_*): δ (ppm) = 2.02–2.07 (m, 1H, CH_3_SCH_2_C*H_2_*CH), 2.09 (s, 3H, SCH_3_), 2.11–2.17 (m, 1H, CH_3_SCH_2_C*H_2_*CH), 2.58–2.69 (m, 2H, CH_3_SC*H_2_*CH_2_CH), 3.55 (dd, *J* = 9.7/8.0 Hz, 1H, 10a-H), 3.57 (s, 3H, 4-OCH_3_), 3.67 (s, 6H, 3-OCH_3_, 5-OCH_3_), 3.76 (s, 3H, CO_2_CH_3_), 4.04 (dd, *J* = 9.6/5.3 Hz, 1H, 3a-H), 4.52 (d, *J* = 5.3 Hz, 1H, 4-H), 4.69 (ddd, *J* = 9.0/7.6/4.7 Hz, 1H, CH_3_SCH_2_CH_2_C*H*), 4.78 (d, *J* = 8.0 Hz, 1H, 10-H), 6.32 (s, 2H, 2-H_TMP_,6-H_TMP_), 6.95 (ddd, *J* = 8.0/7.0/1.0 Hz, 1H, 8-H), 7.07 (ddd, *J* = 8.2/7.0/1.2 Hz, 1H, 7-H), 7.36 (dt, *J* = 8.1/0.9 Hz, 1H, 6-H), 7.42 (d, *J* = 8.0 Hz, 1H, 9-H), 9.18 (d, *J* = 7.7 Hz, 1H, NH_amide_), 10.34 (s, 1H, 5-H), 10.97 (s, 1H, 2-H). ^13^C NMR (101 MHz, DMSO-*d_6_*): δ (ppm) = 14.6 (1C, SCH_3_), 29.5 (1C, CH_3_S*C*H_2_CH_2_CH), 30.7 (1C, CH_3_SCH_2_*C*H_2_CH), 38.0 (1C, C-10), 38.4 (1C, C-4), 42.1 (1C, C-3a), 46.0 (1C, C-10a), 51.6 (1C, CH_3_SCH_2_CH_2_*C*H), 52.7 (1C, CO_2_*C*H_3_), 55.8 (2C, 3-OCH_3_, 5-OCH_3_), 59.9 (1C, 4-OCH_3_), 105.8 (2C, C-2_TMP_, C-6_TMP_), 111.1 (1C, C-6), 112.1 (1C, C-9b), 118.1 (1C, C-9), 119.0 (1C, C-8), 121.6 (1C, C-7), 125.6 (1C, C-9a), 130.8 (1C, C-4a), 135.9 (1C, C-5a), 136.2 (1C, C-4_TMP_), 137.0 (1C, C-1_TMP_), 152.3 (2C, C-3_TMP_, C-5_TMP_), 170.3 (1C, CO_amide_), 173.5 (1C, *C*O_2_CH_3_), 178.1 (1C, C-1), 180.3 (1C, C-3). Exact mass (ESI): *m/z* = 596.2062 (calcd. 566.2061 for C_30_H_34_N_3_O_8_S [MH]^+^). IR (neat): ṽ [cm^−1^] = 2978, 2889 (w, C-H_aliph_.), 1774, (w, C=O), 1713 (s, C=O), 1643 (w, C=O), 1173, 1123 (s, C-O), 745 (s, C-H_arom._).

**Methyl *N*-{[(3a*S*,4*R*,10*S*,10a*S*)-1,3-dioxo-10-(3,4,5-trimethoxyphenyl)-1,2,3,3a,4,5,10,10a-octahydropyrrolo[3,4-*b*]carbazol-4-yl]carbonyl}-(*S*)-histidinate∙HCl ((+(-20a HCl) and methyl *N*-{[(3a*R*,4*S*,10*R*,10a*R*)-1,3-dioxo-10-(3,4,5-trimethoxyphenyl)-1,2,3,3a,4,5,10,10a-octahydropyrrolo[3,4-*b*]carbazol-4-yl]carbonyl}-(*S*)-histidinate ((-)-20b)**

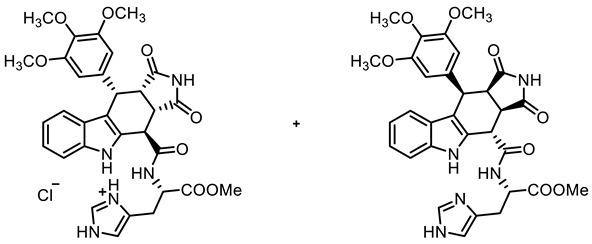

Carboxylic acid **10** (450 mg, 1.00 mmol), COMU^®^ (471 mg, 1.10 mmol), and (*S*)-histidine methyl ester HCl (267 mg, 1.10 mmol) were dissolved in dry THF (20 mL) at room temperature. Then, DIPEA (426 mg, 3.30 mmol) was added dropwise. The mixture was stirred for 16 h at room temperature. After the completion of the transformation, ethyl acetate (100 mL) was added. The mixture was extracted with NaHCO_3_ solution (0.1 M in water, 4 × 50 mL) and saturated NaCl solution (1 × 50 mL). The organic layer was dried (Na_2_SO_4_), filtered, and concentrated in vacuo. The residue was purified by automatic flash column chromatography (cartridge: SNAP 100 g, flow rate of 50 mL/min, MeOH/CH_2_Cl_2_ = 2:98 → 10:90) to obtain the diastereomers **20a** and **20b**.
Then, **20a** was dissolved in CH_2_Cl_2_ (10 mL) and HCl solution (2 M in Et_2_O, 5 mL) was added. The resulting suspension was filtered and the precipitate was washed with cold CH_2_Cl_2_ (2 × 10 mL) to give **20a**∙HCl.
**(+)-20a**∙HCl [R_f_ = 0.43 (MeOH/CH_2_Cl_2_ = 10:90, free base)]: yellow solid, mp 253 °C, yield of 125 mg (20%). C_31_H_32_ClN_5_O_8_ (638.1). Purity (HPLC): 95.0%, (t_R_ = 15.3 min). Specific rotation: ${\left[\alpha \right]}_{D}^{20}=$ +121 (c = 1.7, DMSO). ^1^H NMR (600 MHz, DMSO-*d_6_*): δ (ppm) = 3.27 (dd, *J* = 15.2/6.4 Hz, 1H, NHCHC*H_2_*), 3.34 (dd, *J* = 15.1/8.3 Hz, 1H, NHCHC*H_2_*), 3.53 (t, *J* = 8.9 Hz, 1H, 10a-H), 3.57 (s, 3H, 4-OCH_3_), 3.63 (s, 6H, 3-OCH_3_, 5-OCH_3_), 3.65 (s, 3H, CO_2_CH_3_), 3.85 (dd, *J* = 9.0/3.7 Hz, 1H, 3a-H), 4.52 (d, *J* = 3.8 Hz, 1H, 4-H), 4.67–4.75 (m, 1H, NHC*H*CH_2_), 4.72 (d, *J* = 8.2 Hz, 1H, 10-H), 6.25 (s, 2H, 2-H_TMP_, 6-H_TMP_), 6.88 (ddd, *J* = 8.0/7.1/1.0 Hz, 1H, 8-H), 7.04 (ddd, *J* = 8.1/7.0/1.2 Hz, 1H, 7-H), 7.20 (d, *J* = 7.9 Hz, 1H, 9-H), 7.39 (dt, *J* = 8.1/0.9 Hz, 1H, 6-H), 7.51 (s, 1H, 5-H_imidaz_.), 8.99 (s, 1H, 2-H_imidaz_.), 9.22 (d, *J* = 7.4 Hz, 1H, NH_amide_), 10.52 (s, 1H, 5-H), 10.91 (s, 1H, 2-H). A signal for the NH_imidaz._ proton is not observed in the spectrum. ^13^C NMR (101 MHz, DMSO-*d_6_*): δ (ppm) = 26.0 (1C, NHCH*C*H_2_), 37.7 (1C, C-4), 38.1 (1C, C-10), 42.8 (1C, C-3a), 45.9 (1C, C-10-a), 52.2 (1C, NH*C*HCH_2_), 52.3 (1C, CO_2_*C*H_3_), 55.7 (2C, 3-OCH_3_, 5-OCH_3_), 59.9 (1C, 4-OCH_3_), 106.1 (2C, C-2_TMP_, C-6_TMP_), 111.4 (1C, C-6), 111.4 (1C, C-9b), 117.2 (1C, C-5_imidaz._) 118.2 (1C, C-9), 118.7 (1C, C-8), 121.5 (1C, C-7), 125.6 (1C, C-9a), 130.4 (1C, C-4a), 133.9 (1C, C-2_imidaz._), 136.2 (1C, C-4_TMP_), 136.3 (1C, C-5a), 136.6 (1C, C-1_TMP_), 152.1 (2C, C-3_TMP_, C-5_TMP_), 170.8 (1C, CO_amide_), 171.0 (1C, *C*O_2_CH_3_), 178.0 (1C, C-1), 179.8 (1C, C-3). A signal for the C-4_imidaz._ C-atom was not observed in the spectrum. Exact mass (ESI): *m/z* = 602.2251 (calcd. 602.2245 for C_31_H_32_N_5_O_8_ [MH]^+^). IR (neat): ṽ [cm^−1^] = 2978, 2886 (s, C-H_aliph_.), 1713 (s, C=O), 1686 (m, C=O), 1154, 1119 (s, C-O), 745 (s, C-H_arom._).
**(−)-20b** [R_f_ = 0.32 (MeOH/CH_2_Cl_2_ = 10:90)]: Yellow solid, mp 233 °C, yield of 172 mg (29%). C_31_H_31_N_5_O_8_ (601.6). Purity (HPLC): 94.3%, (t_R_ = 15.9 min). Specific rotation: ${\left[\alpha \right]}_{D}^{20}=$ −169 (c 1.5, DMSO). ^1^H NMR (600 MHz, DMSO-*d_6_*): δ (ppm) = 3.10 (dd, *J* = 14.8/9.3 Hz, 1H, NHCHC*H_2_*), 3.16 (dd, *J* = 14.9/4.2 Hz, 1H, NHCHC*H_2_*), 3.50 (dd, *J* = 9.5/7.8 Hz, 1H, 10a-H), 3.56 (s, 3H, 4-OCH_3_), 3.66 (s, 9H, 3-OCH_3_, 5-OCH_3_, CO_2_CH_3_), 4.03 (dd, *J* = 9.6/4.9 Hz, 1H, 3a-H), 4.29 (s broad, 1H, NHC*H*CH_2_), 4.53 (d, *J* = 5.0 Hz, 1H, 4-H), 4.75 (d, *J* = 7.8 Hz, 1H, 10-H), 6.31 (s, 2H, 2-H_TMP_, 6-H_TMP_), 6.93 (td, *J* = 7.5/7.1/0.9 Hz, 1H, 8-H), 7.03 (s, 1H, 5-H_imidaz._), 7.06 (ddd, *J* = 8.2/7.0/1.1 Hz, 1H, 7-H), 7.37–7.43 (m, 2H, 6-H, 9-H), 7.80 (s, 1H, 2-H_imidaz._), 9.36 (s, 1H, NH_amide_), 10.90 (s, 1H, 2-H), 11.95 (s, 1H, 5-H), 12.07 (s, 1H, τ-NH_imidaz._). ^13^C NMR (101 MHz, DMSO-*d_6_*): δ (ppm) = 27.8 (1C, NHCH*C*H_2_), 38.1 (1C, C-10), 38.3 (1C, C-4), 42.0 (1C, C-3a), 46.2 (1C, C-10-a), 52.1 (1C, CO_2_*C*H_3_), 52.3 (1C, NH*C*HCH_2_), 55.8 (2C, 3-OCH_3_, 5-OCH_3_), 59.9 (1C, 4-OCH_3_), 105.8 (2C, C-2_TMP_, C-6_TMP_), 111.2 (1C, C-6), 111.8 (1C, C-9b), 113.7 (1C, C-5_imidaz._), 117.9 (1C, C-9), 118.7 (1C, C-8), 121.5 (1C, C-7), 125.5 (1C, C-9a), 130.4 (1C, C-4a), 135.1 (1C, C-2_imidaz._), 136.1 (1C, C-4_TMP_), 136.1 (1C, C-5a), 137.0 (1C, C-1_TMP_), 152.3 (2C, C-3_TMP_, C-5_TMP_), 170.1 (1C, CO_amide_), 172.1 (1C, *C*O_2_CH_3_), 178.1 (1C, C-1), 180.2 (1C, C-3). A signal for the C-4_imidaz._ C-atom was not observed in the spectrum. Exact mass (ESI): *m/z* = 602.2254 (calcd. 602.2245 for C_31_H_32_N_5_O_8_ [MH]^+^). IR (neat): ṽ [cm^−1^] = 2978, 2886 (s, C-H_aliph_.), 1705, 1674 (s, C=O), 1153, 1125 (s, C-O), 745 (s, C-H_arom._).

**Ethyl (3a*RS*,4*SR*,10*RS*,10a*RS*)-10-(4-nitrophenyl)-1,3-dioxo-1,2,3,3a,4,5,10,10a-octahydropyrrolo[3,4-*b*]carbazole-4-carboxylate (21a)**

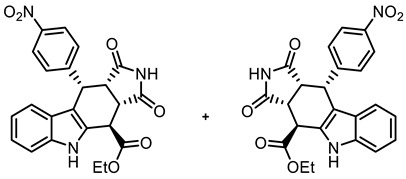

Under N_2_, indolylacetate **6** (205 mg, 1.01 mmol), maleimide (**8a**, 292 mg, 3.00 mmol), and 4-nitrobenzaldehyde (**7b**, 228 mg, 1.51 mmol) were dissolved in dry *o*-xylene (15 mL) in a pressure-resistant Schlenk tube. Crushed CuSO_4_ ∙ 5 H_2_O (26.4 mg, 0.11 mmol) was added to the solution and the mixture was heated to reflux for 40 h (oil bath temperature 180 °C). After cooling to room temperature, the mixture was diluted with CH_2_Cl_2_ until a clear solution formed. After filtration, the filter was washed with CH_2_Cl_2_ (3 × 10 mL). The filtrate was concentrated in vacuo. The residue was again dissolved in CH_2_Cl_2_ (2 mL) and adsorbed onto silica gel, followed by purification by automatic flash column chromatography (cartridge: SNAP 100g, flow rate of 50 mL/min, ethyl acetate/cyclohexane = 10:90 → 0:100). For further purification, the resulting solid was left stirring in Et_2_O (5 mL) for 30 min. The suspension was filtrated, washed with cold Et_2_O (3 × 10 mL), and dried under vacuum. Yellow solid, mp 262 °C, yield of 107 mg (25%). C_23_H_19_N_3_O_6_ (433.4). TLC (ethyl acetate/cyclohexane = 1:1): R_f_ = 0.26. Purity (HPLC): 94.9%, (t_R_ = 20.1 min). ^1^H NMR (600 MHz, DMSO-*d_6_*): δ (ppm) = 1.31 (t, *J* = 7.1 Hz, 3H, COCH_2_C*H_3_*), 3.72 (t, *J* = 9.0 Hz, 1H, 10a-H), 4.09 (dd, *J* = 9.0/3.8 Hz, 1H, 3a-H), 4.22–4.33 (m, 2H, COC*H_2_*CH_3_), 4.56 (d, *J* = 3.7 Hz, 1H, 4-H), 4.99 (d, *J* = 8.9 Hz, 1H, 10-H), 6.83 (ddd, *J* = 7.9/6.8/1.0 Hz, 1H, 8-H), 6.98 (d, *J* = 7.8 Hz, 9H), 7.04 (ddd, *J* = 8.1/6.9/1.1 Hz, 1H, 7H), 7.25–7.30 (m, 2H, 2-H_NO2Ph_, 6-H_NO2Ph_), 7.35–7.39 (dt, *J* = 8.1/0.8 Hz, 1H, 6-H), 8.03–8.07 (m, 2H, 3-H_NO2Ph_, 5-H_NO2Ph_), 10.97 (s, 1H, 2-H), 11.10 (s, 1H, 5-H). ^13^C NMR (151 MHz, DMSO-*d_6_*): δ (ppm) = 14.0 (1C, COCH_2_*C*H_3_), 37.1 (1C, C-4), 37,4 (1C, C-10), 42.6 (1C, C-3a), 45.0 (1C, C-10a), 61.7 (1C, CO*C*H_2_CH), 109.9 (1C, C-9b), 111.6 (1C, C-6), 118.1 (1C, C-9), 118.9 (1C, C-8), 121.7 (1C, C-7), 123.0 (2C, C-3_NO2Ph_, C-3_NO2Ph_), 125.2 (1C, C-9a), 129.2 (1C, C-4a), 130.2 (2C, C-2_NO2Ph_, C-6_NO2Ph_), 136.6 (1C, C-5a), 146.3 (1C, C-4_NO2Ph_), 149.1 (1C, C-1_NO2Ph_), 170.9 (1C, *C*OCH_2_CH_3_), 177.5 (1C, C-1), 178.2 (1C, C-3). Exact mass (ESI): *m/z* = 434.1342 (calcd. 434.1347 for C_23_H_20_N_3_O_6_ [MH]^+^). IR (neat): ṽ [cm^−1^] = 2978, 2893 (w, C-H_aliph_.), 1720 (s, C=O), 1508, 1342 (s, N-O) 1141 (s, C-O), 745 (m, C-H_arom._).

**Ethyl (3a*RS*,4*SR*,10*RS*,10a*RS*)-2-methyl-10-(4-nitrophenyl)-1,3-dioxo-1,2,3,3a,4,5,10,10a-octahydropyrrolo[3,4-*b*]carbazole-4-carboxylate (21b)**

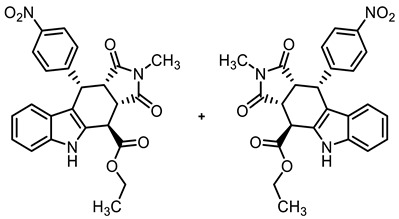

Under N_2_, indolylacetate **6** (101 mg, 0.50 mmol), *N*-methylmaleimide (**8b**, 281 mg, 2.53 mmol), and 4-nitrobenzaldehyde (**7b**, 132 mg, 0.87 mmol) were dissolved in dry *o*-xylene (15 mL) in a pressure-resistant Schlenk tube. Crushed CuSO_4_ ∙ 5 H_2_O (12.4 mg, 0.05 mmol) was added to the solution and the mixture was heated to reflux for 40 h (oil bath temperature of 180 °C). After cooling to room temperature, the mixture was filtered, and the filter was washed with CH_2_Cl_2_ (3 × 10 mL). The filtrate was concentrated in vacuo and the residue was purified by flash column chromatography (ethyl acetate/cyclohexane = 4:7, Ø 4 cm, h = 12 cm, v = 20 mL). Yellow solid, mp 237 °C, yield of 82 mg (40%). C_24_H_21_N_3_O_6_ (447.4). TLC (ethyl acetate/cyclohexane = 4:6): R_f_ = 0.21. Purity (HPLC): 95.3%, (t_R_ = 21.3 min). ^1^H NMR (600 MHz, DMSO-*d_6_*): δ (ppm) = 1.30 (t, *J* = 7.1 Hz, 3H, OCH_2_C*H_3_*), 2.36 (s, 3H, NHC*H_3_*), 3.80 (t, *J* = 8.8 Hz, 1H, 10a-H), 4.10 (dd, *J* = 8.6/3.4 Hz, 1H, 3a-H), 4.16–4.36 (m, 2H, OC*H_2_*CH_3_), 4.62 (d, *J* = 3.4 Hz, 1H, 4-H), 5.01 (d, *J* = 8.9 Hz, 1H, 10-H), 6.82 (ddd, *J* = 8.0/7.0/1.0 Hz, 1H, 8-H), 6.93 (d, *J* = 8.2 Hz, 1H, 9-H), 7.03 (ddd, *J* = 8.2/7.0/1.3 Hz, 1H, 7-H), 7.17–7.25 (m, 2H, 2-H_NO2Ph_ 6-H_NO2Ph_), 7.37 (dt, *J* = 8.1/0.9 Hz, 1H, 6-H), 7.98–8.10 (m, 3-H_NO2Ph_, 5-H_NO2Ph_), 11.14 (s, 1H, 5-H). ^13^C NMR (151 MHz, DMSO-*d_6_*): δ (ppm) = 14.0 (1C, OCH_2_*C*H_3_), 23.8 (1C, NHC*H_3_*), 37.0 (1C, C-4), 37.6 (1C, C-10), 41.6 (1C, C-3a), 44.0 (1C C-10a), 61.7 (1C, O*C*H_2_CH_3_), 109.6 (1C, C-9b), 111.5 (1C, C-6), 118.2 (1C, C-9), 118.9 (1C, C-8), 121.7 (1C, C-7), 122.9 (2C, C-3_NO2Ph_, C-5_NO2Ph_) 125.1 (1C, C-9a), 129.1 (1C, C-4a), 130.0 (2C, C-2_NO2Ph_, C-6_NO2Ph_), 136.6 (1C, C-5a), 146.3 (1C, C-4_NO2Ph_), 148.8 (1C, C-1_NO2Ph_), 170.8 (1C, CO_ester_) 176.2 (1C, C-1), 177.4 (1C, C-3). Exact mass (ESI): *m/z* = 446.1506 (calcd. 448.1503 for C_24_H_22_N_3_O_6_ [MH]^+^). IR (neat): ṽ [cm^−1^] = 2978 (w, C-H_aliph_.), 1782, (w, C=O), 1717 (s, C=O), 1196,1150 (s, C-O), 745 (m, C-H_arom._).

**Ethyl (3a*RS*,4*SR*,10*RS*,10a*RS*)-2-benzyl-10-(4-nitrophenyl)-1,3-dioxo-1,2,3,3a,4,5,10,10a-octahydropyrrolo[3,4-*b*]carbazole-4-carboxylate (21c)**

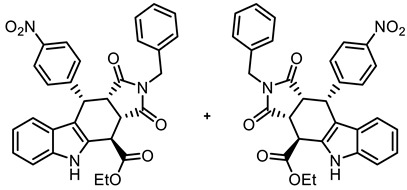

Under N_2_, indolylacetate **6** (104 mg, 0.52 mmol), *N*-benzylmaleimide (**8c**, 282 mg, 1.50 mmol), and 4-nitrobenzaldehyde (**7b**, 113 mg, 0.75 mmol) were dissolved in dry *o*-xylene (15 mL) in a pressure-resistant Schlenk tube. Crushed CuSO_4_ ∙ 5 H_2_O (12.9 mg, 0.05 mmol) was added to the solution and the mixture was heated to reflux for 24 h (oil bath temperature of 180 °C). The mixture was allowed to cool down to ambient temperature and CH_2_Cl_2_ (20 mL) was added until a clear solution formed. After filtration, the filter was washed with CH_2_Cl_2_ (3 × 10 mL). The filtrate was concentrated in vacuo. The residue was purified by flash column chromatography (ethyl acetate/CH_2_Cl_2_ = 2:98, Ø 3 cm, h = 22 cm, v = 20 mL). Yellow solid, mp 199 °C, yield of 109 mg (40%). C_30_H_25_N_3_O_6_ (523.5). TLC (ethyl acetate/CH_2_Cl_2_ = 2:98): R_f_ = 0.33. Purity (HPLC): 98.7%, (t_R_ = 23.2 min). ^1^H NMR (600 MHz, DMSO-*d_6_*): δ (ppm) 1.33 (t, *J* = 7.1 Hz, 3H, OCH_2_C*H_3_*), 3.86 (t, *J* = 9.0 Hz, 1H, 10a-H), 4.15 (d, *J* = 14.8 Hz, 1H, NC*H_2_*Ph), 4.20 (d, *J* = 14.8 Hz, 1H, NC*H_2_*Ph), 4.24 (dd, *J* = 9.3/4.6 Hz, 1H, 3a-H), 4.26–4.40 (m, 2H, OC*H_2_*CH_3_), 4.62 (d, *J* = 4.5 Hz, 1H, 4-H), 5.04 (d, *J* = 8.7 Hz, 1H, 10-H), 6.83 (ddd, *J* = 8.1/7.1/1.4 Hz, 1H, 8-H), 6.88–6.95 (m, 2H, C-2_benzyl_, C-6_benzyl_), 7.02–7.08 (m, 5H, 7-H, 9-H, 3-H_benzyl_, 4-H_benzyl_, 5-H_benzyl_), 7.10–7.13 (m, 2H, 2-H_NO2Ph_, 6-H_NO2Ph_), 7.35–7.40 (m, 1H, 6-H), 7.75–7.79 (m, 2H, 3-H_NO2Ph_, 5-H_NO2Ph_), 11.07 (s, 1H, 5-H). ^13^C NMR (151 MHz, DMSO-*d_6_*): δ (ppm) = 14.0 (1C, OCH_2_*C*H_3_), 37.3 (1C, C-10), 37.6 (1C, C-4), 41.5 (1C, N*C*H_2_Ph), 41.6 (1C, C-3a), 43.8 (1C, C-10a), 61.9 (1C, O*C*H_2_CH_3_), 110.3 (1C, C-9b), 111.6 (1C, C-6), 118.0 (1C, C-9), 118.9 (1C, C-8), 121.7 (1C, C-7), 123.0 (2C, C-3_NO2Ph_, C-5_NO2Ph_), 125.1 (1C, C-9a), 127.1 (1C, C-4_benzyl_), 127.9 (2C, C-3_benzyl_, C-5_benzyl_), 128.0 (2C, C-2_benzyl_, C-6_benzyl_), 129.1 (1C, C-4a), 129.8 (2C, C-2_NO2Ph_, C-6_NO2Ph_), 135.3 (1C C-1_benzyl_), 136.5 (1C, C-5a), 146.1 (1C, C-4_NO2Ph_), 148.2 (1C, C-1_NO2Ph_), 170.9 (1C, C-4-*C*=O), 175.9 (1C, C-1), 177.7 (1C, C-3). Exact mass (ESI): *m/z* = 524.1794 (calcd. 524.1816 for C_30_H_26_N_3_O_6_ [MH]^+^). IR (neat): ṽ [cm^−1^] = 2978, 2928 (w, C-H_aliph_.), 1732, 1701 (s, C=O), 1342 (s, N-O) 1141 (s, C-O_ester_), 745, 689 (m, C-H_arom._).

**Ethyl (3a*RS*,4*SR*,10*RS*,10a*R*S)-10-(4-aminophenyl)-1,3-dioxo-1,2,3,3a,4,5,10,10a-octahydropyrrolo[3,4-*b*]carbazole-4-carboxylate∙HCl (22a.HCl)**

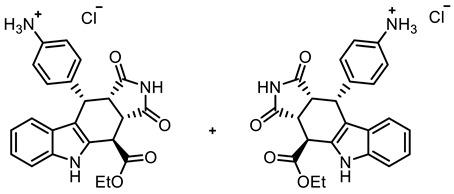

Aromatic nitro compound **21a** (119 mg, 0.28 mmol) was dissolved in THF (20 mL), and saturated NH_4_Cl solution (20 mL) was added. Under vigorous stirring, a large excess of Zn dust (1.01 g, 15.4 mmol) was poured into the mixture, which was then stirred for 2 h at room temperature. After the completion of the transformation, saturated NaHCO_3_ solution (30 mL) was added, and the suspension was stirred for an additional 30 min at room temperature. The reaction mixture was filtered through Celite^®^, followed by rinsing with ethyl acetate (150 mL). The filtrate was poured into a separating funnel and the layers were separated. The aqueous layer was then extracted with ethyl acetate (2 × 50 mL). The combined organic layers were dried (Na_2_SO_4_), filtered, and concentrated in vacuo. The residue was purified by flash column chromatography (ethyl acetate/cyclohexane = 8:2, Ø 2 cm, h = 12 cm, v = 10 mL). For conversion into the HCl salt, the resulting solid (74 mg) was dissolved in CH_2_Cl_2_ (5 mL). HCl solution (1 M in Et_2_O, 3 mL) was added, and the suspension was concentrated in vacuo. Orange solid, mp 244 °C, yield of 75 mg (62%). C_23_H_22_ClN_3_O_4_ (439.9). TLC (ethyl acetate/cyclohexane = 8:2): R_f_ = 0.21 (free base). Purity (HPLC): 95.9%, (t_R_ = 15.1 min). ^1^H NMR (600 MHz, DMSO-*d_6_*): δ (ppm) = 1.31 (t, *J* = 7.1 Hz, 3H, COCH_2_C*H_3_*), 3.62 (t, *J* = 8.9 Hz, 1H, 10a-H), 4.06 (dd, *J* = 9.1/3.9 Hz, 1H, 3a-H), 4.21–4.35 (m, 2H, COC*H_2_*CH_3_), 4.51 (d, *J* = 3.9 Hz, 1H, 4-H), 4.82 (d, *J* = 8.7 Hz, 1H, 10-H), 6.82 (ddd, *J* = 7.9/6.9/1.0 Hz, 1H, 8-H), 6.99 (d, *J* = 7.9 Hz, 1H, 9-H), 7.01–7.07 (m, 3H, 7-H, 3-H_NH2Ph_, 5-H_NH2Ph_), 7.10 (d, *J* = 8.1 Hz, 2H, 2-H_NH2Ph_, 6-H_NH2Ph_), 7.32–7.40 (m, 1H, 6-H), 9.79 (s, 3H, N*H_3_*^+^), 10.95 (s, 1H, 2-H), 11.01 (s, 1H, 5-H). ^13^C NMR (151 MHz, DMSO-*d_6_*): δ (ppm) = 14.0 (1C, COCH_2_*C*H_3_), 37.1 (1C, C-4), 37.2 (1C, C-10), 42.6 (1C, C-3a), 45.2 (1C, C-10a), 61.7 (1C, CO*C*H_2_CH), 110.9 (1C, C-9b), 111.5 (1C, C-6), 118.1 (1C, C-9), 118.7 (1C, C-8), 121.6 (1C, C-7), 121.8 (2C, C-2_NH2Ph_, C-6_NH2Ph_), 125.2 (1C, C-9a), 129.0 (1C, C-4a), 130.0 (2C, C-3_NH2Ph_, C-5_NH2Ph_), 136.5 (1C, C-5a), 171.0 (1C, *C*OCH_2_CH_3_), 177.6 (1C, C-1), 179.1 (1C, C-3). Signals for C-1_NH2Ph_ and C-4_NH2Ph_ C-atoms were not observed in the spectrum. Exact mass (ESI): *m/z* = 404.1606 (calcd. 404.1605 for C_23_H_22_N_3_O_4_ [MH]^+^). IR (neat): ṽ [cm^−1^] = 2978 (w, N-H_amine salt_), 2983, 2862 (w, C-H_aliph_.), 1782 (w, C=O), 1701 (s, C=O), 1180 (s, C-O), 1153 (m, C-O), 741 (m, C-H_arom._).

**Ethyl (3a*RS*,4*SR*,10*RS*,10a*RS*)-10-(4-aminophenyl)-2-benzyl-1,3-dioxo-1,2,3,3a,4,5,10,10a-octahydropyrrolo[3,4-*b*]carbazole-4-carboxylate∙HCl (22c.HCl)**

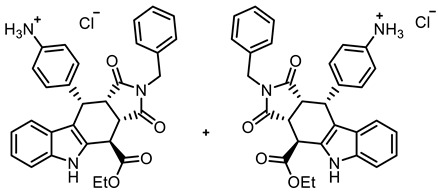

Aromatic nitro compound **21c** (263 mg, 0.50 mmol) was dissolved in THF (30 mL) and saturated NH_4_Cl solution (30 mL) was added. Under vigorous stirring, a large excess of Zn dust (5.00 g, 76.5 mmol) was poured into the mixture, which was then stirred for 2 h at room temperature. After the completion of the transformation, saturated NaHCO_3_ solution (30 mL) was added, and the suspension was stirred for an additional 30 min at room temperature. The reaction mixture was filtered through Celite^®^, followed by rinsing with ethyl acetate (80 mL). The filtrate was poured into a separating funnel and the layers were separated. The aqueous layer was then extracted with ethyl acetate (3 × 30 mL). The combined organic layers were dried (Na_2_SO_4_), filtered, and concentrated in vacuo. For conversion into the HCl salt, the resulting solid was dissolved in CH_2_Cl_2_ (5 mL). HCl solution (1 M in Et_2_O, 3 mL) was added, and the suspension was concentrated in vacuo. Orange solid, mp 206 °C, yield of 126 mg (48%). C_30_H_28_ClN_3_O_4_ (530.0). TLC (ethyl acetate/cyclohexane = 7:3): R_f_ = 0.38 (free base). Purity (HPLC): 95.9%, (t_R_ = 18.7 min). ^1^H NMR (400 MHz, DMSO-*d_6_*): δ (ppm) = 1.30 (t, *J* = 7.1 Hz, 3H, COCH_2_C*H_3_*), 3.81 (t, *J* = 8.8 Hz, 1H, 10a-H), 3.98 (d, *J* = 15.1 Hz, 1H, NC*H_2_*Ph), 4.12 (d, *J* = 15.1 Hz, 1H, NC*H_2_*Ph), 4.20 (dd, *J* = 8.9/4.0 Hz, 1H, 3a-H), 4.22–4.34 (m, 2H, CO*CH_2_*CH_3_), 4.57 (d, *J* = 4.0 Hz, 1H, 4-H), 4.88 (d, *J* = 8.5 Hz, 1H, 10-H), 6.82 (ddd, *J* = 8.0/7.0/1.0 Hz, 1H, 8-H), 6.93–7.09 (m, 8H, 7-H, 9-H, 2-H_NH2Ph_, 3-H_NH2Ph_, 5-H_NH2Ph_, 6-H_NH2Ph_, 3-H_benzyl_, 5-H_benzyl_), 7.18–7.23 (m, 3H, 2-H_benzyl_, 4-H_benzyl_, 6-H_benzyl_), 7.38 (dt, *J* = 8.1/1.1/0.9 Hz, 1H, 6-H), 9.77 (s, 3H, N*H_3_*^+^), 11.08 (s, 1H, 5-H). ^13^C NMR (101 MHz, DMSO-*d_6_*): δ (ppm) = 14.1 (1C, COCH_2_*C*H_3_), 37.51 (1C, C-4), 37.53 (1C, C-10), 41.1 (1C, N*C*H_2_Ph), 41.8 (1C, C-3a), 44.4 (1C, C-10a), 61.8 (1C, CO*C*H_2_CH), 110.7 (1C, C-9b), 111.6 (1C, C-6, 118.2 (1C, C-9), 118.8 (1C, C-8), 121.6 (1C, C-7), 122.1 (2C, C-2_NH2Ph_, C-6_NH2Ph_), 125.2 (1C, C-9a), 127.2 (2C, C-3_benzyl_, C-5_benzyl_), 127.3 (1C, C-4_benzyl_), 128.3 (2C, C-2_benzyl_, C-6_benzyl_), 129.1 (1C, C-4a), 130.0 (2C, C-3_NH2Ph_, C-5_NH2Ph_), 135.5 (1C, C-1_benzyl_), 136.5 (1C, C-5a), 170.8 (1C, *C*OCH_2_CH_3_), 176.2 (1C, C-1), 177.6 (1C, C-3). Signals for C-1_NH2Ph_ and C-4_NH2Ph_ C-atoms are not observed in the spectrum. Exact mass (APCI): *m/z* = 494.2064 (calcd. 494.2074 for C_30_H_28_N_3_O_4_ [MH]^+^). IR (neat): ṽ [cm^−1^] = 2978 (w, N-H_amine salt_), 2978, 2808 (w, C-H_aliph_.), 1697 (s, C=O), 1184, 1146 (m, C-O), 745 (m, C-H_arom._).

**Ethyl (3a*RS*,4*SR*,10*RS*,10a*RS*)-2-benzyl-10-[4-(4-methoxybenzamido)phenyl]-1,3-dioxo-1,2,3,3a,4,5,10,10a-octahydropyrrolo[3,4-*b*]carbazole-4-carboxylate (23)**

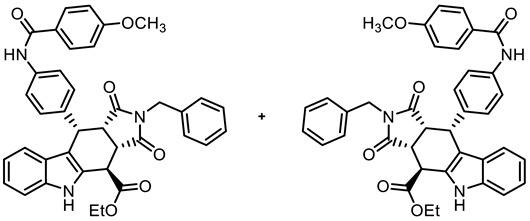

Ammonium chloride **22c∙HCl** (77.5 mg, 0.14 mmol) was suspended in dry CH_2_Cl_2_ (20 mL) followed by the addition of DIPEA (80 µL, 0.47 mmol) to obtain the free base. 4-methoxybenzoyl chloride (43.1 mg, 0.28 mmol) was added and the mixture was stirred for 2 h at room temperature. After the completion of the transformation, the solution was washed with HCl solution (0.1 M, 2 × 10 mL) and saturated NaCl solution (1x 10 mL). The organic layer was dried (Na_2_SO_4_), filtered, and concentrated in vacuo. The residue was purified by flash column chromatography (THF/CH_2_Cl_2_ = 3:97, Ø 3 cm, h = 14 cm, v = 10 mL). Yellow solid, mp 249 °C, yield of 76 mg (82%). C_38_H_33_N_3_O_6_ (627.7). TLC (THF/CH_2_Cl_2_ = 3:97): R_f_ = 0.19. Purity (HPLC): 94.5%, (t_R_ = 22.8 min). ^1^H NMR (600 MHz, DMSO-*d_6_*): δ (ppm) = 1.32 (t, *J* = 7.1 Hz, 3H, COCH_2_C*H_3_*), 3.78 (t, *J* = 8.9 Hz, 1H, 10a-H), 3.83 (s, 3H, OCH_3_), 4.04 (d, *J* = 15.2 Hz, 1H, NC*H_2_*Ph), 4.19 (dd, *J* = 9.2/4.3 Hz, 1H, 3a-H), 4.20 (d, *J* = 15.1 Hz, 1H, NC*H_2_*Ph), 4.24–4.37 (m, 2H, COC*H_2_*CH_3_), 4.57 (d, *J* = 4.3 Hz, 1H, 4-H), 4.83 (d, *J* = 8.4 Hz, 1H, 10-H), 6.83–6.88 (m, 3H, 8-H, 2-H_NH2Ph_, 6-H_NH2Ph_), 6.89–6.93 (m, 2H, 2-H_benzyl_, 6-H_benzyl_), 7.01–7.09 (m, 4H, 7-H, 9-H, 3-H_MeObz_, 5-H_MeObz_), 7.09–7.18 (m, 3H, 3-H_benzyl_, 4-H_benzyl_, 5-H_benzyl_), 7.38 (dt, *J* = 8.3/0.9 Hz, 1H, 6-H), 7.48–7.54 (m, 2H, 3-H_NH2Ph_, 5-H_NH2Ph_), 7.90–7.96 (m, 2H, 2-H_MeObz_, 6-H_MeObz_), 9.98 (s, 1H, NH_amide_), 11.00 (s, 1H, 5-H). ^13^C NMR: (151 MHz, DMSO-*d_6_*) δ (ppm) = 14.0 (1C, COCH_2_*C*H_3_), 37.5 (2 × 1C, C-4_,_ C-10), 41.2 (1C, N*C*H_2_Ph), 41.8 (1C, C-3a), 44.7 (1C, C-10a), 55.4 (1C, OCH_3_), 61.7 (1C, CO*C*H_2_CH), 111.3 (1C, C-9b), 111.5 (1C, C-6), 113.6 (2C, C-3_MeObz_, C-5_MeObz_), 118.2 (1C, C-9), 118.8 (1C, C-8), 119.5 (2C, C-3_NH2Ph_, C-5_NH2Ph_), 121.5 (1C, C-7), 125.3 (1C, C-9a), 127.0 (1C, C-4_benzyl_), 127.0 (1C, C-1_MeObz_), 127.2 (2C, C-2_benzyl_, C-6_benzyl_) 128.1 (2C, C-3_benzyl_, C-5_benzyl_), 128.8 (2C, C-2_NH2Ph_, C-6_NH2Ph_), 128.9 (1C, C-4a), 129.5 (2C, C-2 _MeObz_, C-6 _MeObz_), 134.9 (1C, C-1_NH2Ph_), 135.5 (1C, C-1_benzyl_), 136.5 (1C, C-5a), 138.1 (1C, C-4_NH2Ph_), 161.8 (1C, C-4 _MeObz_), 164.7 (1C, *C*O_amide_), 170.9 (1C, *C*OCH_2_CH_3_), 176.2 (1C, C-1), 177.8 (1C, C-3). Exact mass (APCI): *m/z* = 628.2400 (calcd. 628.2442 for C_38_H_34_N_3_O_6_ [MH]^+^). IR (neat): ṽ [cm^−1^] = 2978, 2889 (m, C-H_aliph_.), 1775 (w, C=O), 1694 (s, C=O), 1643, 1605 (m, C=C_aryl_) 1184 (s, C-O), 1153 (m, C-O).

### 4.4. Microscale Thermophoresis Assay

The recombinant expression and purification [37,47] of human CK2α^1–335^ and human CK2β^1−193^, as well as the microscale thermophoresis (MST) assay to quantify the interaction of the two CK2 subunits, were performed as described previously [40,45]. Briefly, MST experiments followed the interaction between 20 nM of fluorescently labelled CK2β^1–193^ and 16 serial dilutions of CK2α^1–335^ (0.1526–5000 nM) in 25 mM Tris-HCl, 500 mM NaCl, pH 8.5, 1% (*v*/*v*) DMSO, and 0.05% (*v*/*v*) Tween 20. The compounds were investigated at a final concentration of 20, 50, or 100 µM. These experiments resulted in dissociation constants of *K*_D_ and *K*_D_′ for the uninhibited and inhibited CK2 subunit interactions, respectively, which were then subjected to an unpaired Student’s t test to analyze for the statistical significance of the difference in the values. Data in the absence of inhibitors and in the presence of those compounds leading to a statistically significant increase in the dissociation constant (*p* ≤ 0.05) were analyzed again by setting *K*_D_ to a global value and, in addition, using the equation *K*_D_′ = *K*_D_ [1 + (c_compound_/*K*_i_)]. The global analysis gave a *K*_D_ of 11 nM, which was in agreement with reported values [40,45] and provided the *K*_i_ values listed in Table 1. Data analysis and statistical evaluation were conducted with GraphPad Prism v.6 for Windows (GraphPad Software, La Jolla, CA, USA, www.graphpad.com).

### 4.5. Capillary Electrophoresis Assay

The capillary electrophoresis assay to determine the inhibition of the CK2 holoenzyme (CK2α_2_β_2_) was performed as described in refs. [40,46]. The CK2 holoenzyme was recombinantly expressed in *E. coli* BL21(DE3) and purified according to the protocol by Grankowski et al. [48]. Enzymatic activity was determined in the presence of 1 µg of CK2α_2_β_2_, 60 µM of ATP, and 114 µM of the substrate peptide RRRDDDSDDD. The assay buffer contained 60 mM NaCl and 10 mM MgCl_2_. Inhibition was determined in three independent experiments at a compound concentration of 10 µM, and the mean value and the standard deviation (SD) were calculated. When the holoenzyme inhibition exceeded 50% in comparison to the enzymatic activity without inhibitors, obtained with the same amount of DMSO, an IC_50_ value was determined three times independently.

## Figures and Tables

**Table 1:** Inhibition of the CK2α/CK2β association by the tetracyclic pyrrolocarbazoles **9**–**23** determined in the MST assay compared with the activity of the lead compound W16 (**4**).

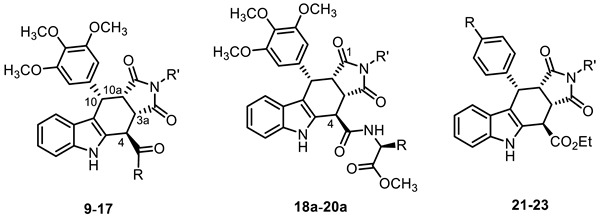					
Compd.	R	R′	Conc. Test Compd. (µM)	CK2α/CK2β Interaction *K*_D_′ [nM] ^a^	Inhibition of CK2α/CK2β Interaction *K*_i_ [µM]
(±)-**9a** [40]	OEt	H	100	59 ± 25	32 ^d^
(±)-**9b**	OEt	CH_3_	20	14 ± 6 ^b^	n.s.
(±)-**10** [40]	OH	H	100	758 ± 338	1.9 ^d^
(±)-**11**	NHCH_3_	H	50	132 ± 43	6.0
(±)-**12**	NHCH_2_C_6_H_5_	H	50	62 ± 19	14
(±)-**13**	NHCH_2_-4-ClC_6_H_4_	H	50	17 ± 3 ^c^	n.s.
(±)-**14**	NH(CH_2_)_2_-indol-3-yl	H	50	158 ± 17	3.8
(±)-**15**	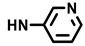	H	50	89 ± 13	7.4
(±)-**16**	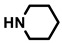	H	50	72 ± 12	9.7
(±)-**17**	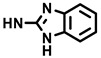	H	50	93 ± 13	7.1
(+)-**18a**	CH_2_OH	H	50	72 ± 2	9.1
(–)-**19b**	CH_2_CH_2_SCH_3_ (S-RSRR-config.)	H	50	94 ± 24	7.7
(+)-**20a**	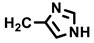	H	50	101 ± 4	6.1
(–)-**20b**	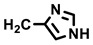 (S-RSRR-config.)	H	20	29 ± 7 ^b^	n.s.
(±)-**21a**	NO_2_	H	100	20 ± 10 ^c^	n.s.
(±)-**22a**	NH_2_	H	100	31 ± 11 ^c^	n.s.
(±)-**22c**	NH_2_	Bn	100	25 ± 15 ^c^	n.s.
(±)-**23**	NHC(=O)aryl	Bn	100	24 ± 9 ^c^	n.s.
(+)-**4** (W16) [40]			100	61 ± 17	31 ^d^

^a^ Mean ± SEM values of 2–4 separate experiments resulting from a global analysis of all included data sets in Table 1 and in ref. [40] where *K*_D_′ values were significantly different (unpaired Student’s *t*-test, *p* ≤ 0.05) from the *K*_D_ value of 12 ± 1 nM (n = 4) for the uninhibited CK2α/CK2β interaction. A global value of *K*_D_ = 11 ± 7 nM (n = 4) was calculated in this global analysis. ^b,c^ *K*_D_′ values were not included in the global analysis. An unpaired Student’s *t*-test showed each *K*_D_′ value to be non-significantly different (*p* > 0.05) from a *K*_D_ value of 20 ± 9 nM (n = 3) ^b^ or 12 ± 1 nM (n = 4) ^c^ for the uninhibited CK2α/CK2β interaction. ^d^ *K*_i_ values in ref. [40] were 32 µM ((±)-**9a**), 1.8 µM ((±)-**10**), and *3*1 µM ((+)-**4**). n.s.: not significant.

## Data Availability

All data are included in the manuscript and the corresponding Supporting Information.

## Supplementary Materials

The following supporting information can be downloaded at: https://www.mdpi.com/article/10.3390/molecules30010063/s1, Supporting Information contain the formula to calculate the *K*_i_ values, ^1^H and ^13^C NMR spectra and HPLC chromatograms of the prepared compounds.

## Abbreviations

ATP: adenosine triphosphate; CE: capillary electrophoresis; CNS: central nervous system; GTP: guanosine triphosphate; MST: microscale thermophoresis; OCNDS: Okur–Chung neurodevelopmental syndrome; PPI: protein–protein interactions; PPII: protein–protein interaction inhibition; POBINDS: Poirier–Bienvenu neurodevelopmental syndrome; SD: standard deviation; SEM: standard error of the mean.
