# Supplementary material for: Structure–Activity Relationship Studies of Tetracyclic Pyrrolocarbazoles Inhibiting Heterotetrameric Protein Kinase CK2
Source: Molecules. 2024 Dec 27;30(1):63. doi: 10.3390/molecules30010063 (PMC11722180; doi:10.3390/molecules30010063)
Supplement: Supplementary file 1 [file molecules-30-00063-s001.zip › molecules-3358620-supplementary.pdf]

Supporting information

Structure activity relationship studies of tetracyclic pyrrolocarbazoles inhibiting the heterotetrameric proteinkinase CK2

Lukas Kröger,[a] Sebastian Borgert,[a] Miriam Lauwers,[b] Michaela Steinkrüger,[b] Joachim Jose,[a] Markus Pietsch,[b,c] Bernhard Wünsch[a]*

[a] Universität Münster, Institut für Pharmazeutische und Medizinische Chemie, Corrensstraße 48, D-48149 Münster, Germany, Tel.: +49-251-8333311; Fax: +49-251-8332144; Email: wuensch@uni-muenster.de

[b] Institutes I & II of Pharmacology, Center of Pharmacology, Faculty of Medicine and University Hospital Cologne, University of Cologne, D-50931 Cologne, Germany.

[c] Faculty of Applied Natural Sciences, TH Köln-University of Applied Sciences, Campus Leverkusen, D-51379 Leverkusen, Germany.

Content page

1. Chemistry, general methods S2
2. HPLC method to determine the purity of the compounds S3
3. Formula to calculate the K_i value S3
4. ^{1}H and ^{13}C NMR spectra S4
5. Purity by HPLC analysis S25

1. Chemistry, general methods

Oxygen and moisture sensitive reactions were carried out under nitrogen dried with silica gel with moisture indicator (orange gel, VWR, Darmstadt, Germany) and in dry glassware (Schlenk flask or Schlenk tube). Temperature was controlled with dry ice/acetone (-78 °C), ice/water (0 °C), Cryostat (Julabo TC100E-F, Seelbach, Germany), magnetic stirrer MR 3001 K (Heidolph, Schwalbach, Germany) or RCT CL (IKA, Staufen, Germany), together with temperature controller EKT HeiCon (Heidolph) or VT-5 (VWR) and PEG or silicone bath. All solvents were of analytical or technical grade quality. *o*-Xylene and toluene were dried with molecular sieves (3Å). Demineralized water was used. Thin layer chromatography (tlc): tlc silica gel 60 F_{254} on aluminum sheets (VWR). Flash chromatography (fc): Silica gel 60, 40–63 µm (VWR); parentheses include: diameter of the column (Ø), length of the stationary phase (l), fraction size (v) and eluent. Automated flash chromatography: Isolera™ Spektra One (Biotage®); parentheses include: cartridge size, flow rate, eluent, fractions size was always 20 mL. Melting point: Melting point system MP50 (Mettler Toledo, Gießen, Germany), open capillary, uncorrected. MS: MicroTOFQII mass spectrometer (Bruker Daltonics, Bremen, Germany); deviations of the found exact masses from the calculated exact masses were 5 mDa or less; the data were analyzed with DataAnalysis® (Bruker Daltonics). NMR: NMR spectra were recorded in deuterated solvents on Agilent DD2 400 MHz and 600 MHz spectrometers (Agilent, Santa Clara CA, USA); chemical shifts (*δ*) are reported in parts per million (ppm) against the reference substance tetramethylsilane and calculated using the solvent residual peak of the undeuterated solvent; coupling constants are given with 0.5 Hz resolution; assignment of ^{1}H and ^{13}C NMR signals was supported by 2-D NMR techniques where necessary. IR: FT/IR Affinity®-1 spectrometer (Shimadzu, Düsseldorf, Germany) using ATR technique.

2. HPLC method to determine the purity of the compounds

Equipment 1: Pump: L-7100, degasser: L-7614, autosampler: L-7200, UV detector: L-7400, interface: D-7000, data transfer: D-line, data acquisition: HSM-Software (all from Merck Hitachi, Darmstadt, Germany); Equipment 2: Pump: LPG-3400SD, degasser: DG-1210, autosampler: ACC-3000T, UV-detector: VWD-3400RS, interface: DIONEX UltiMate 3000, data acquisition: Chromeleon 7 (equipment and software from Thermo Fisher Scientific, Lauenstadt, Germany); column: LiChrospher® 60 RP-select B (5 µm), LiChroCART® 250-4 mm cartridge; flow rate: 1.0 mL/min; injection volume: 5.0 µL; detection at λ = 210 nm; solvents: A: demineralized water with 0.05 % (V/V) trifluoroacetic acid, B: CH_3CN with 0.05 % (V/V) trifluoroacetic acid; gradient elution (% A): 0 - 4 min: 90 %; 4 - 29 min: gradient from 90 % to 0 %; 29 - 31 min: 0 %; 31 - 31.5min: gradient from 0 % to 90 %; 31.5 - 40 min: 90 %.

3. Formula to calculate the K_i value

The recorded K_D value for the inhibition of the K2α7CK2β subunit interaction was transformed into the K_i value using the following formula:

$$\mathrm{K_i} = \frac{[\mathrm{Inhibitor}]}{\left(\left(\frac{\mathrm{K_D}(\text{with Inhibitor})}{\mathrm{K_D}\ (\text{without Inhibitor})}\right) - 1\right)}$$

[Inhibitor]: concentration of the inhibitor in M.

K_D (with inhibitor): Recorded K_D value in the presence of given concentration of inhibitor.

K_D (without inhibitor): K_D value recorded in the absence of any inhibitor.

4. ^{1}H and ^{13}C NMR spectra

^{1}H NMR spectrum of **9a** in $CDCl_3$

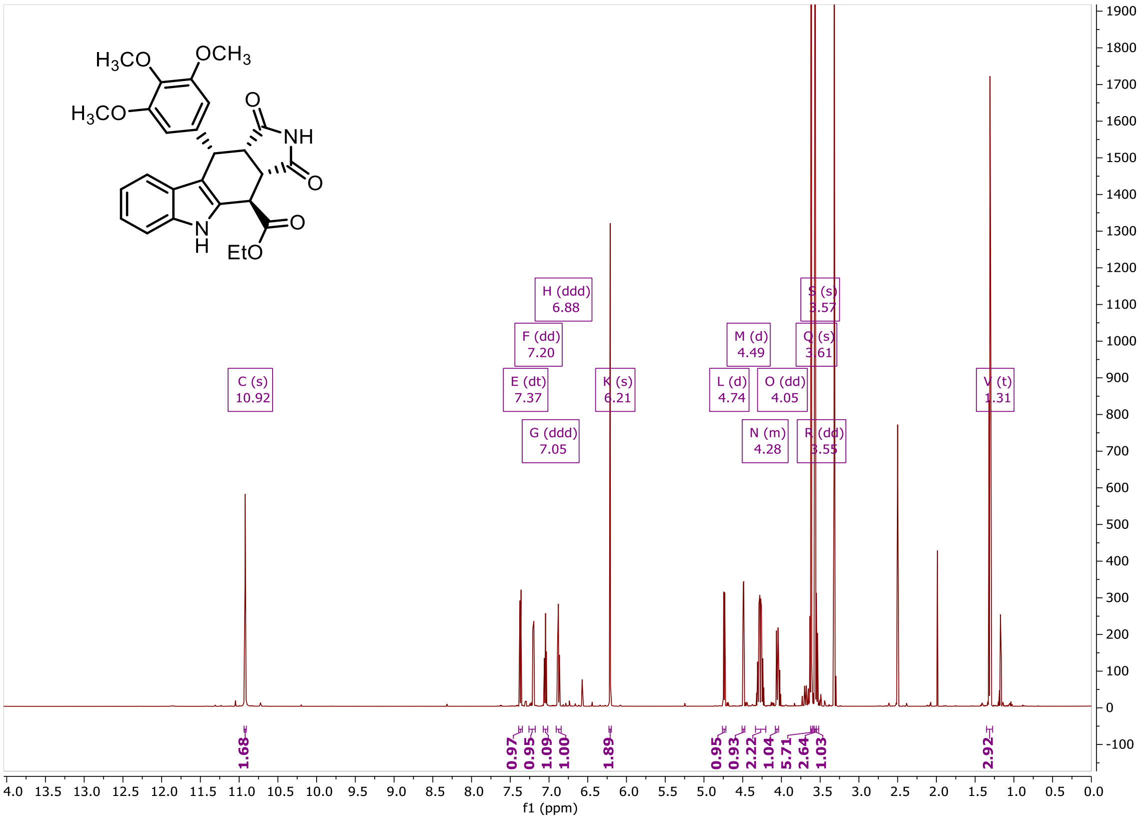

^{13}C NMR spectrum of (±)-**9a** in DMSO-d_6.

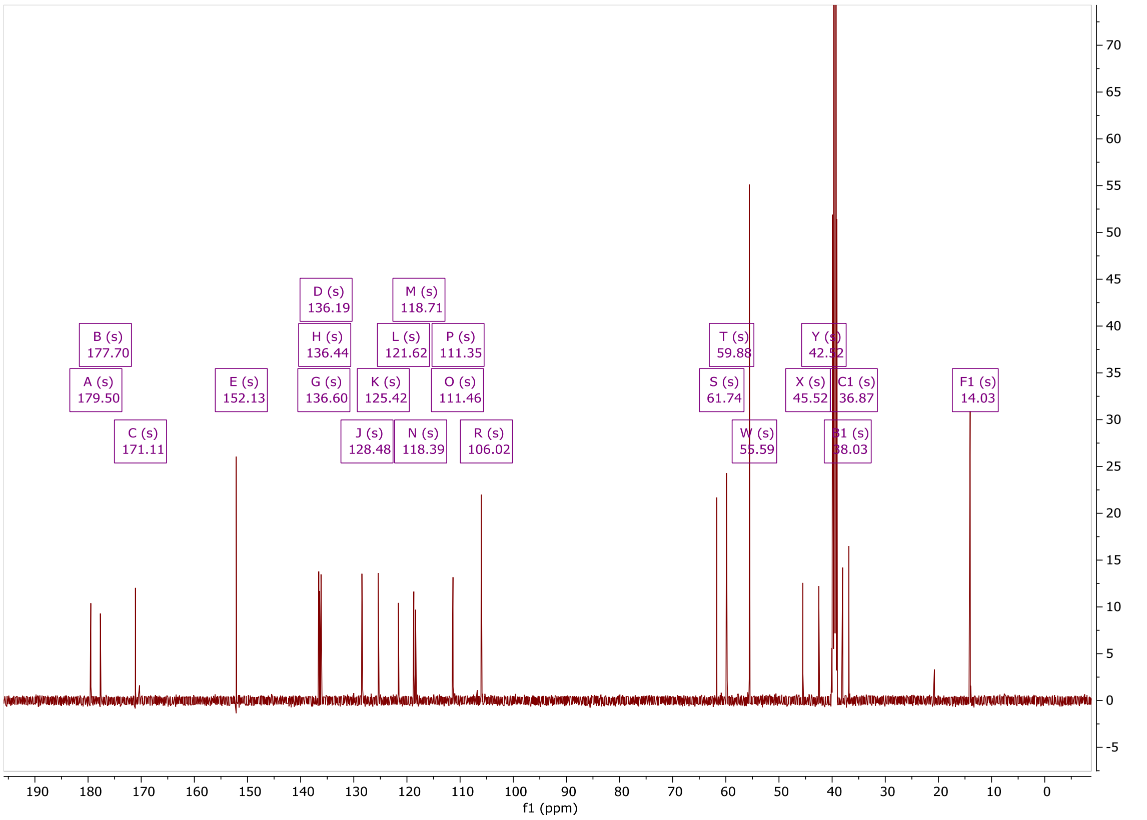

^{1}H NMR spectrum of **9b** in $CDCl_3$

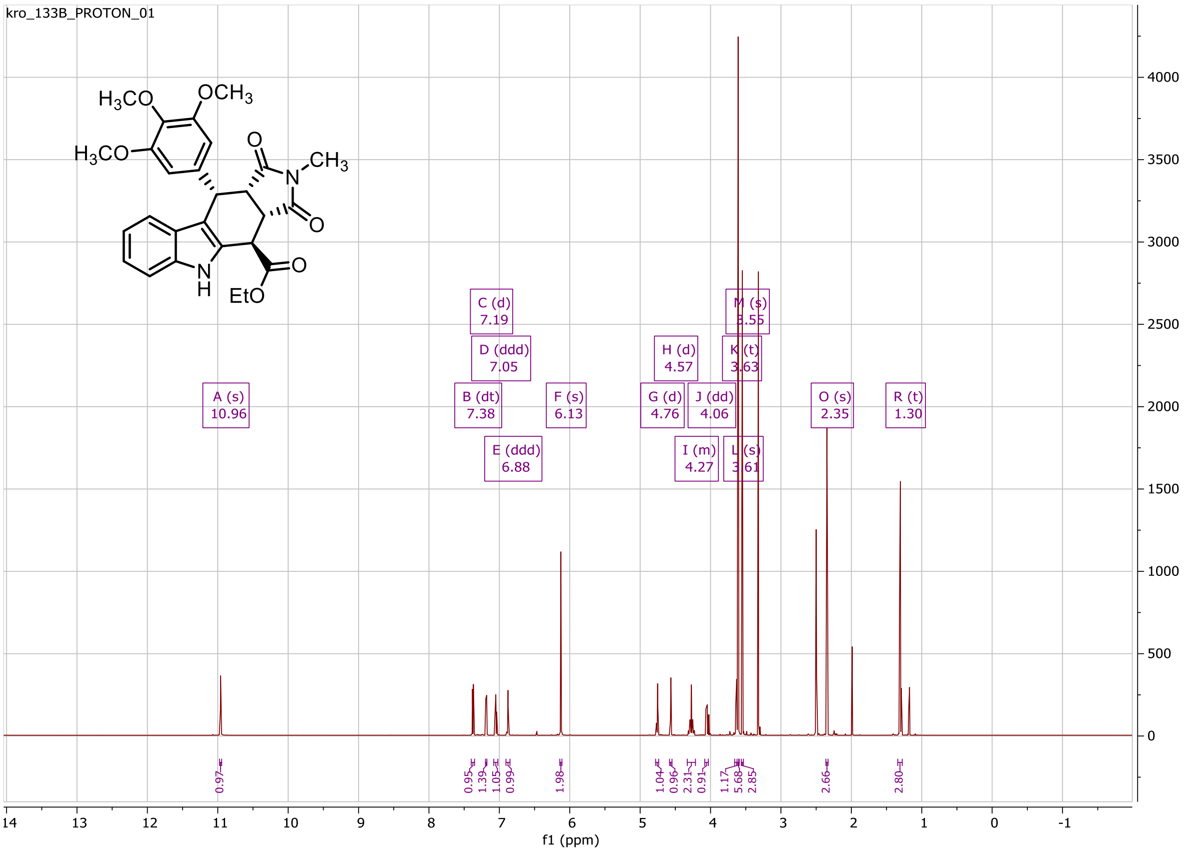

^{13}C NMR spectrum of (±)-**9b** in DMSO-d_6.

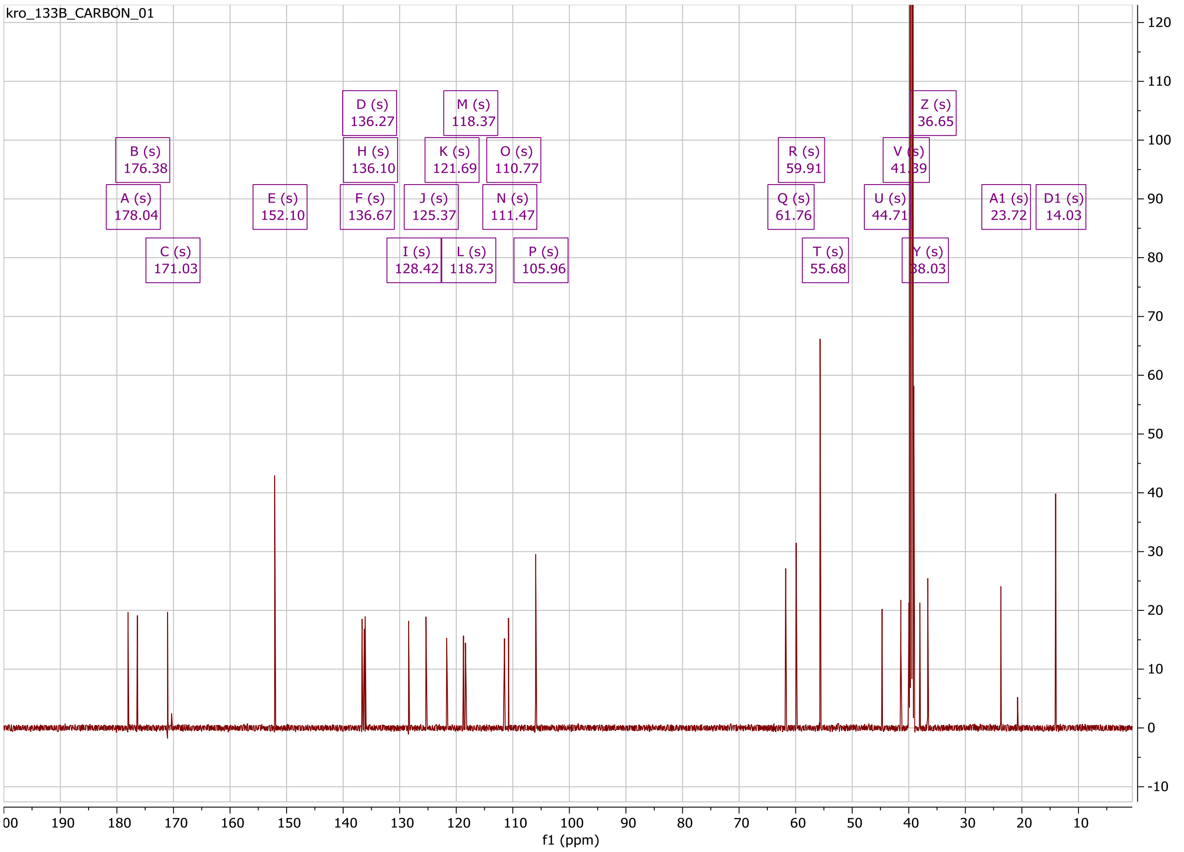

^{1}H NMR spectrum of **10** in $CDCl_3$

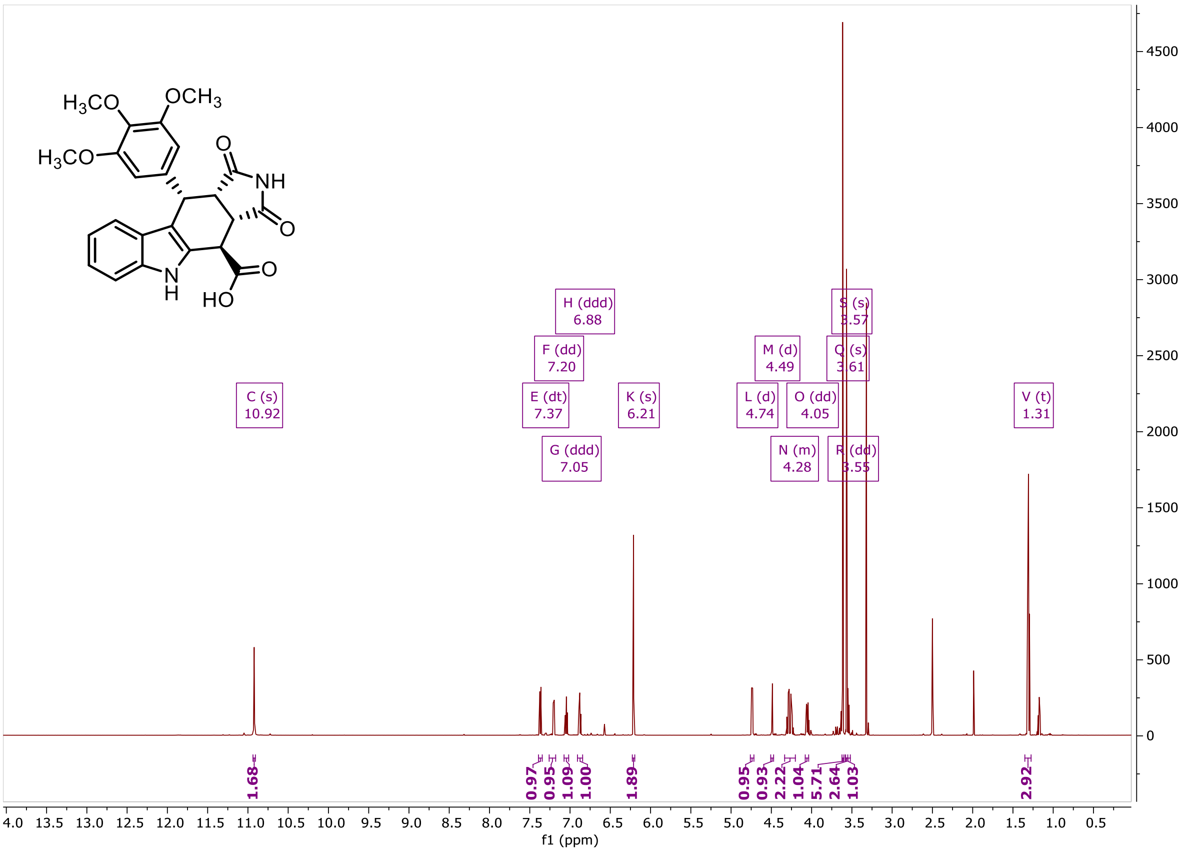

^{13}C NMR spectrum of (±)-**10** in DMSO-d_6.

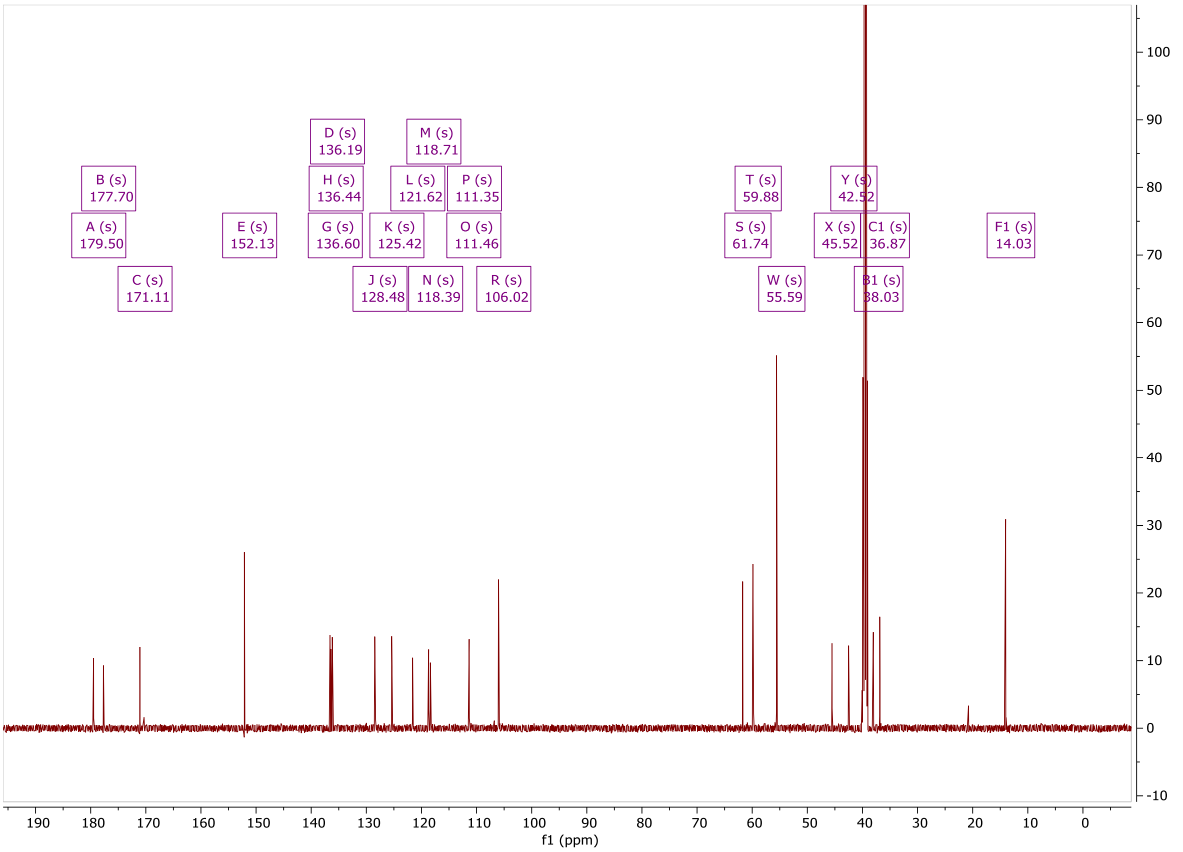

^{1}H NMR spectrum of **11** in $CDCl_3$

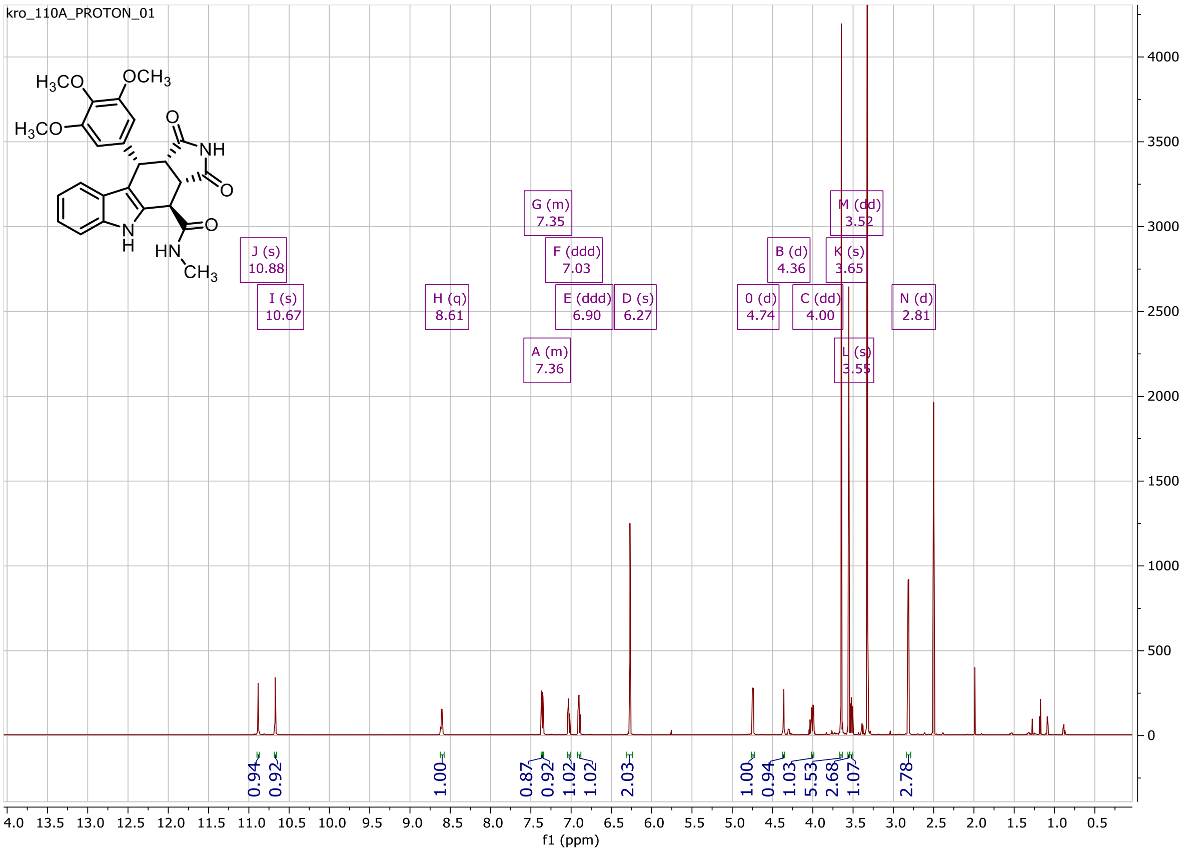

^{13}C NMR spectrum of (±)-**11** in DMSO-d_6.

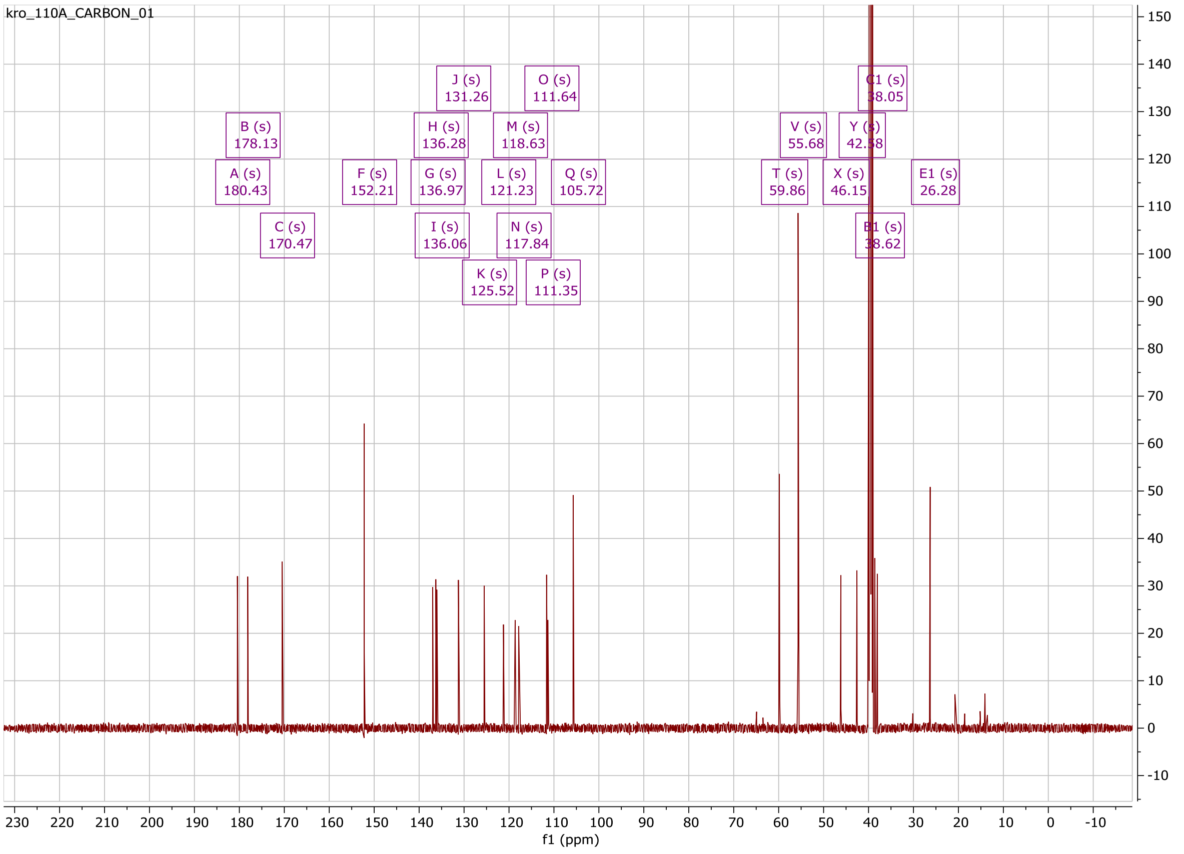

^{1}H NMR spectrum of **12** in $CDCl_3$

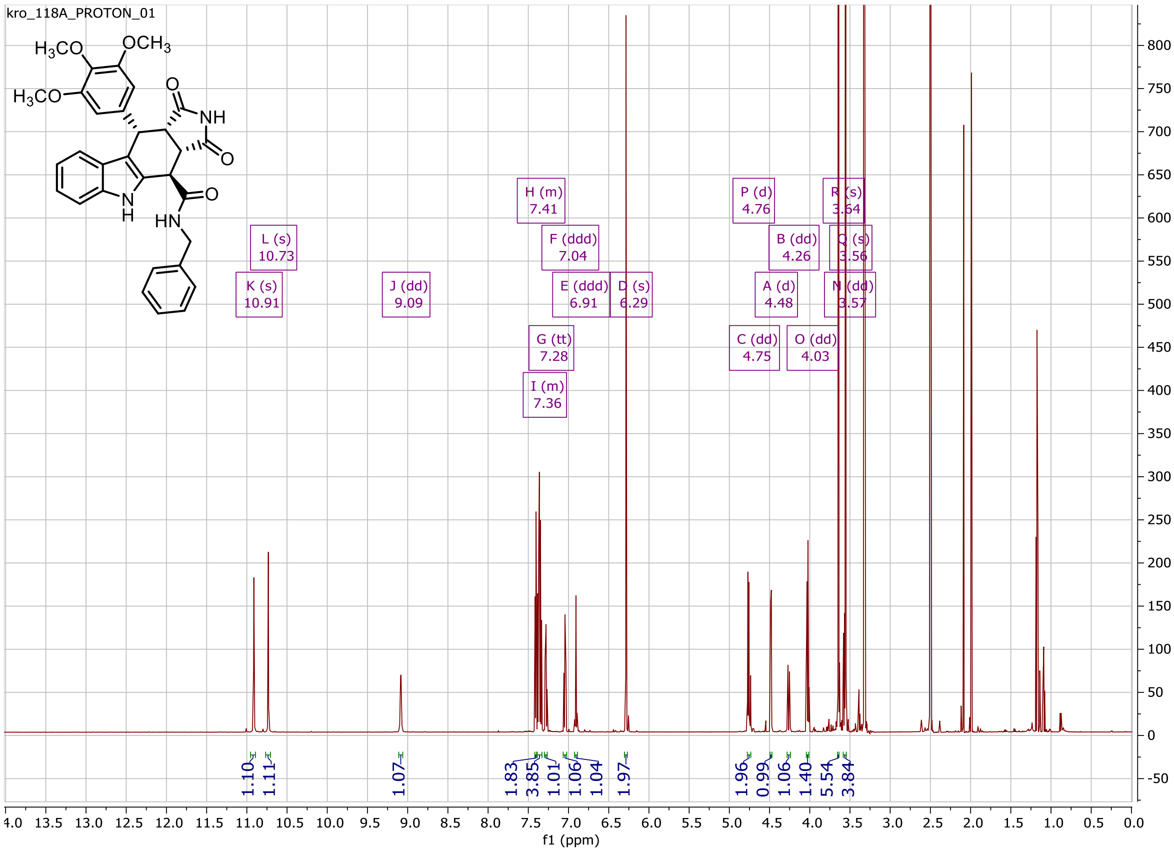

^{13}C NMR spectrum of (±)-**12** in DMSO-d_6.

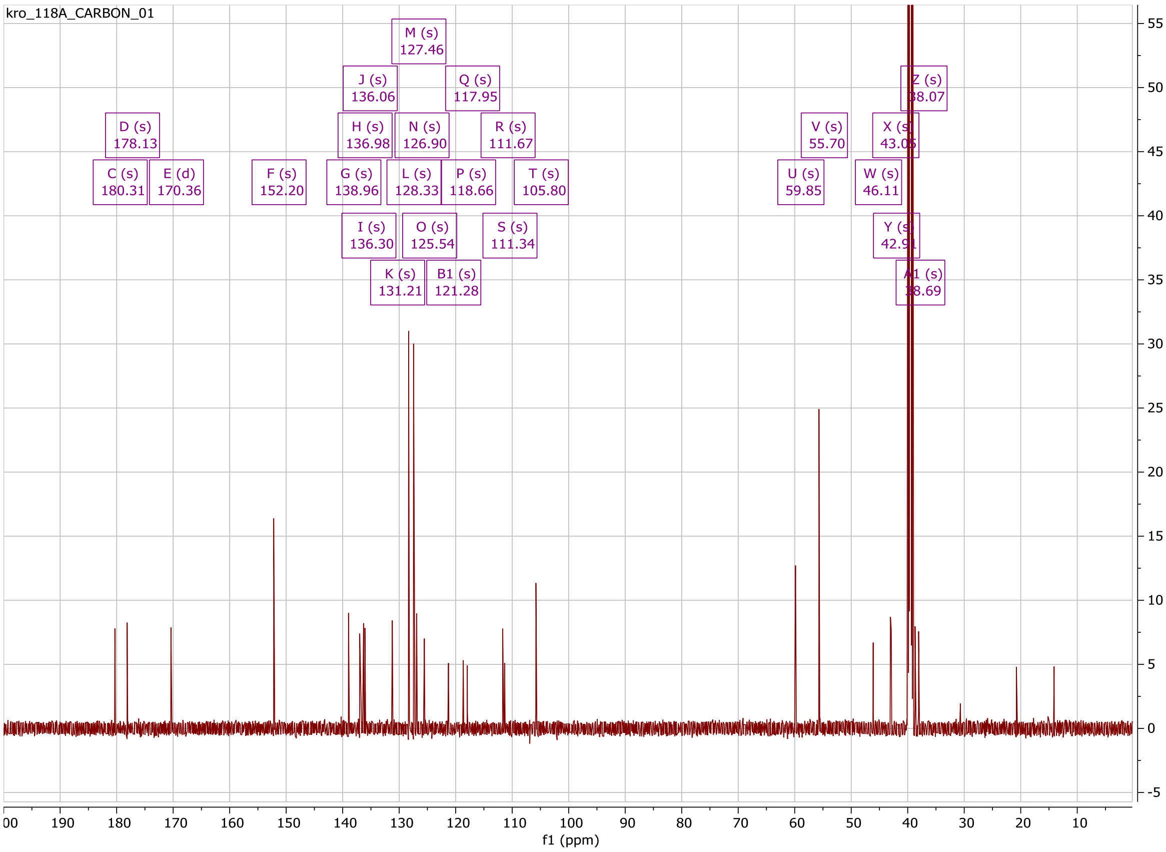

^{1}H NMR spectrum of **13** in $CDCl_3$

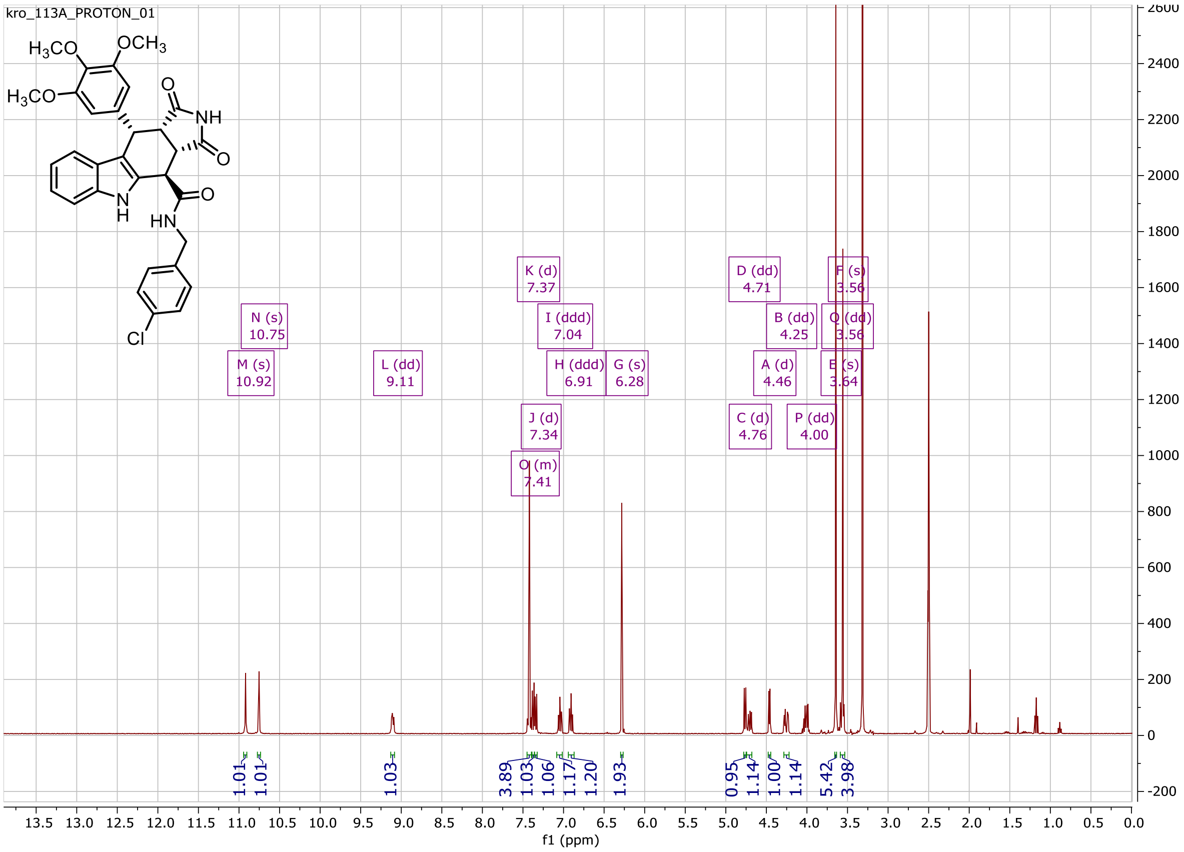

^{13}C NMR spectrum of (±)-**13** in DMSO-d_6.

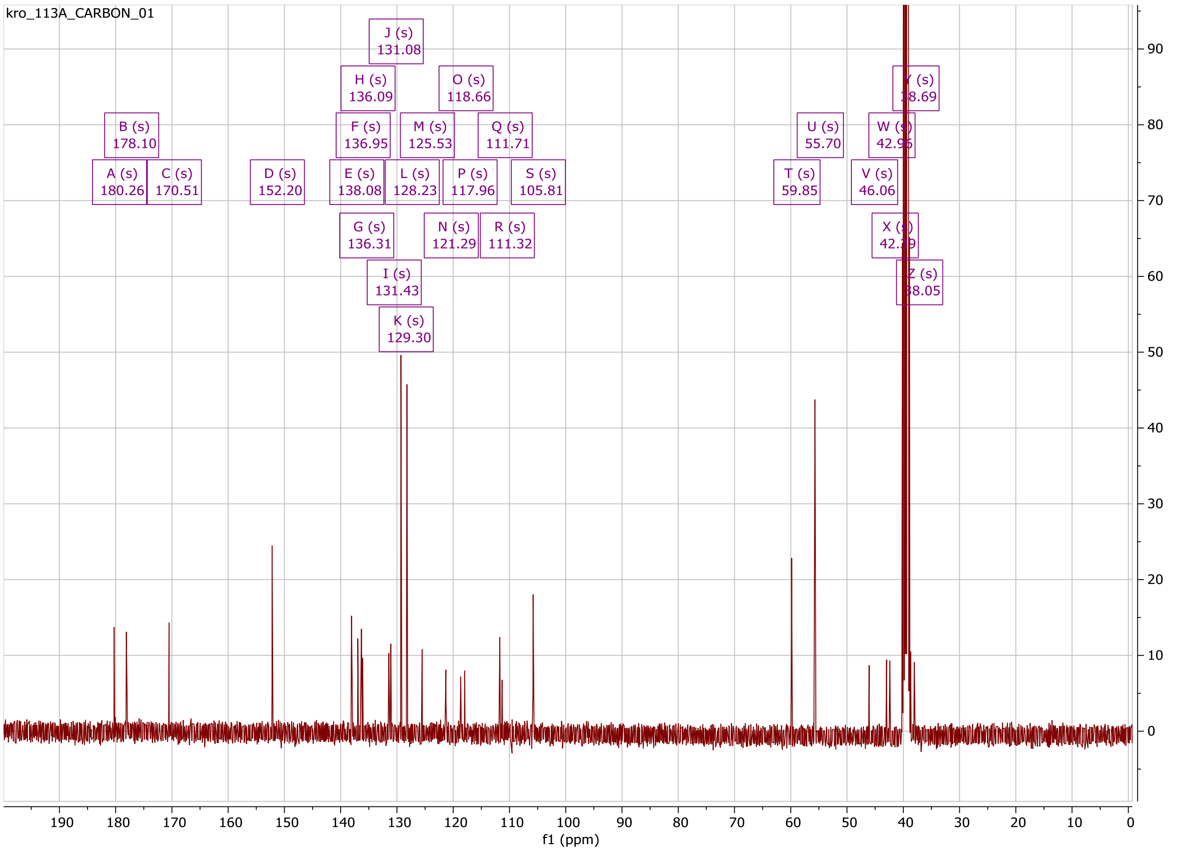

^{1}H NMR spectrum of **14** in $CDCl_3$

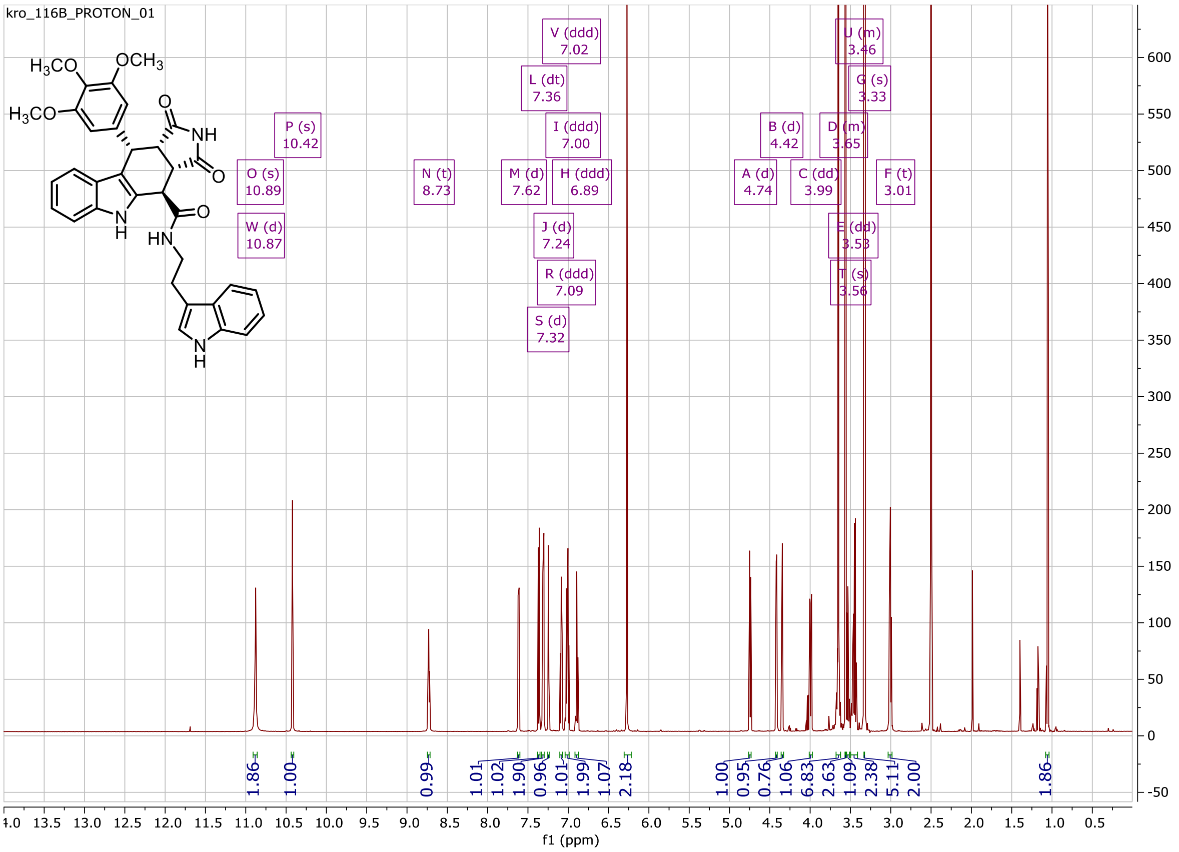

^{13}C NMR spectrum of (±)-**14** in DMSO-d_6.

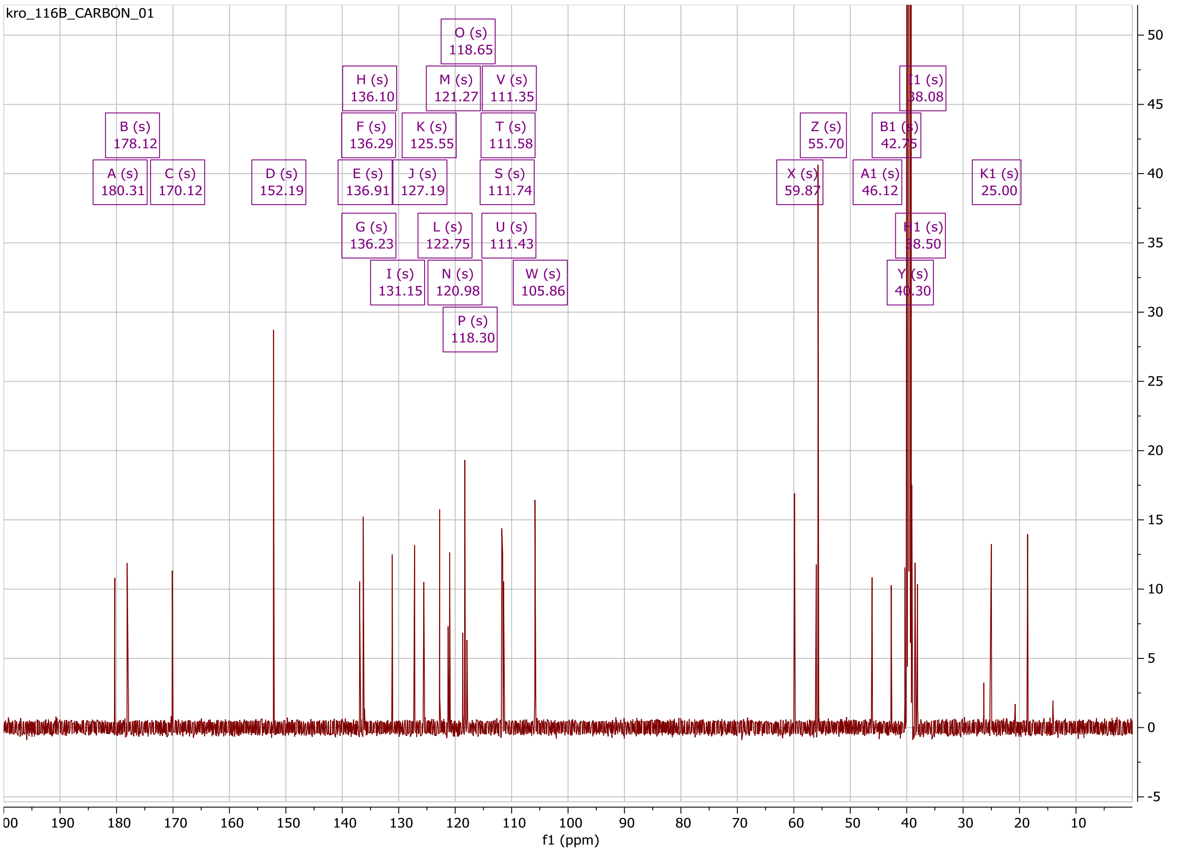

^{1}H NMR spectrum of **15** in $CDCl_3$

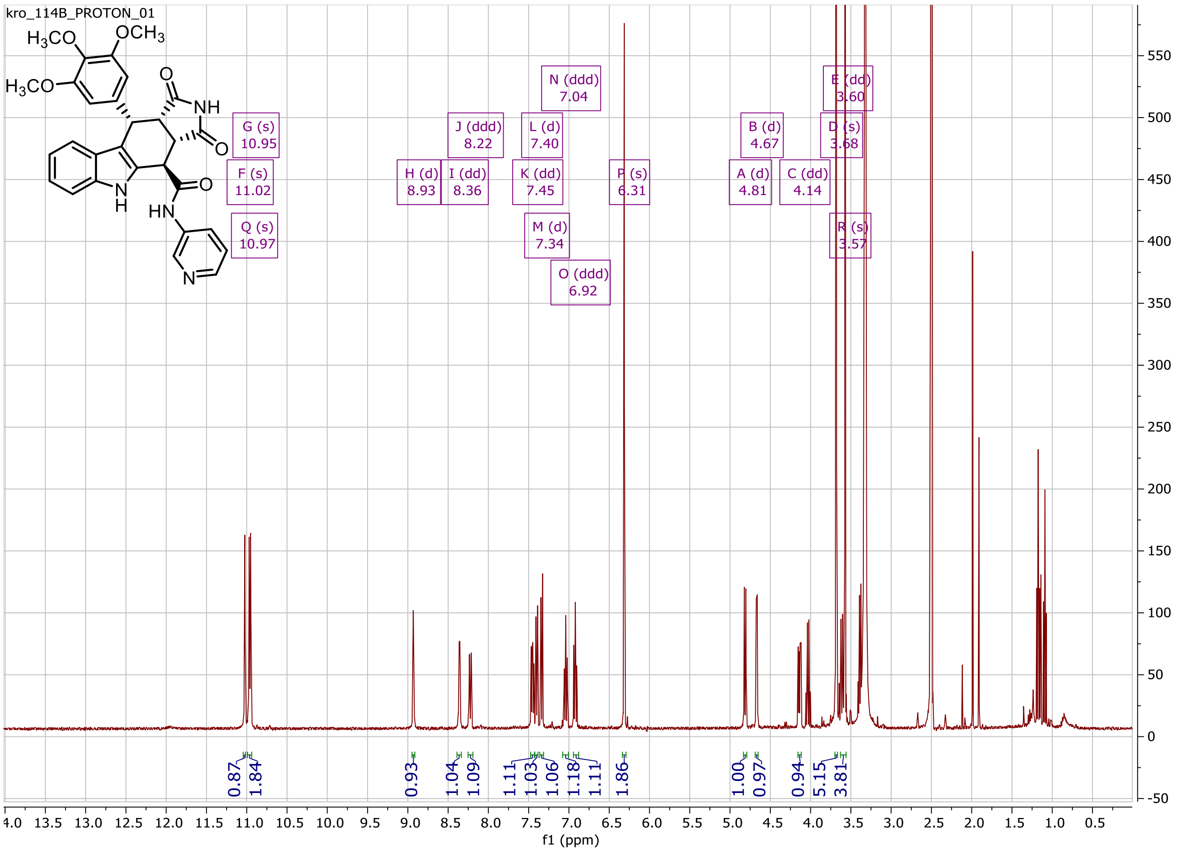

^{13}C NMR spectrum of (±)-**15** in DMSO-d_6.

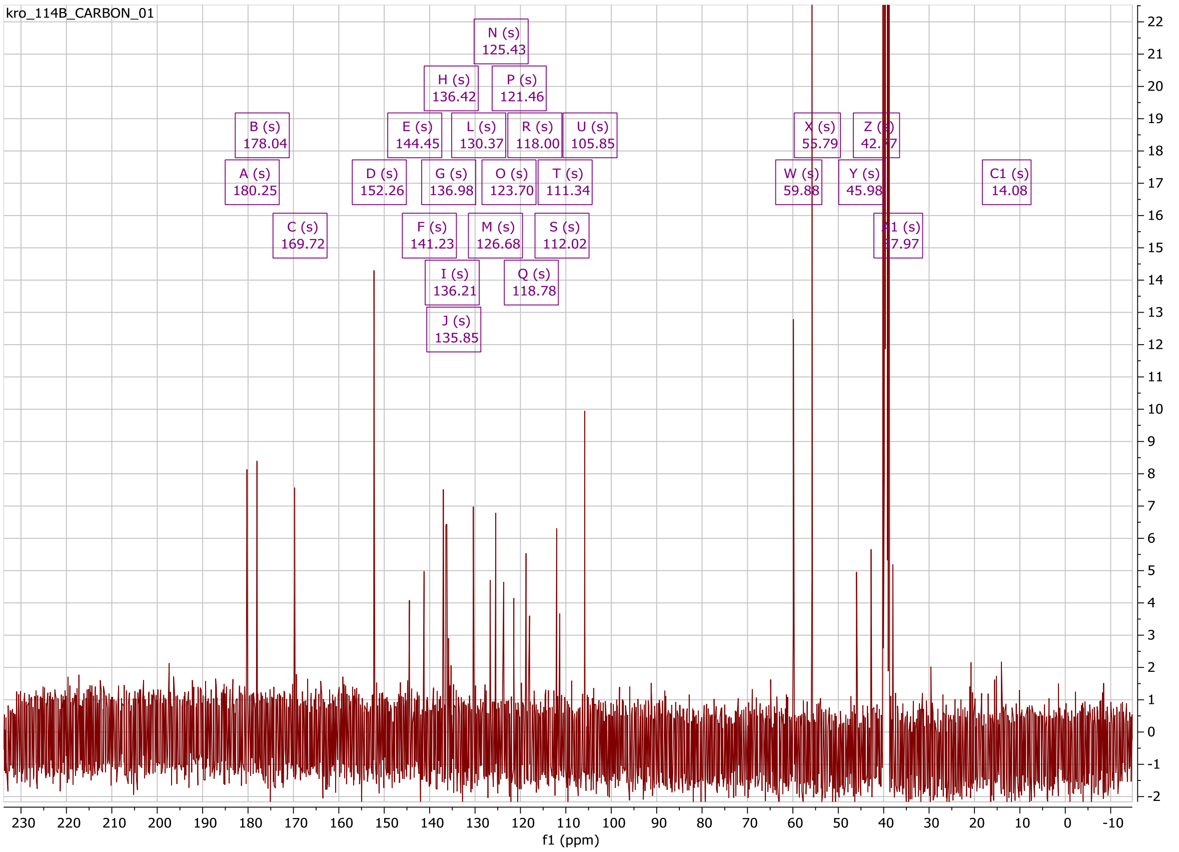

^{1}H NMR spectrum of **16** in $CDCl_3$

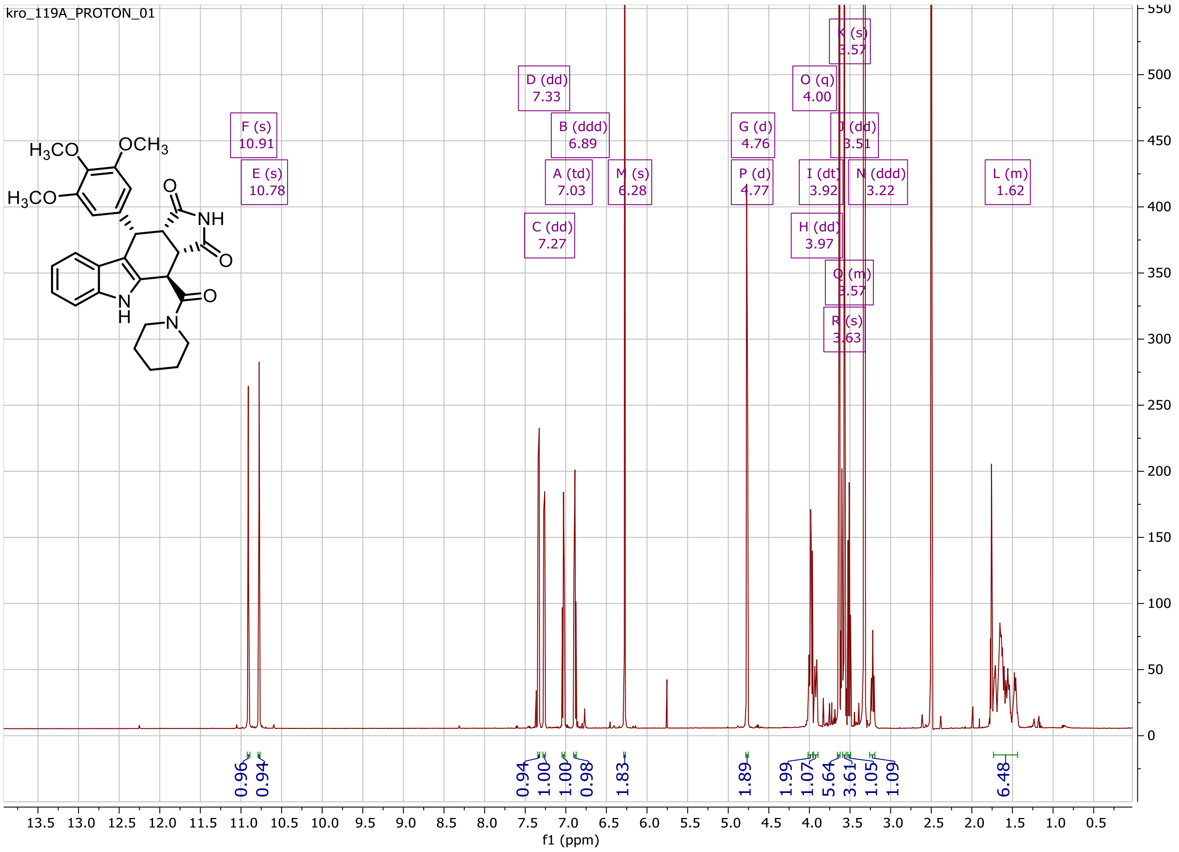

^{13}C NMR spectrum of (±)-**16** in DMSO-d_6.

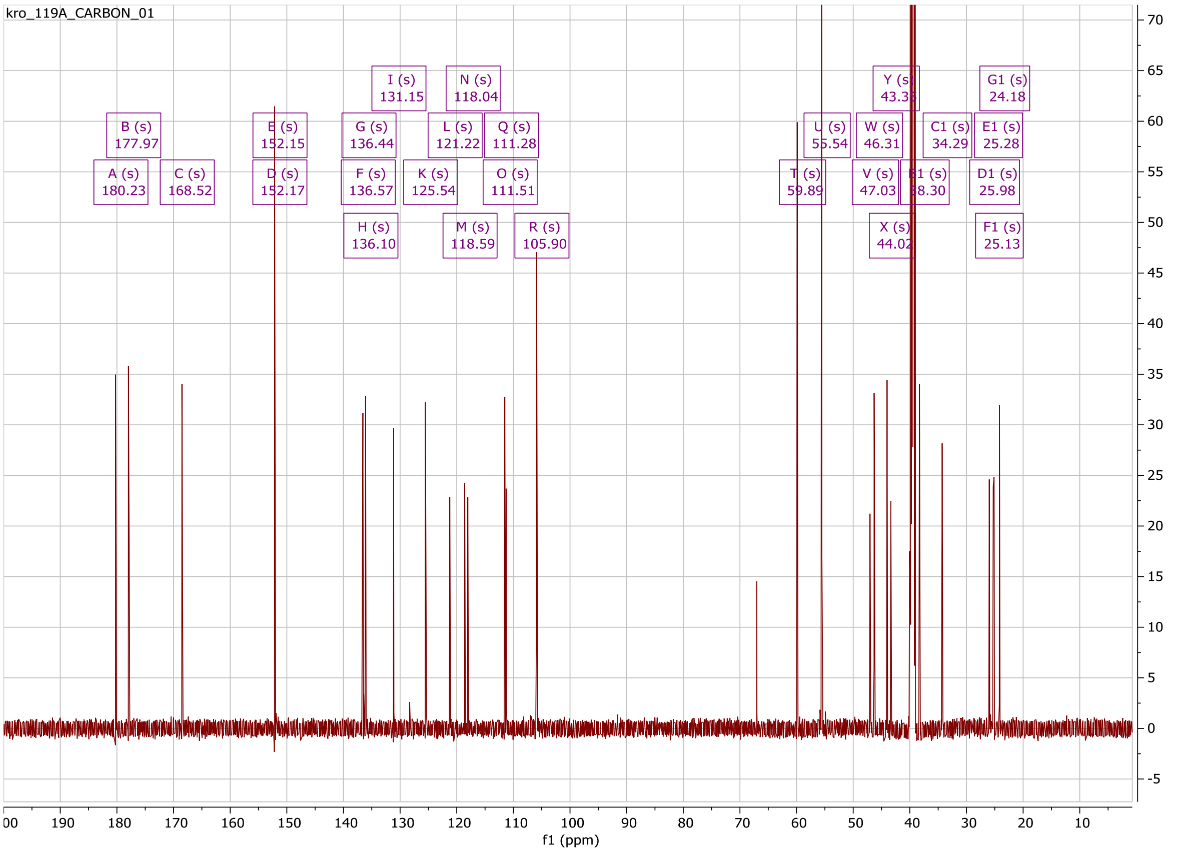

^{1}H NMR spectrum of **17** in $CDCl_3$

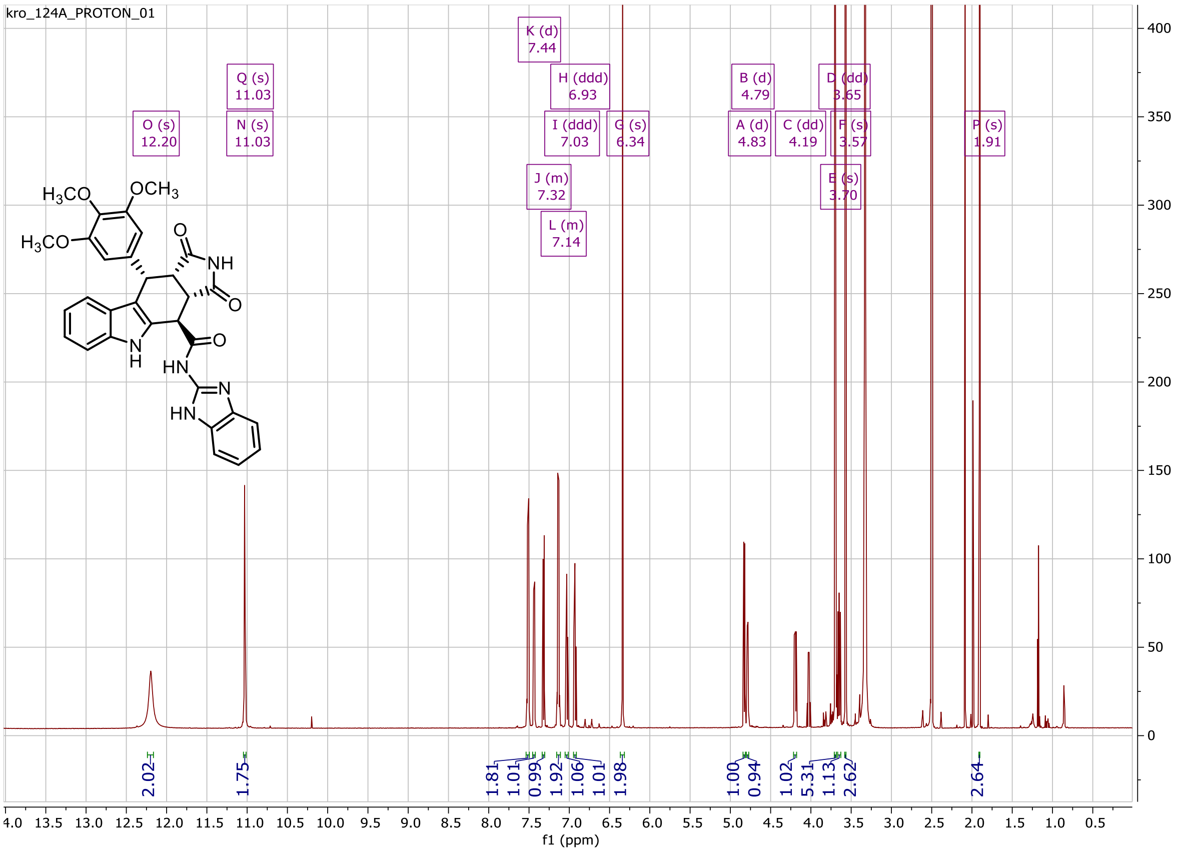

^{13}C NMR spectrum of (±)-**17** in DMSO-*d*$_6$.

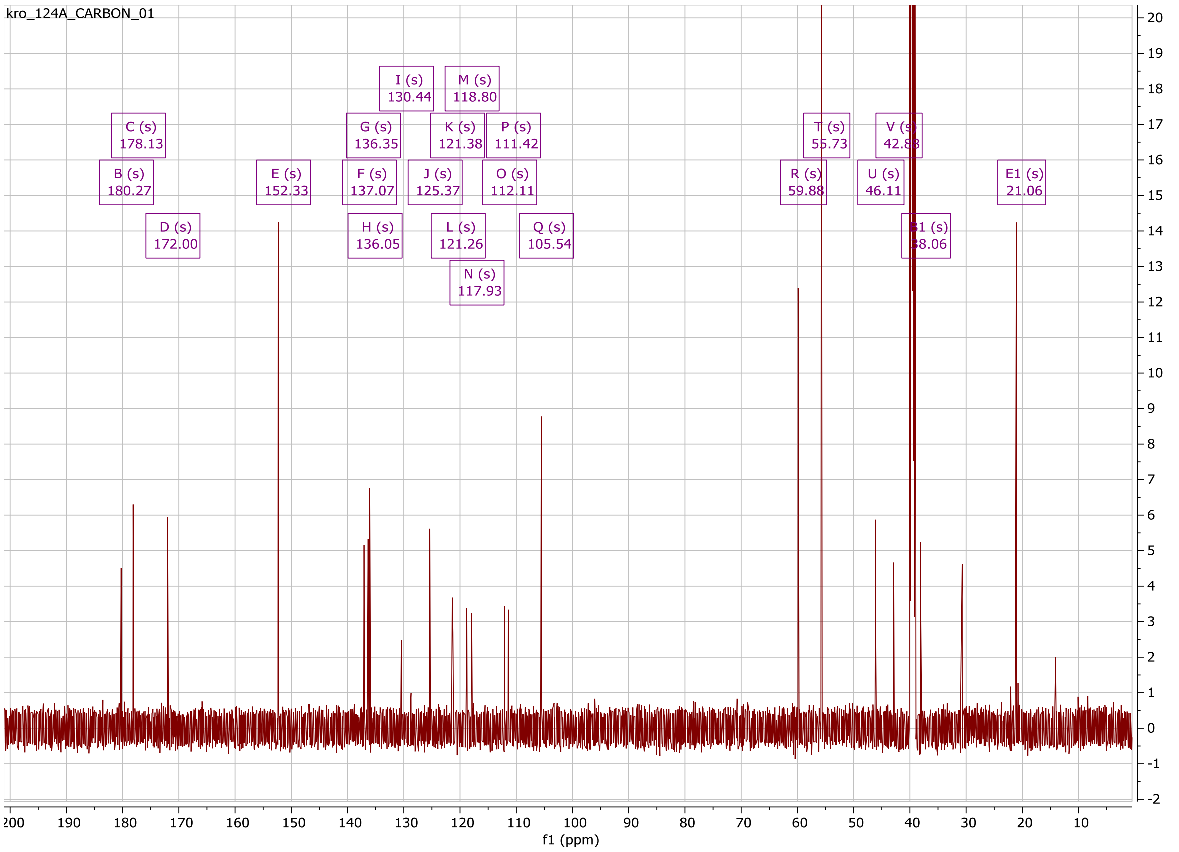

^{1}H NMR spectrum of **18a** in $CDCl_3$

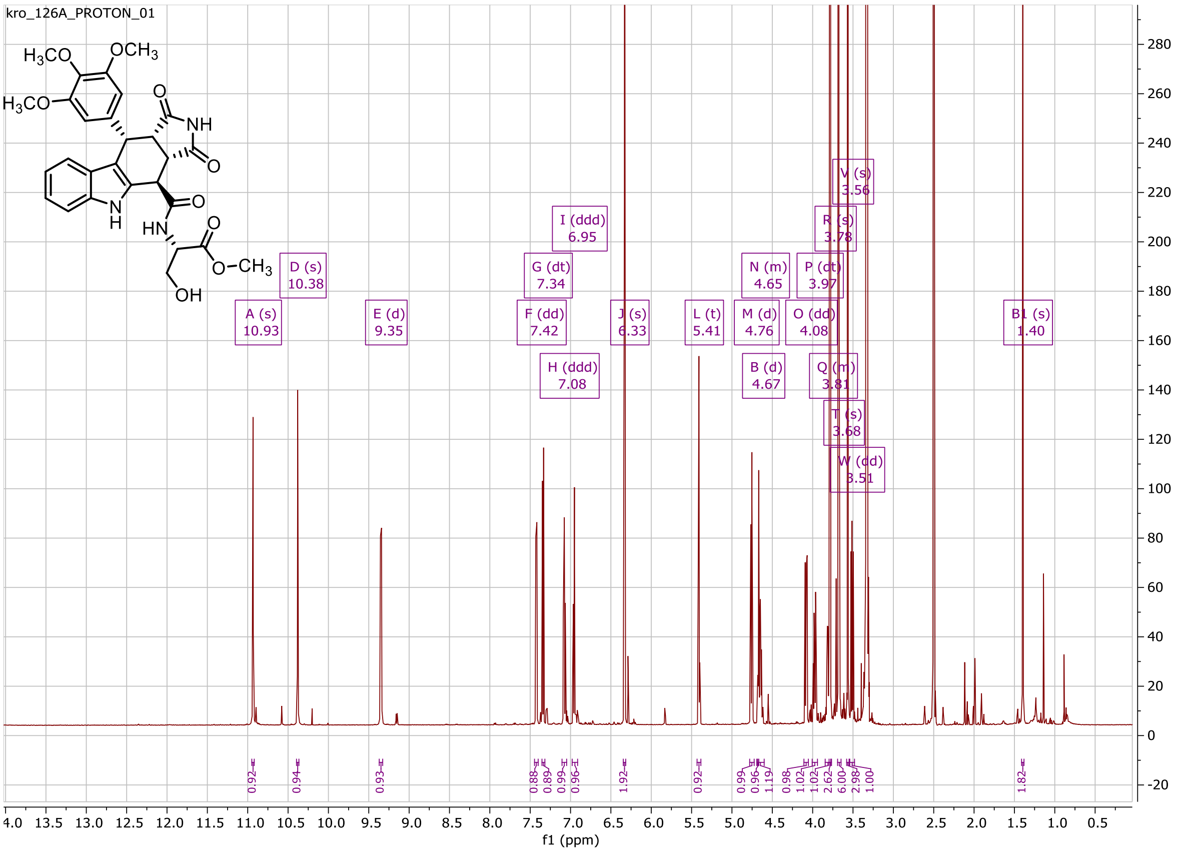

^{13}C NMR spectrum of (+)-**18a** in DMSO-d_6.

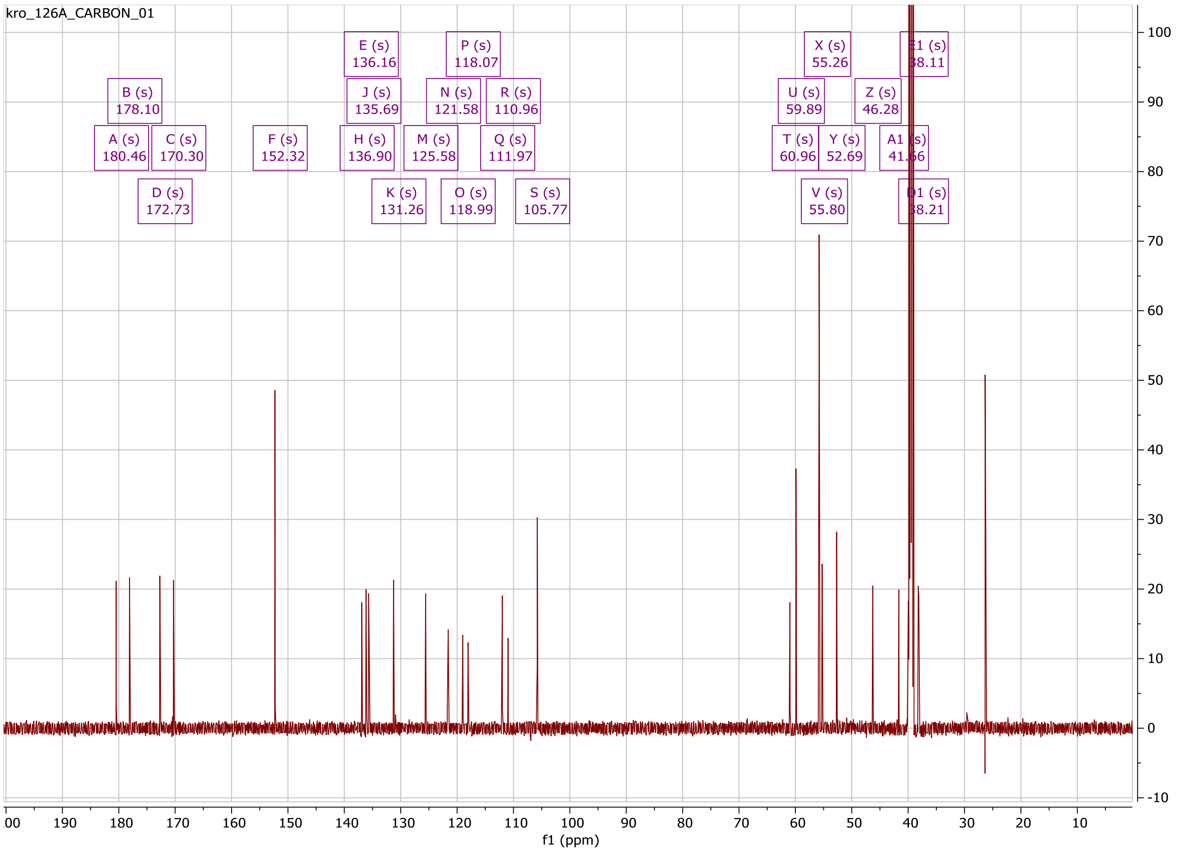

^{1}H NMR spectrum of **18b** in $CDCl_3$

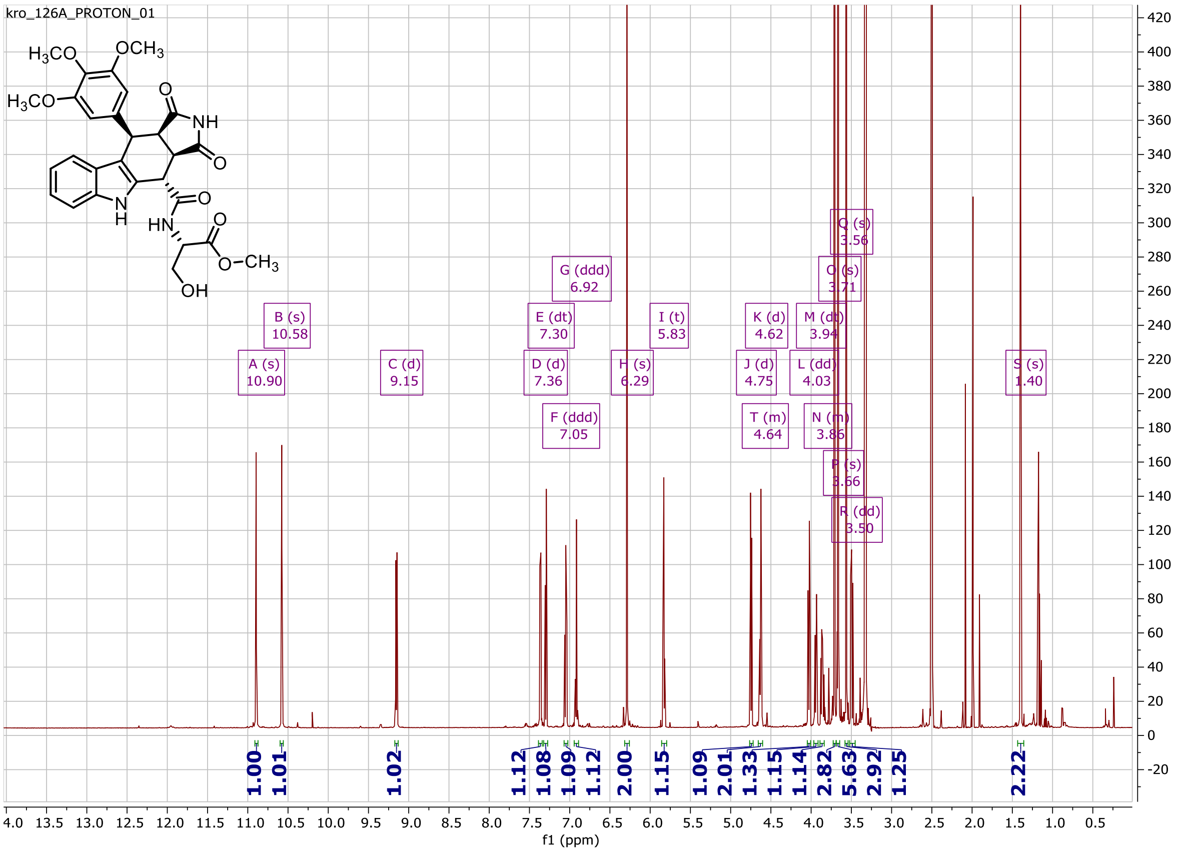

^{13}C NMR spectrum of (-)-**18b** in DMSO-d_6.

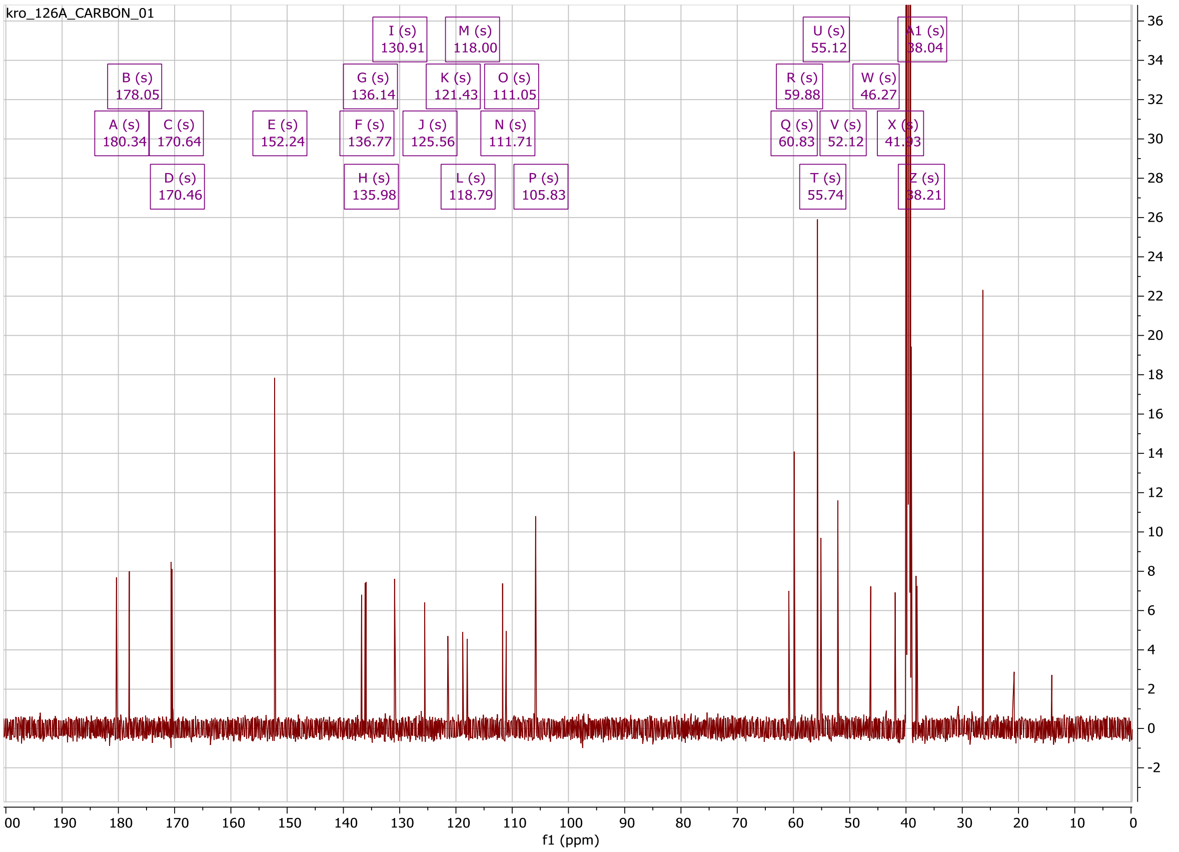

^{1}H NMR spectrum of **19b** in $CDCl_3$

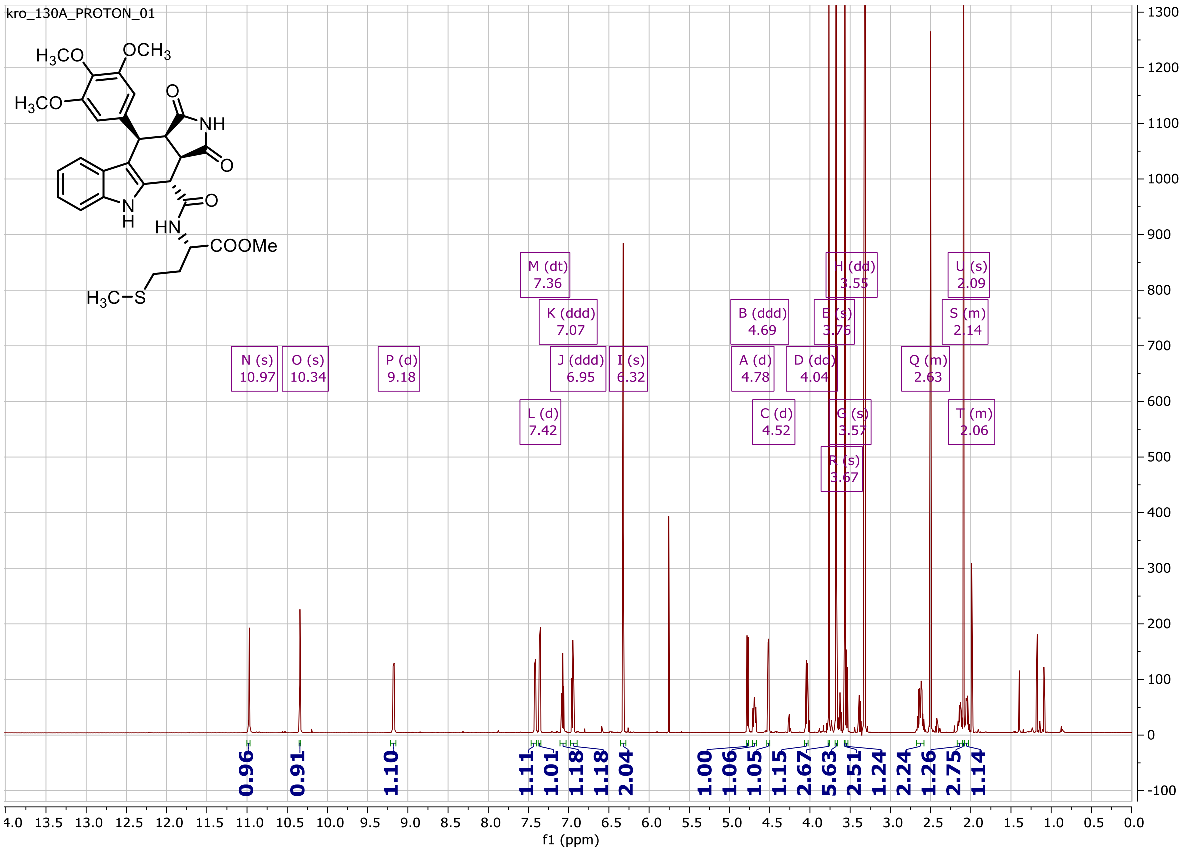

^{13}C NMR spectrum of (-)-**19b** in DMSO-d_6.

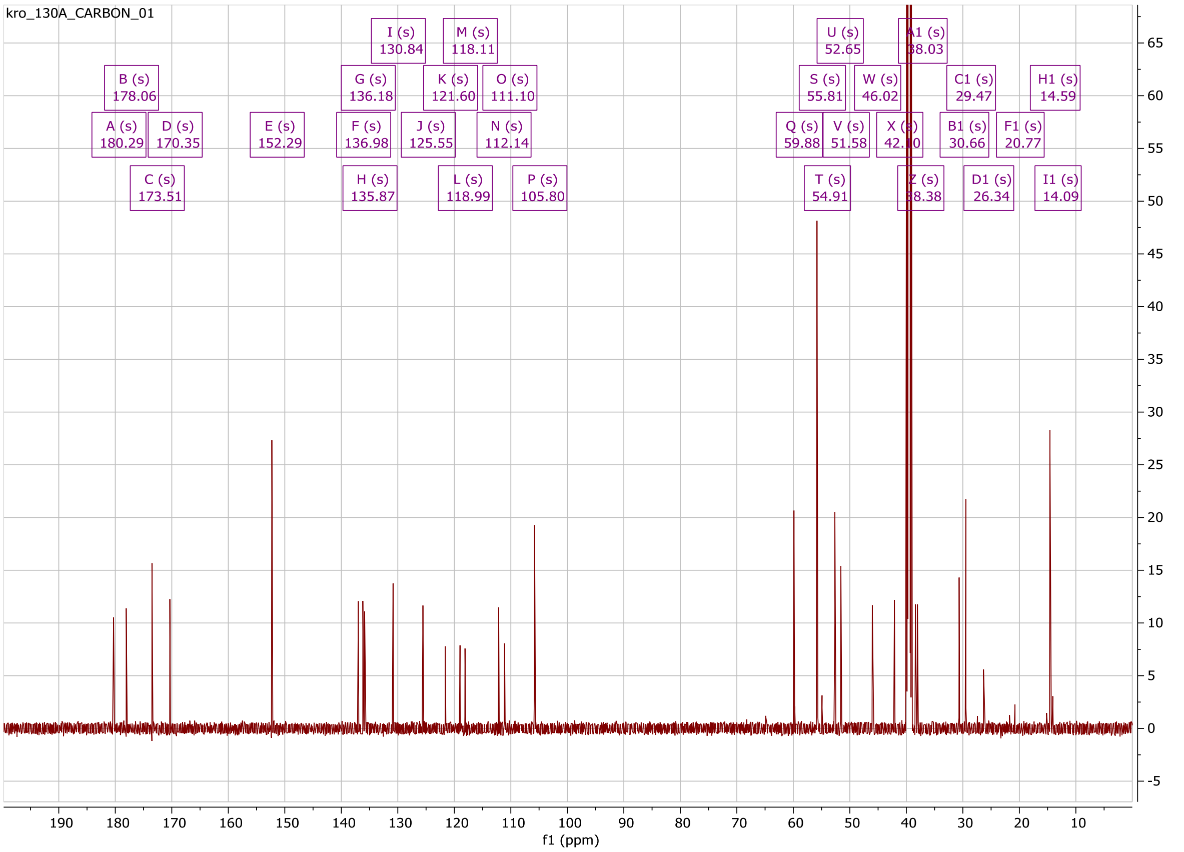

^{1}H NMR spectrum of **20a** in $CDCl_3$

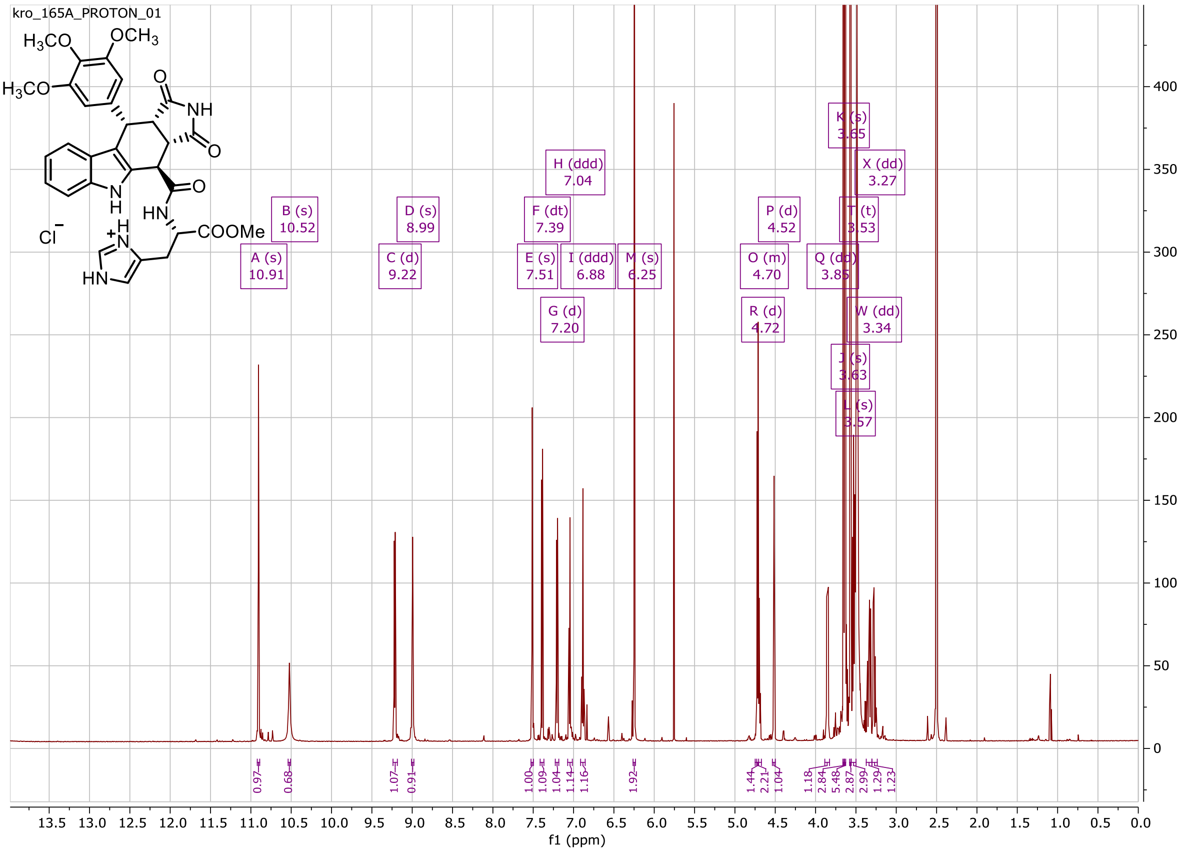

^{13}C NMR spectrum of (+)-**20a**·HCl in DMSO-d_6.

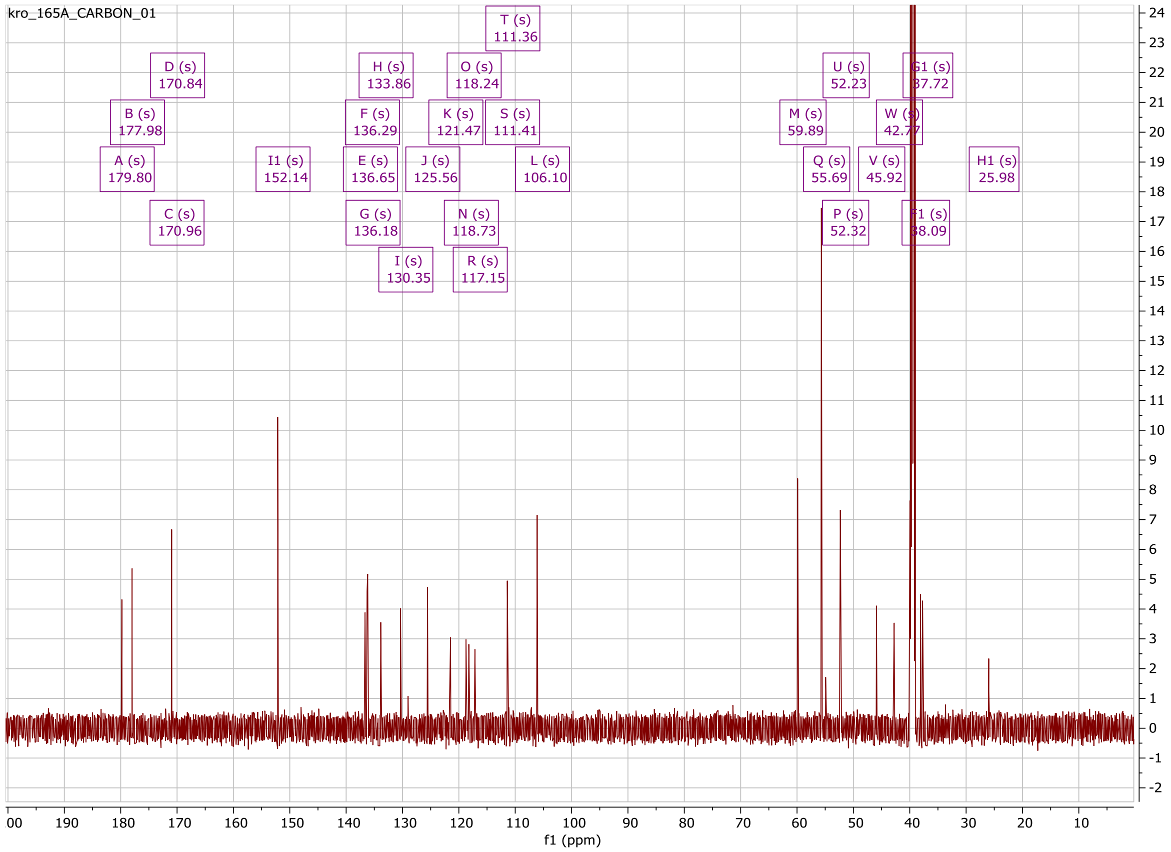

^{1}H NMR spectrum of **20b** in $CDCl_3$

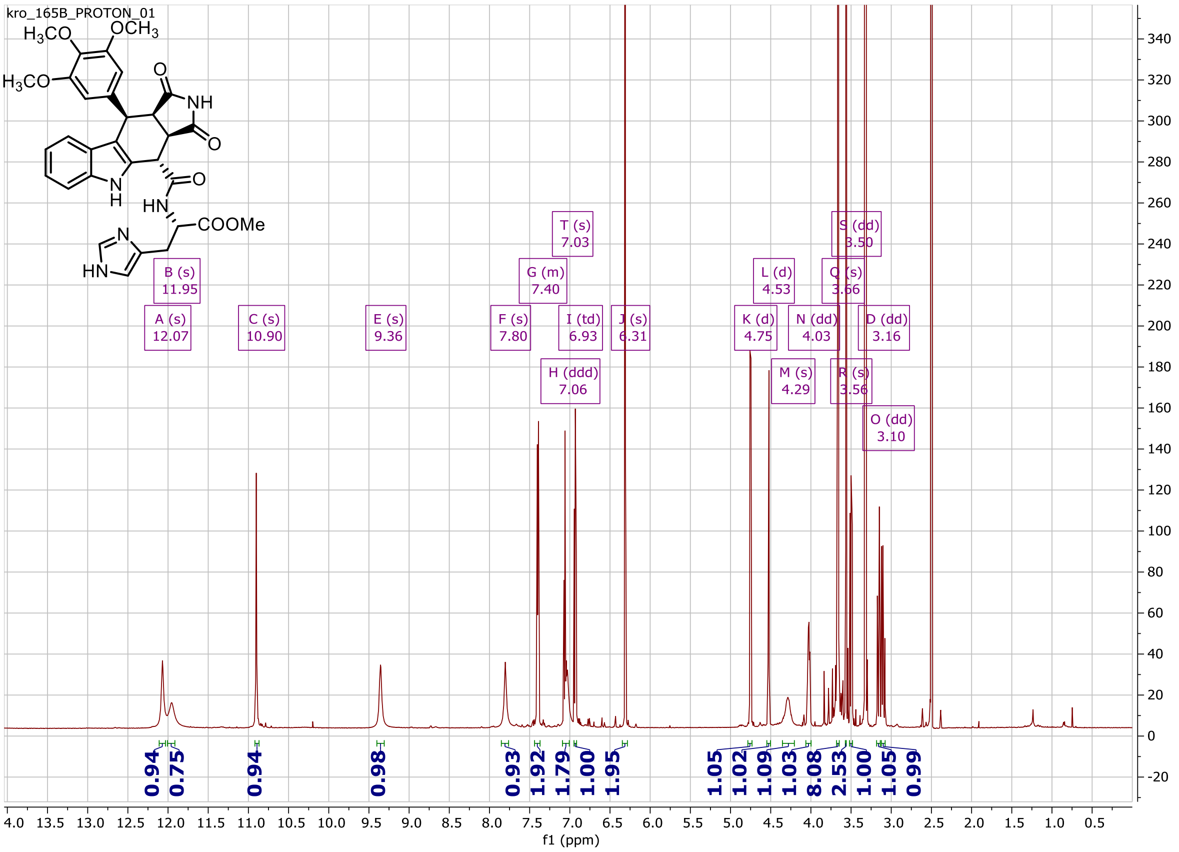

^{13}C NMR spectrum of (-)-**20b** in DMSO-d_6.

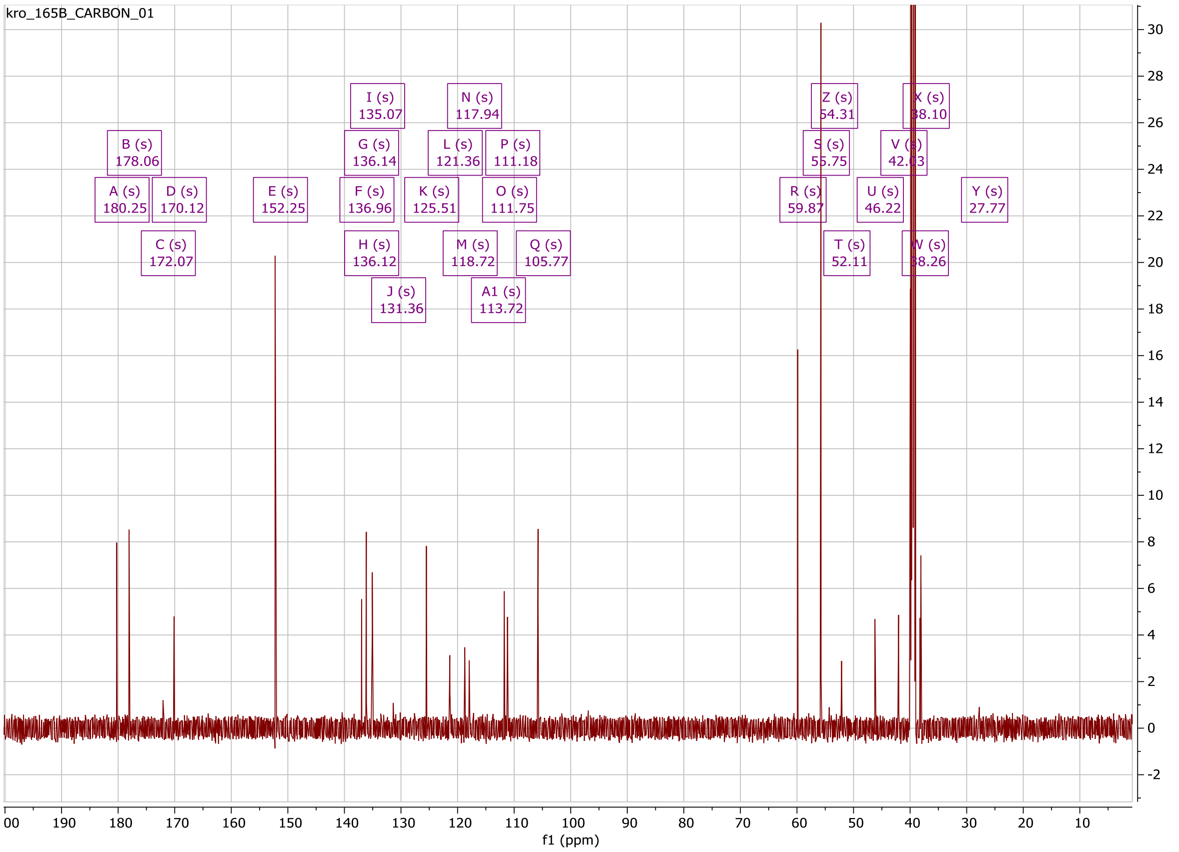

^{1}H NMR spectrum of **21a** in $CDCl_3$

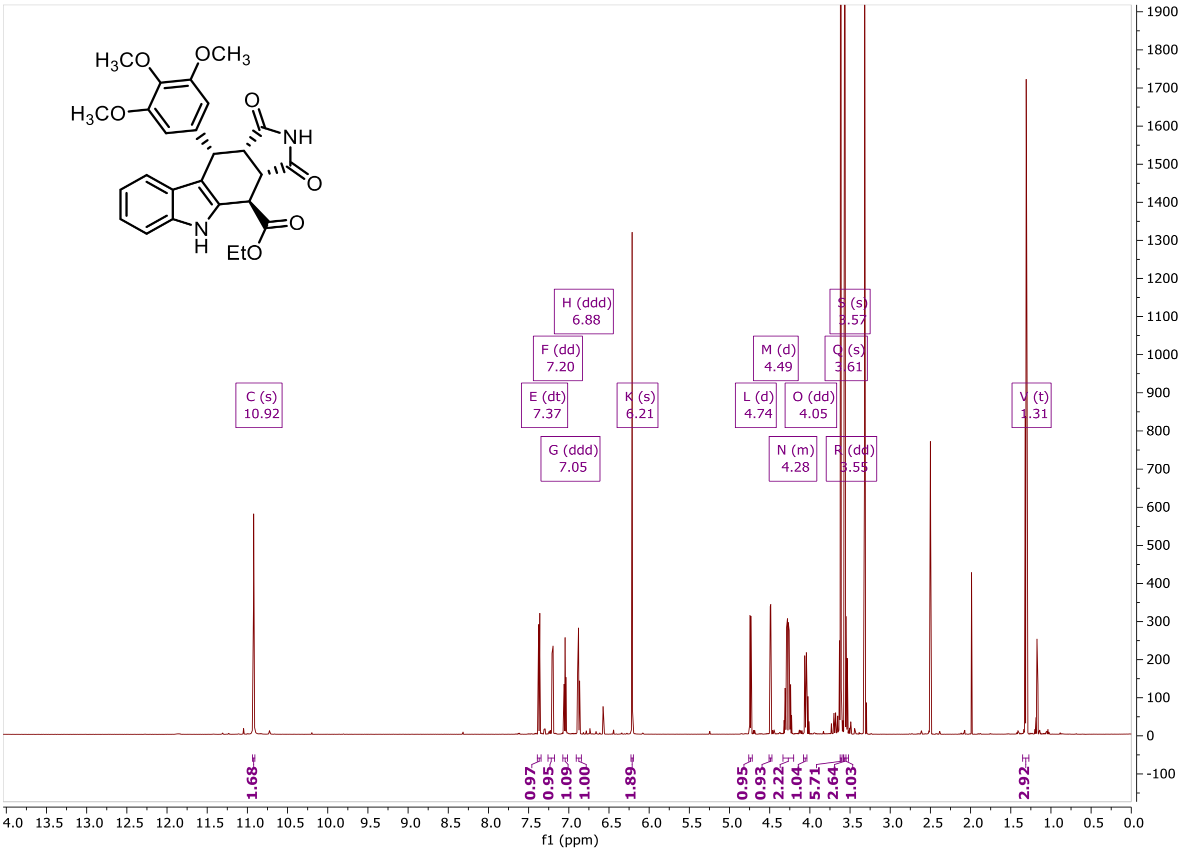

^{13}C NMR spectrum of (±)-**21a** in DMSO-d_6.

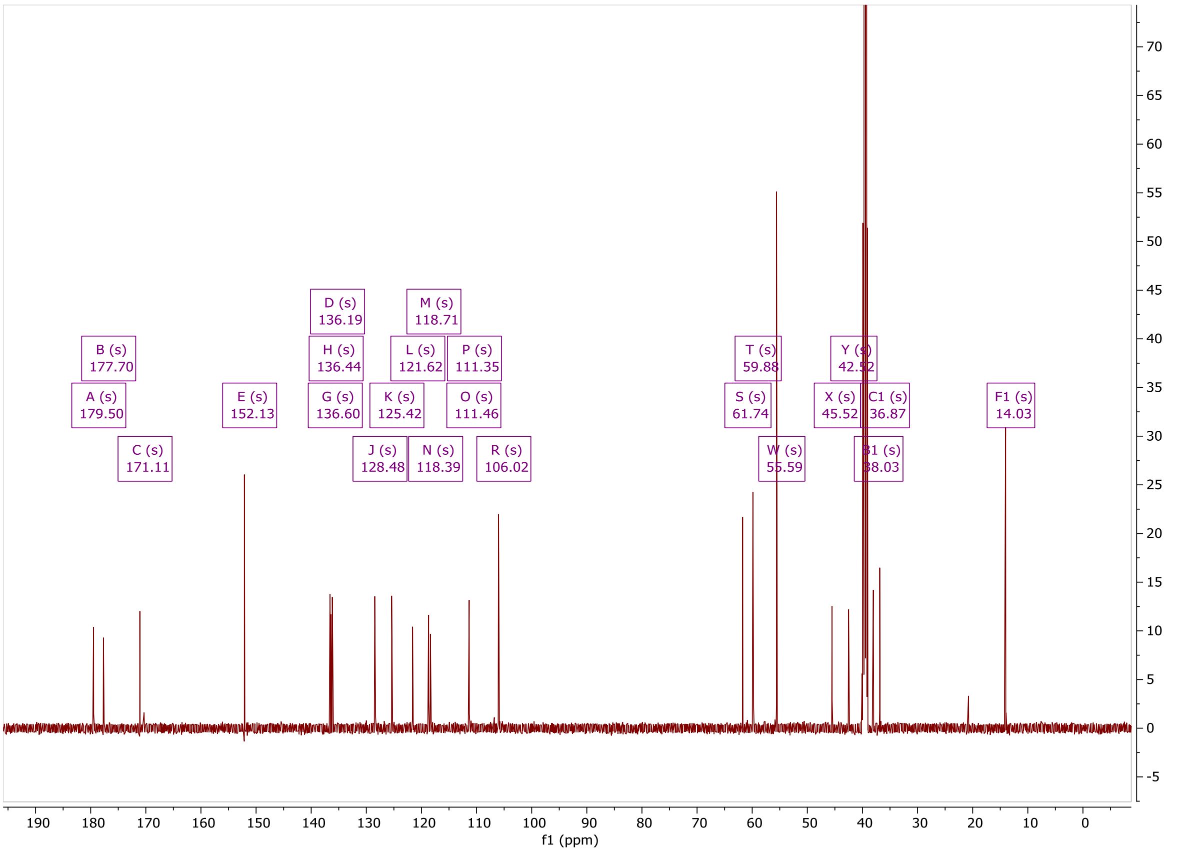

^{1}H NMR spectrum of **21b** in $CDCl_3$

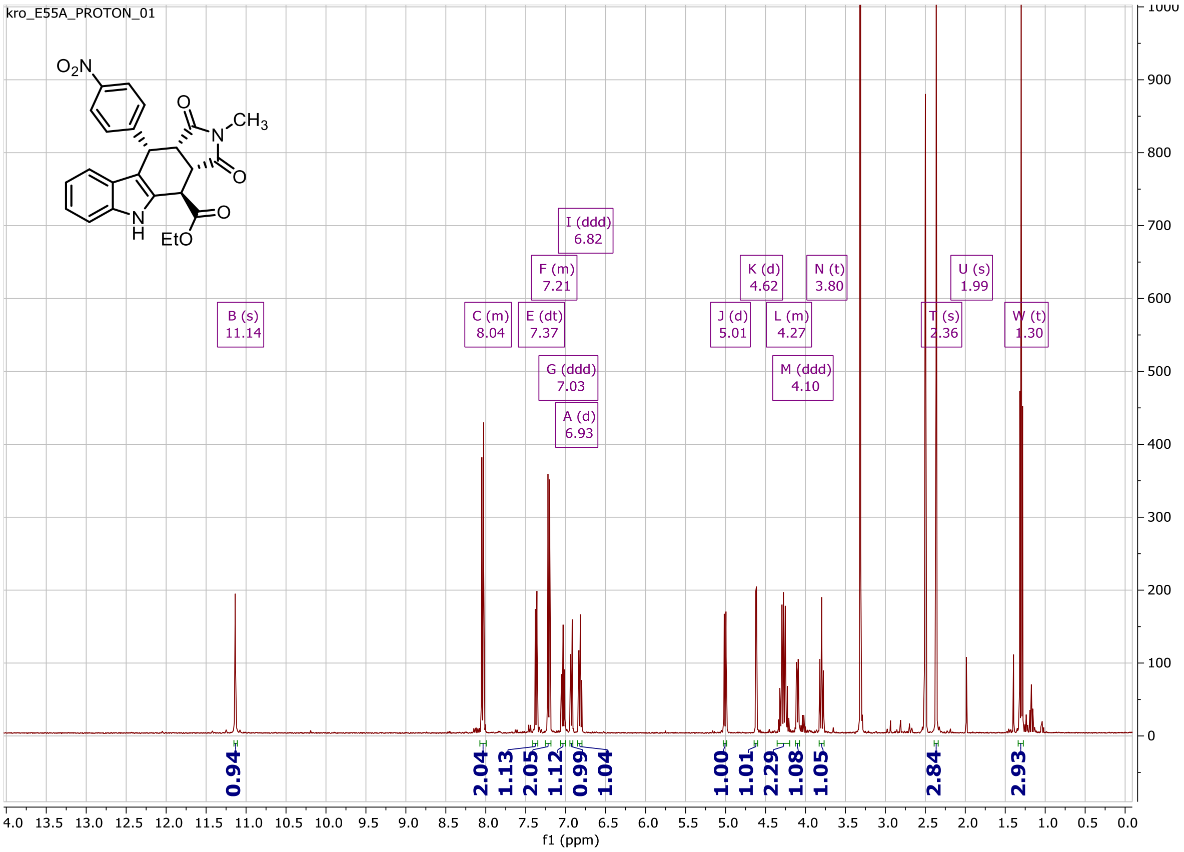

^{13}C NMR spectrum of (±)-**21b** in DMSO-d_6.

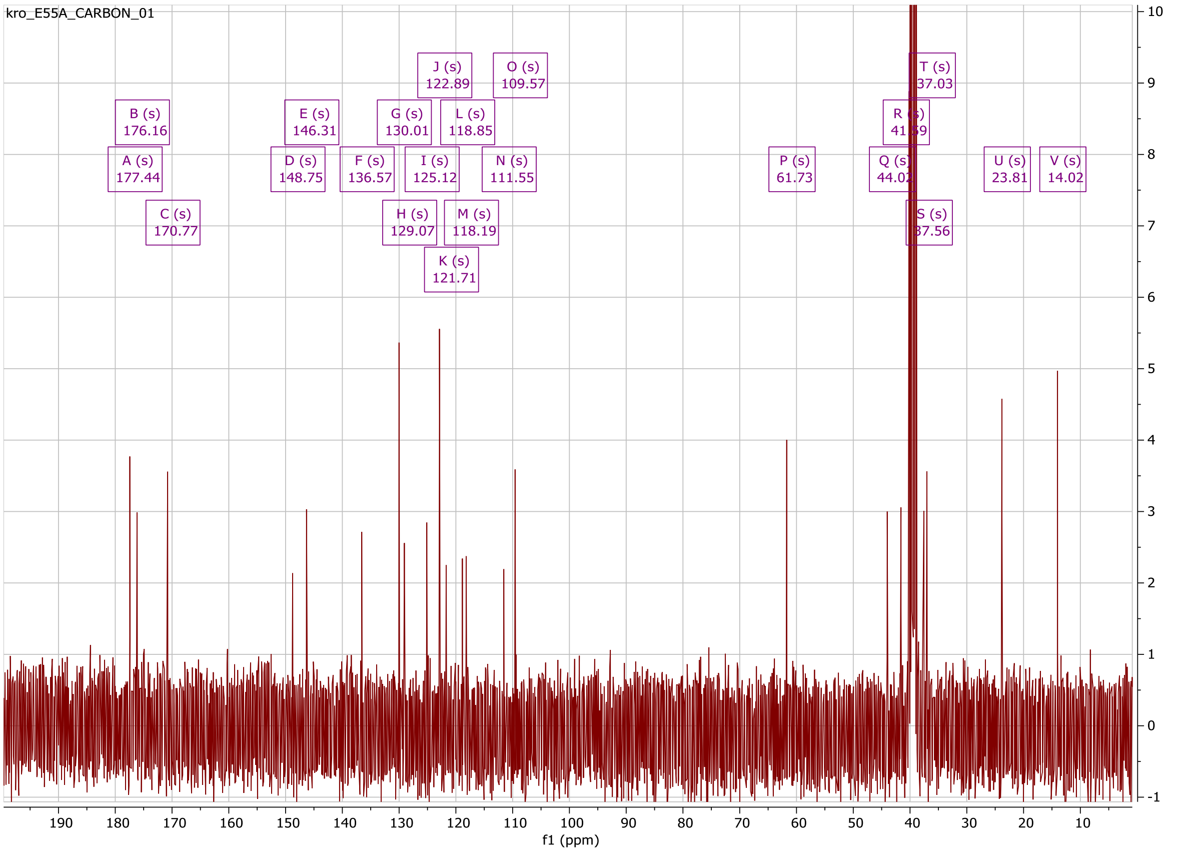

^{1}H NMR spectrum of **21c** in $CDCl_3$

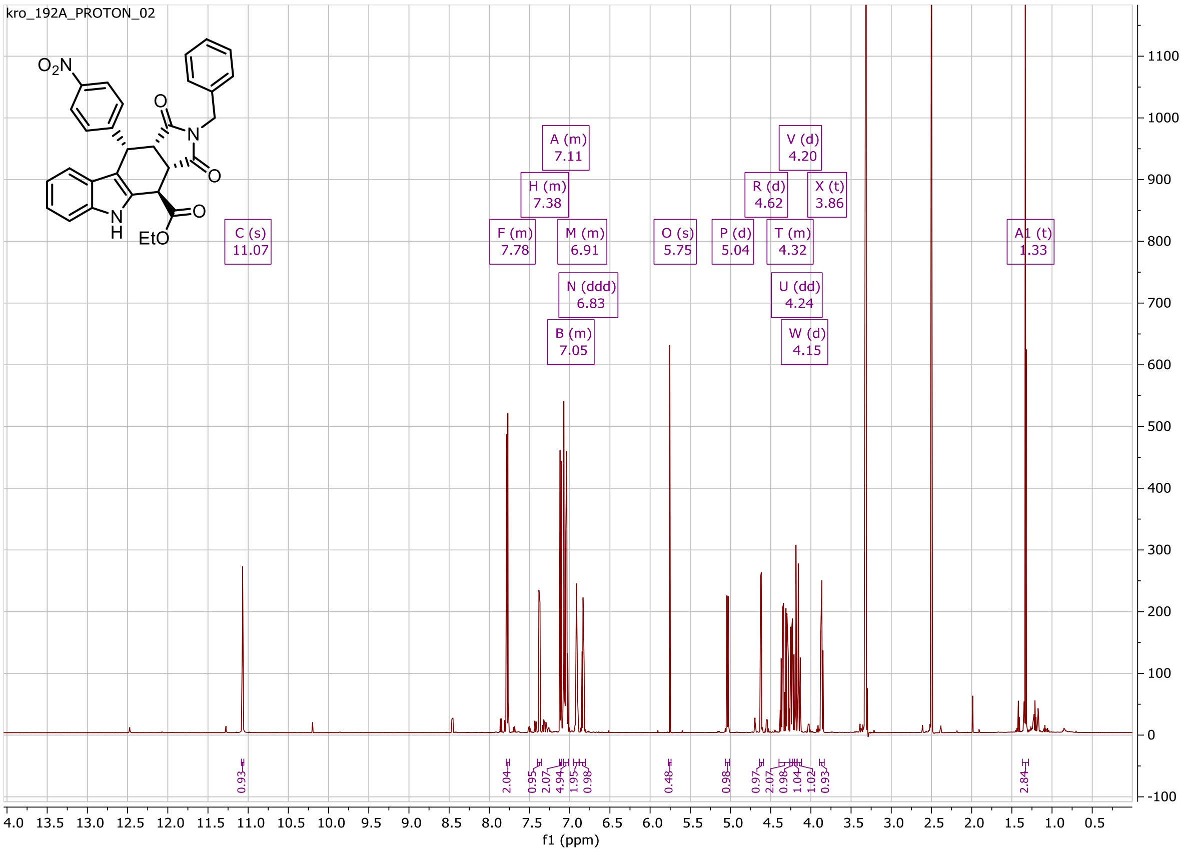

^{13}C NMR spectrum of (±)-**21c** in DMSO-d_6.

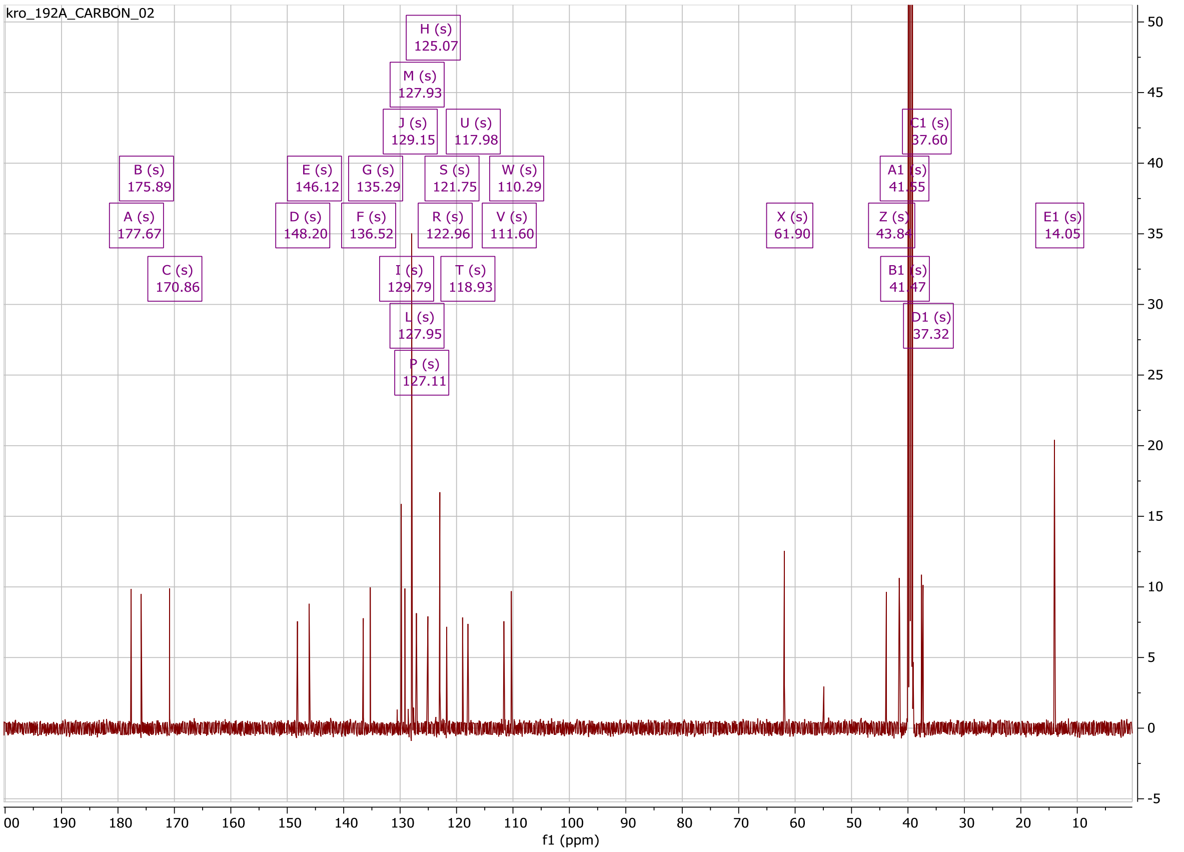

^{1}H NMR spectrum of **22a** in $CDCl_3$

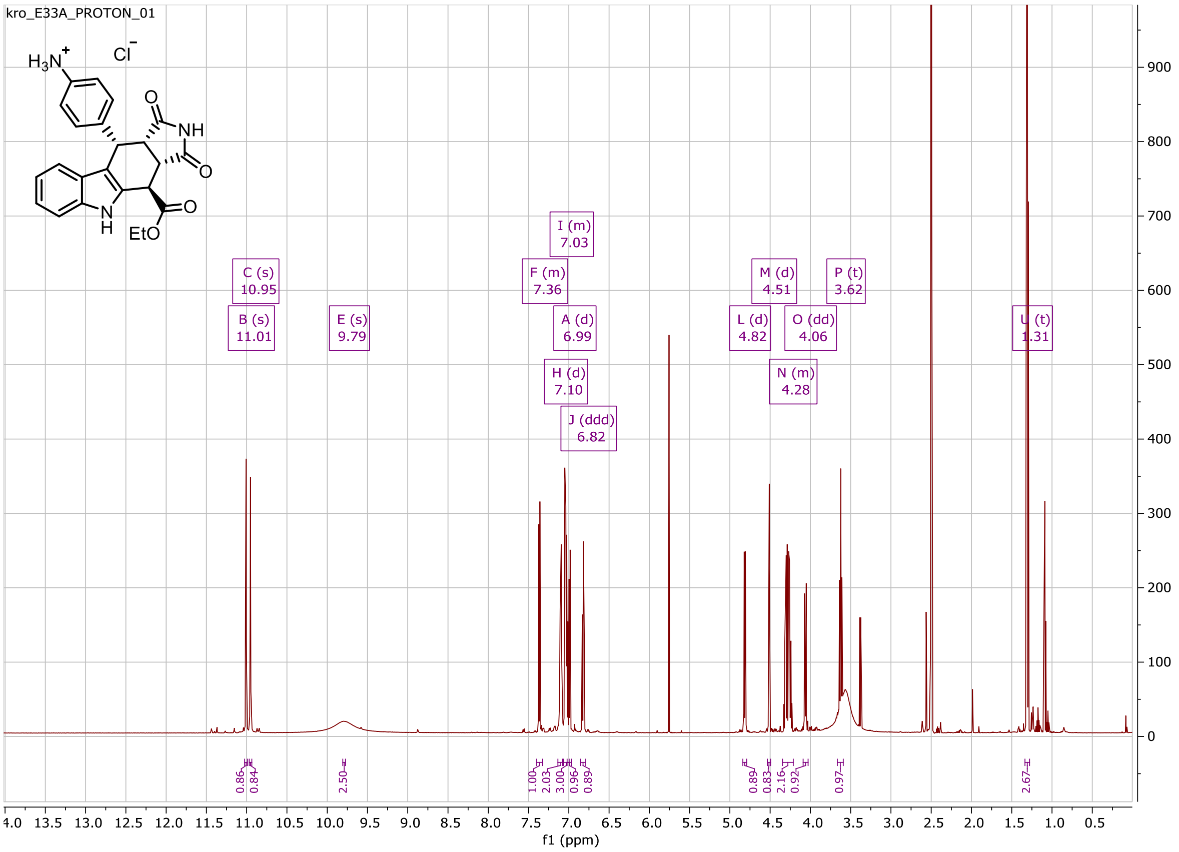

^{13}C NMR spectrum of (±)-**22a**·HCl in DMSO-d_6.

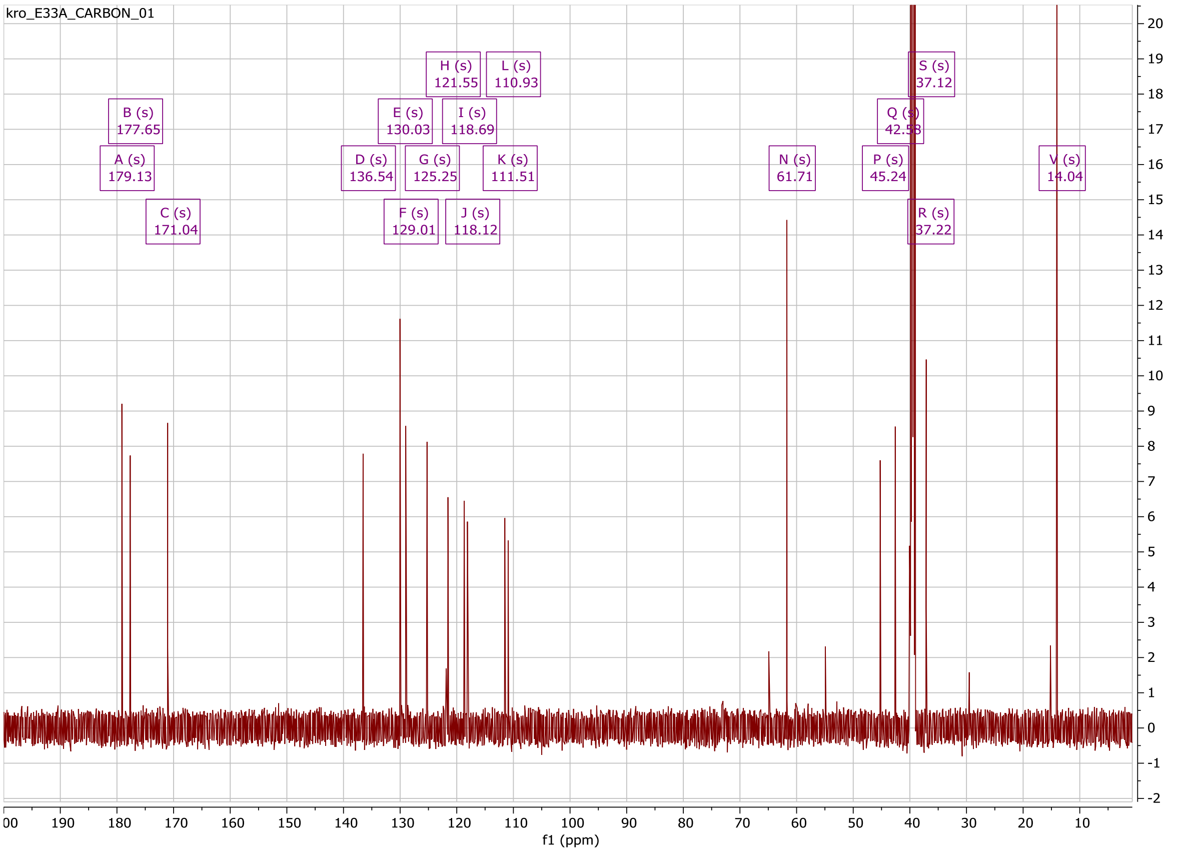

^{1}H NMR spectrum of **22c** in CDCl$_3$

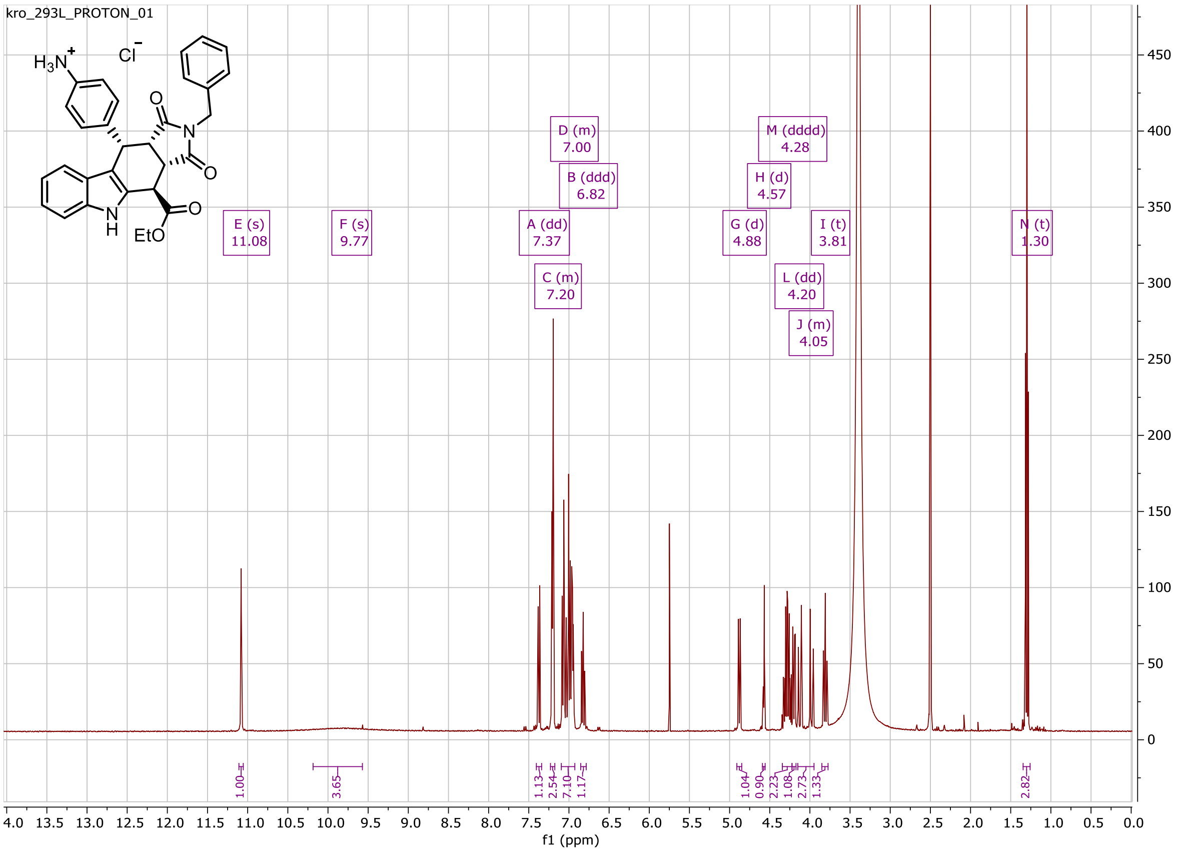

^{13}C NMR spectrum of (±)-**22c**·HCl in DMSO-d_6.

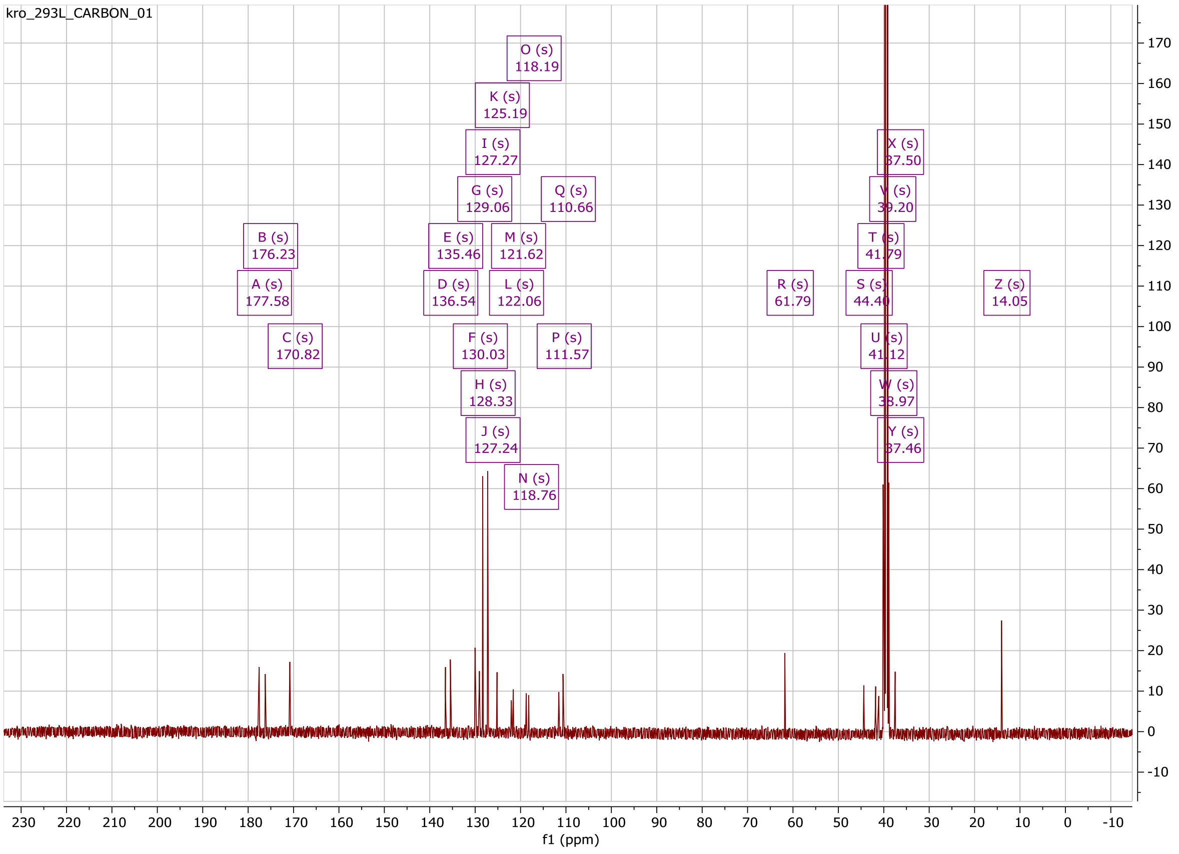

^{1}H NMR spectrum of **23** in $CDCl_3$

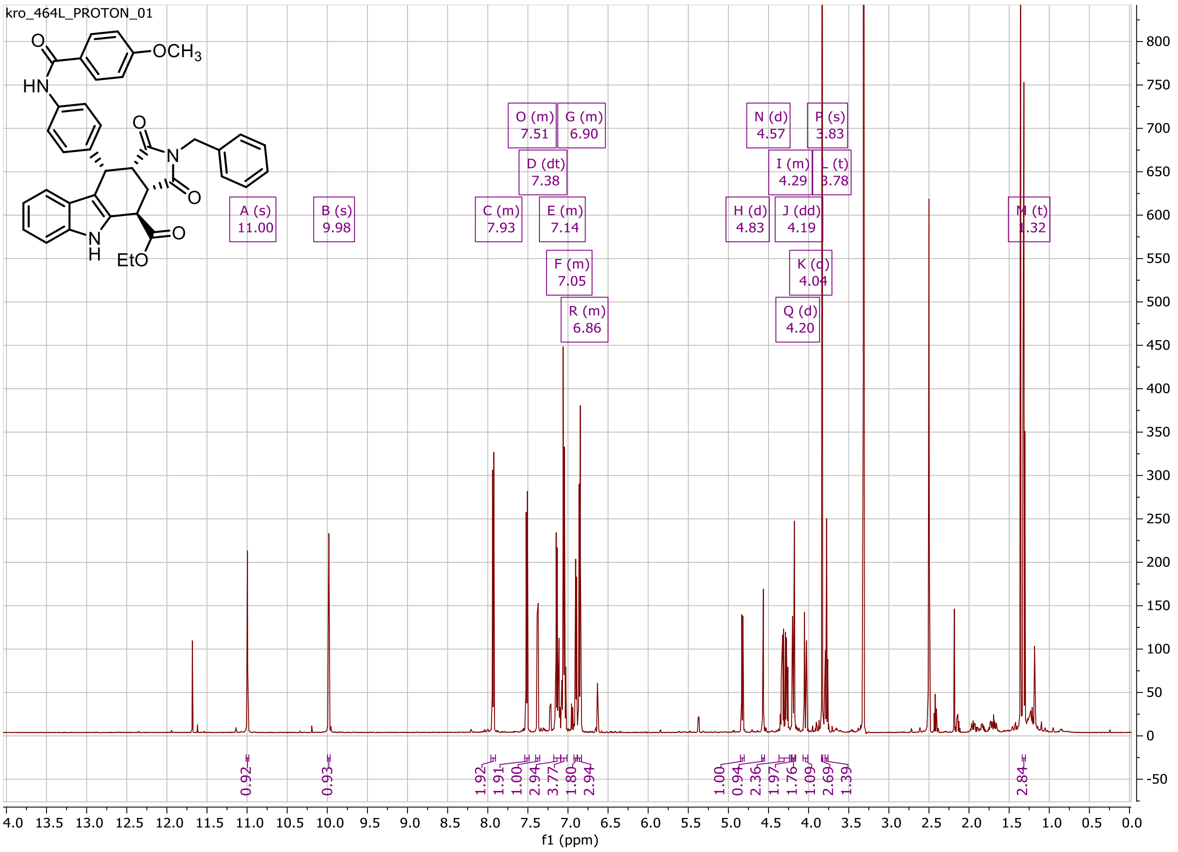

^{13}C NMR spectrum of (±)-**23** in DMSO-d_6.

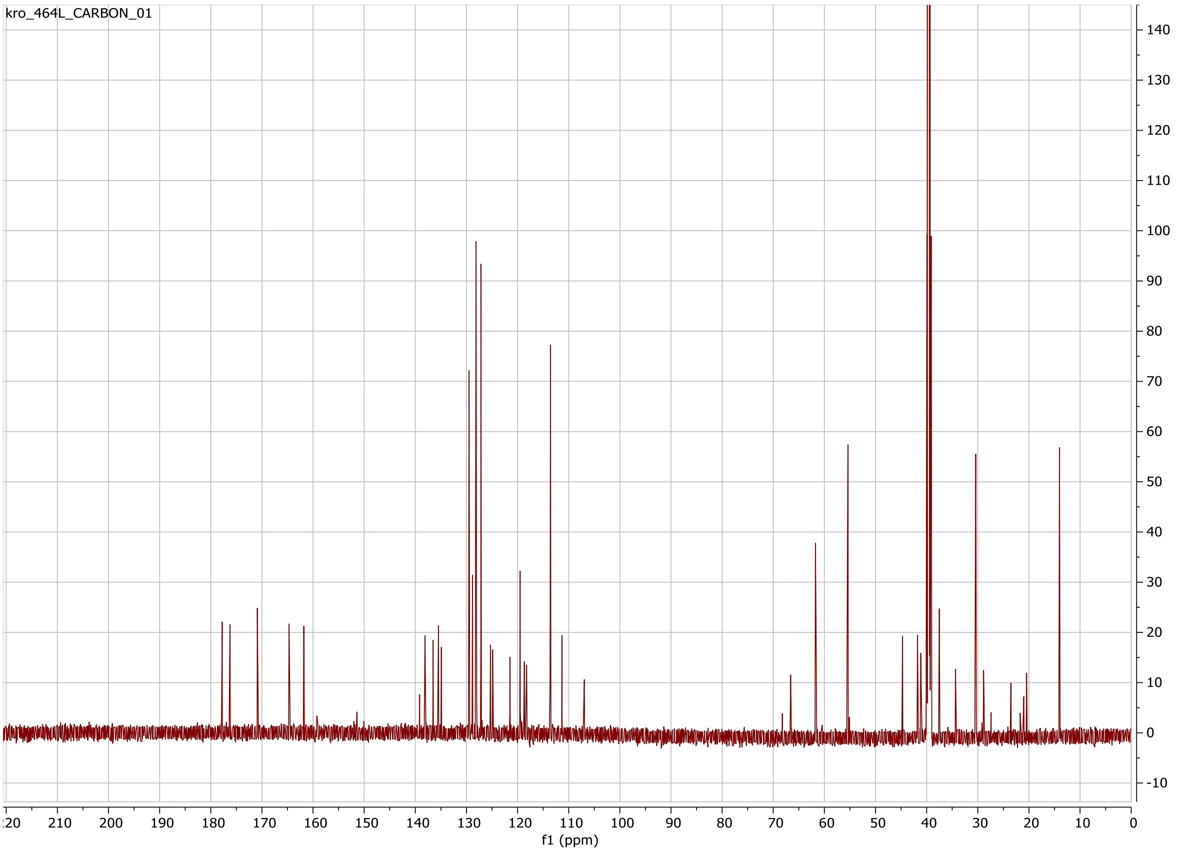

5. Purity by HPLC analysis

HPLC analysis of compound **9a**

Instrument:AKBW_CHROMNI2 Sequence:Chromni_16_12_14 Page 8 of 21

Chromatogram and Results

Injection Details

Injection Name:	**99L**	*Run Time (min):*	**30,00**
Vial Number:	**GA5**	*Injection Volume:*	**5,00**
Injection Type:	**Unknown**	*Channel:*	**UV_VIS_1**
Calibration Level:		*Wavelength:*	**210,0**
Instrument Method:	**Chromni**	*Bandwidth:*	**n.a.**
Processing Method:	**Chromni Processing Method**	*Dilution Factor:*	**1,0000**
Injection Date/Time:	**14.Dez.16 18:17**	*Sample Weight:*	**1,0000**

Chromatogram

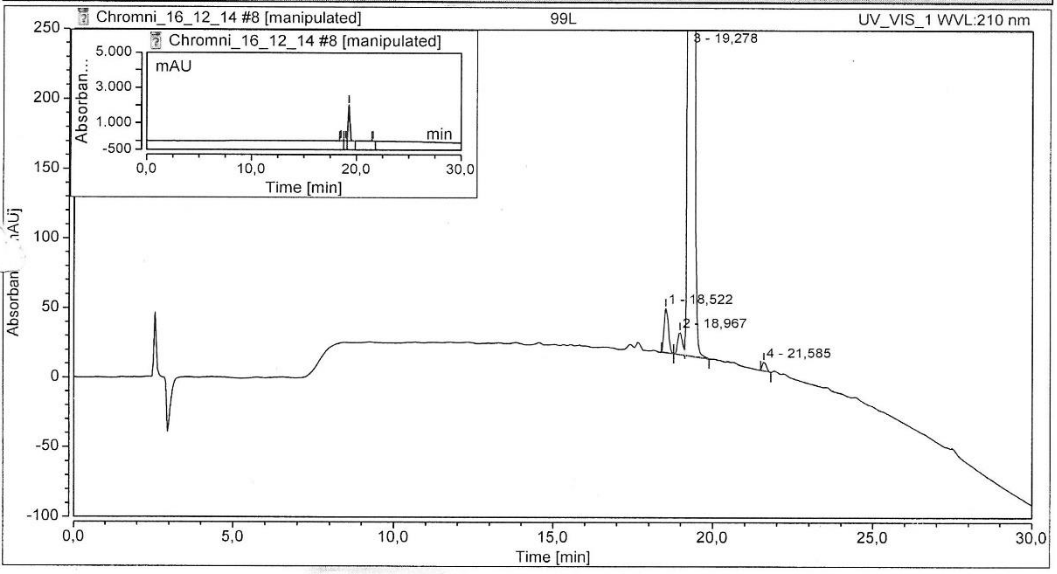

Integration Results

No.	Peak Name	Retention Time min	Area mAU*min	Height mAU	Relative Area %	Relative Height %	Amount n.a.
1		18,522	4,960	31,635	1,52	1,54	n.a.
2		18,967	2,738	15,965	0,84	0,78	n.a.
3		19,278	316,918	2002,559	97,35	97,38	n.a.
4		21,585	0,943	6,326	0,29	0,31	n.a.
**Total:**			**325,559**	**2056,486**	**100,00**	**100,00**	

Default/Integration

Chromeleon (c) Dionex
Version 7.2.0.3765

HPLC analysis of compound **9b**

Chromatogram and Results

Injection Details

Injection Name:	**133B**	*Run Time (min):*	**30,00**
Vial Number:	**GB1**	*Injection Volume:*	**5,00**
Injection Type:	**Unknown**	*Channel:*	**UV_VIS_1**
Calibration Level:		*Wavelength:*	**210,0**
Instrument Method:	**Chromni**	*Bandwidth:*	**n.a.**
Processing Method:	**Chromni Processing Method**	*Dilution Factor:*	**1,0000**
Injection Date/Time:	**05.Apr.17 23:15**	*Sample Weight:*	**1,0000**

Chromatogram

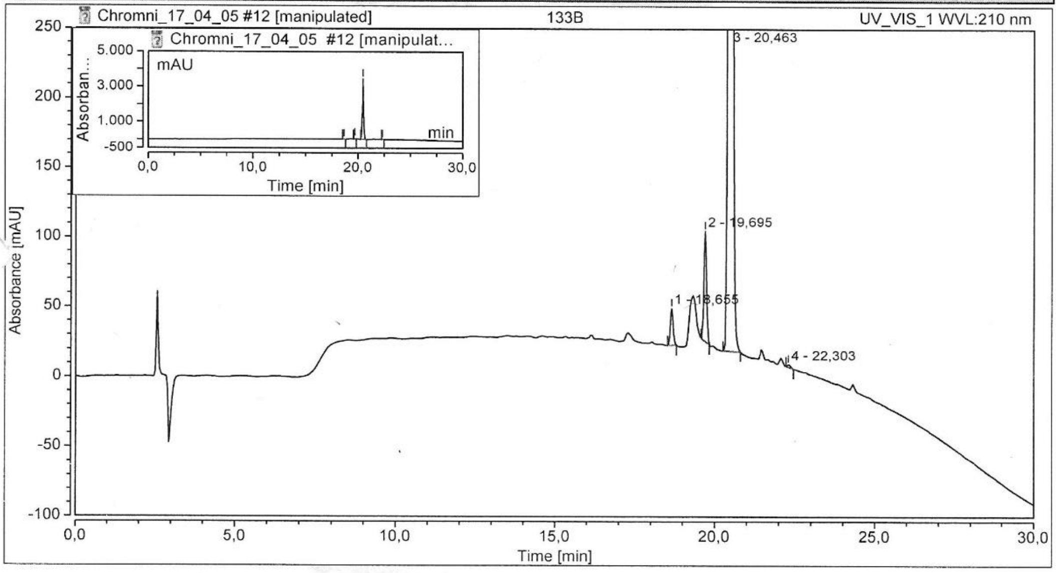

Integration Results

No.	Peak Name	Retention Time min	Area mAU*min	Height mAU	Relative Area %	Relative Height %	Amount n.a.
1		18,655	2,608	26,274	0,67	0,74	n.a.
2		19,695	7,600	78,884	1,95	2,22	n.a.
3		20,463	378,721	3440,891	97,32	96,98	n.a.
4		22,303	0,221	1,962	0,06	0,06	n.a.
**Total:**			**389,150**	**3548,011**	**100,00**	**100,00**	

Chromeleon (c) Dionex
Version 7.2.0.3765

HPLC analysis of compound **10**

Instrument:AKBW_CHROMNI2 Sequence:Chromni_16_12_14 Page 11 of 21

Chromatogram and Results

Injection Details

Injection Name:	**103L**	*Run Time (min):*	**30,00**
Vial Number:	**GA8**	*Injection Volume:*	**5,00**
Injection Type:	**Unknown**	*Channel:*	**UV_VIS_1**
Calibration Level:		*Wavelength:*	**210,0**
Instrument Method:	**Chromni**	*Bandwidth:*	**n.a.**
Processing Method:	**Chromni Processing Method**	*Dilution Factor:*	**1,0000**
Injection Date/Time:	**14.Dez.16 20:19**	*Sample Weight:*	**1,0000**

Chromatogram

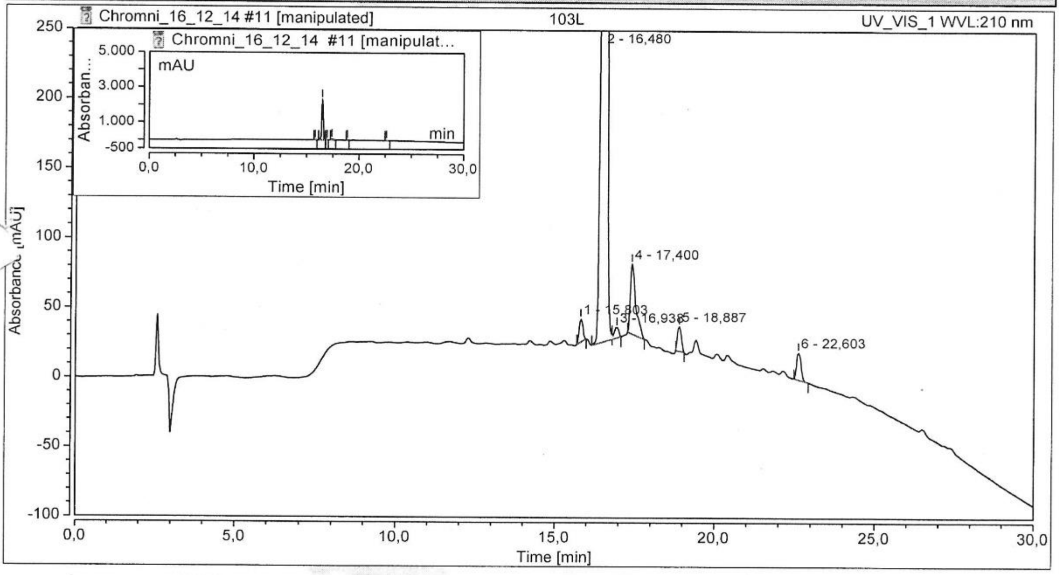

Integration Results

No.	Peak Name	Retention Time min	Area mAU*min	Height mAU	Relative Area %	Relative Height %	Amount n.a.
1		15,803	2,291	16,768	0,62	0,69	n.a.
2		16,480	347,499	2302,659	94,66	95,34	n.a.
3		16,938	1,304	7,764	0,36	0,32	n.a.
4		17,400	10,194	50,415	2,78	2,09	n.a.
5		18,887	2,621	17,738	0,71	0,73	n.a.
6		22,603	3,195	19,886	0,87	0,82	n.a.
**Total:**			**367,104**	**2415,231**	**100,00**	**100,00**	

HPLC analysis of compound **11**

Instrument:AKBW_CHROMNI2 Sequence:Chromni_17_01_12 Page 36 of 39

Chromatogram and Results

Injection Details

Injection Name:	**110A**	*Run Time (min):*	**30,00**
Vial Number:	**GD8**	*Injection Volume:*	**5,00**
Injection Type:	**Unknown**	*Channel:*	**UV_VIS_1**
Calibration Level:		*Wavelength:*	**210,0**
Instrument Method:	**Chromni**	*Bandwidth:*	**n.a.**
Processing Method:	**Chromni Processing Method**	*Dilution Factor:*	**1,0000**
Injection Date/Time:	**13.Jan.17 15:16**	*Sample Weight:*	**1,0000**

Chromatogram

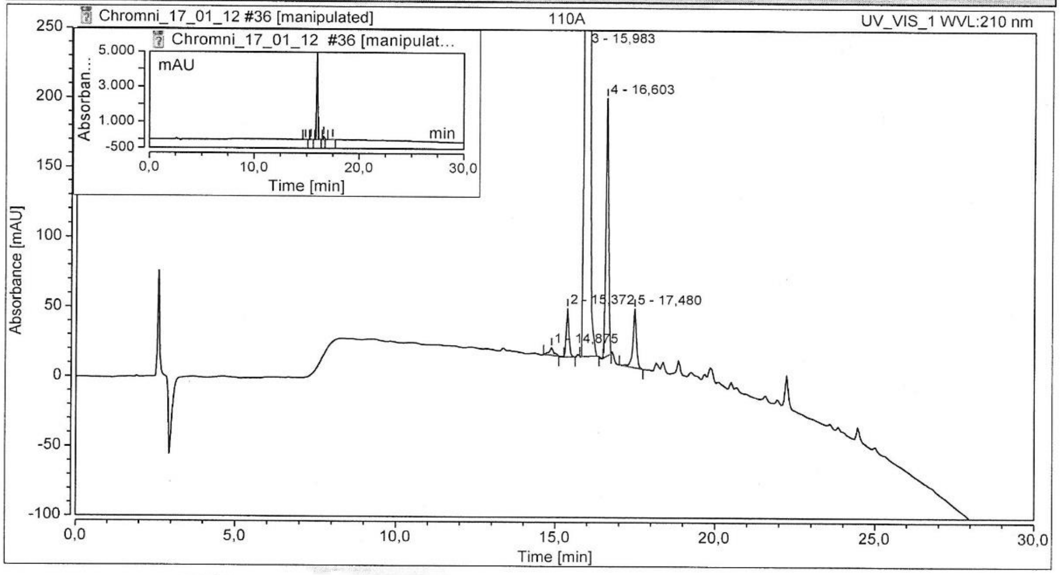

Integration Results

No.	Peak Name	Retention Time min	Area mAU*min	Height mAU	Relative Area %	Relative Height %	Amount n.a.
1		14,875	0,949	5,562	0,16	0,11	n.a.
2		15,372	3,322	34,830	0,54	0,66	n.a.
3		15,983	583,415	4984,107	95,51	94,90	n.a.
4		16,603	17,856	185,056	2,92	3,52	n.a.
5		17,480	5,312	42,369	0,87	0,81	n.a.
**Total:**			**610,854**	**5251,925**	**100,00**	**100,00**	

Default/Integration

Chromeleon (c) Dionex
Version 7.2.0.3765

HPLC analysis of compound **12**

Chromatogram and Results

Injection Details

Injection Name:	**118A**	*Run Time (min):*	**30,00**
Vial Number:	**GC2**	*Injection Volume:*	**5,00**
Injection Type:	**Unknown**	*Channel:*	**UV_VIS_1**
Calibration Level:		*Wavelength:*	**210,0**
Instrument Method:	**Chromni**	*Bandwidth:*	**n.a.**
Processing Method:	**Chromni Processing Method**	*Dilution Factor:*	**1,0000**
Injection Date/Time:	**02.Feb.17 05:53**	*Sample Weight:*	**1,0000**

Chromatogram

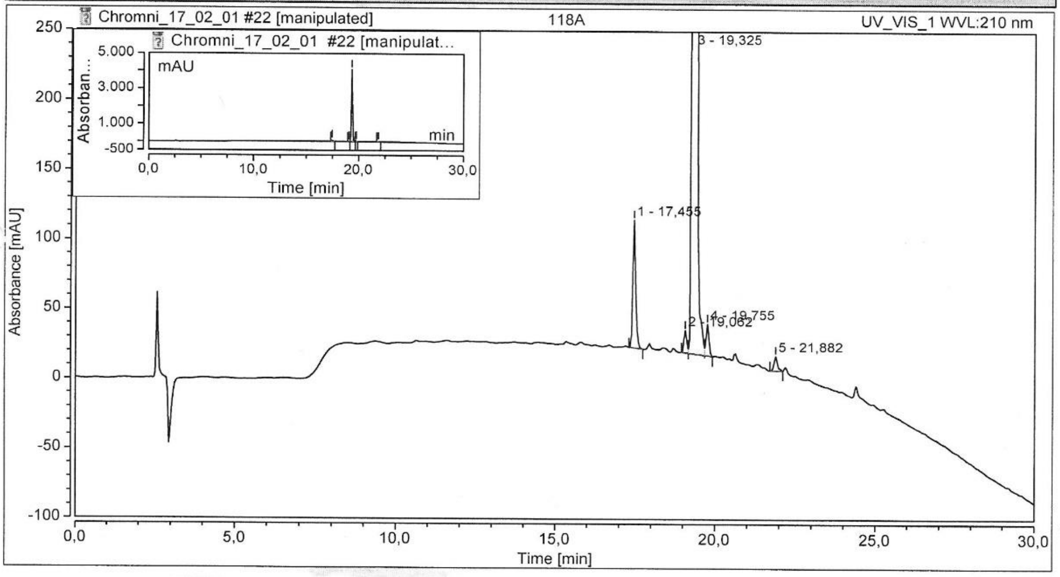

Integration Results

No.	Peak Name	Retention Time min	Area mAU*min	Height mAU	Relative Area %	Relative Height %	Amount n.a.
1		17,455	9,523	91,665	2,02	2,18	n.a.
2		19,062	1,619	15,991	0,34	0,38	n.a.
3		19,325	457,017	4071,355	96,86	96,66	n.a.
4		19,755	2,374	22,846	0,50	0,54	n.a.
5		21,882	1,310	10,344	0,28	0,25	n.a.
**Total:**			**471,843**	**4212,202**	**100,00**	**100,00**	

HPLC analysis of compound **13**

Chromatogram and Results

Injection Details

Injection Name:	**L113A**	*Run Time (min):*	**30,00**
Vial Number:	**GB4**	*Injection Volume:*	**5,00**
Injection Type:	**Unknown**	*Channel:*	**UV_VIS_1**
Calibration Level:		*Wavelength:*	**210,0**
Instrument Method:	**Chromni**	*Bandwidth:*	**n.a.**
Processing Method:	**Chromni Processing Method**	*Dilution Factor:*	**1,0000**
Injection Date/Time:	**19.Jan.17 01:00**	*Sample Weight:*	**1,0000**

Chromatogram

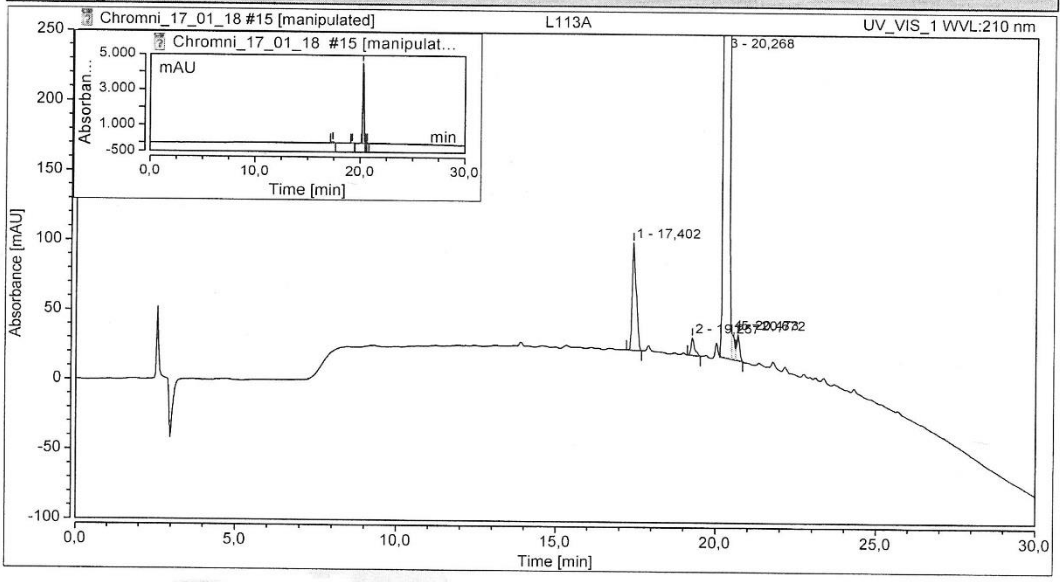

Integration Results

No.	Peak Name	Retention Time min	Area mAU*min	Height mAU	Relative Area %	Relative Height %	Amount n.a.
1		17,402	11,103	77,296	2,06	1,66	n.a.
2		19,257	1,616	12,752	0,30	0,27	n.a.
3		20,268	523,047	4526,830	96,92	97,27	n.a.
4		20,473	1,885	18,089	0,35	0,39	n.a.
5		20,672	2,010	18,738	0,37	0,40	n.a.
**Total:**			**539,661**	**4653,704**	**100,00**	**100,00**	

HPLC analysis of compound **14**

Instrument:AKBW_CHROMNI2 Sequence:Chromni_17_02_01 Page 23 of 42

Chromatogram and Results

Injection Details

Injection Name:	**116B**	*Run Time (min):*	**30,00**
Vial Number:	**GC1**	*Injection Volume:*	**5,00**
Injection Type:	**Unknown**	*Channel:*	**UV_VIS_1**
Calibration Level:		*Wavelength:*	**210,0**
Instrument Method:	**Chromni**	*Bandwidth:*	**n.a.**
Processing Method:	**Chromni Processing Method**	*Dilution Factor:*	**1,0000**
Injection Date/Time:	**02.Feb.17 05:13**	*Sample Weight:*	**1,0000**

Chromatogram

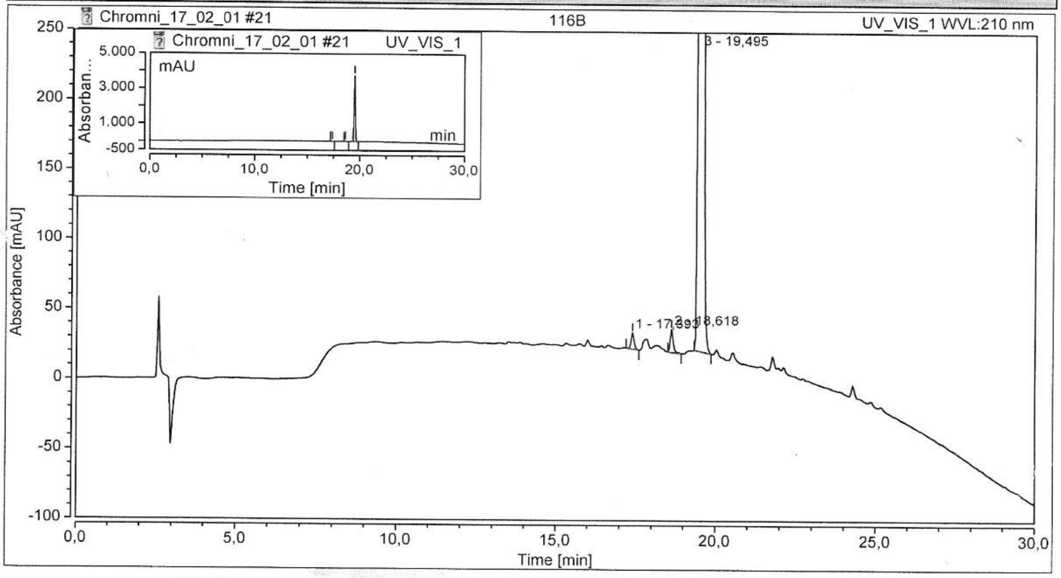

Integration Results

No.	Peak Name	Retention Time min	Area mAU*min	Height mAU	Relative Area %	Relative Height %	Amount n.a.
1		17,393	1,238	11,624	0,26	0,31	n.a.
2		18,618	1,755	16,386	0,36	0,43	n.a.
3		19,495	479,073	3745,499	99,38	99,26	n.a.
**Total:**			**482,066**	**3773,509**	**100,00**	**100,00**	

Default/Integration

Chromeleon (c) Dionex
Version 7.2.0.3765

HPLC analysis of compound **15**

Chromatogram and Results

Injection Details

Injection Name:	**L114B**	*Run Time (min):*	**30,00**
Vial Number:	**GB5**	*Injection Volume:*	**5,00**
Injection Type:	**Unknown**	*Channel:*	**UV_VIS_1**
Calibration Level:		*Wavelength:*	**210,0**
Instrument Method:	**Chromni**	*Bandwidth:*	**n.a.**
Processing Method:	**Chromni Processing Method**	*Dilution Factor:*	**1,0000**
Injection Date/Time:	**19.Jan.17 01:41**	*Sample Weight:*	**1,0000**

Chromatogram

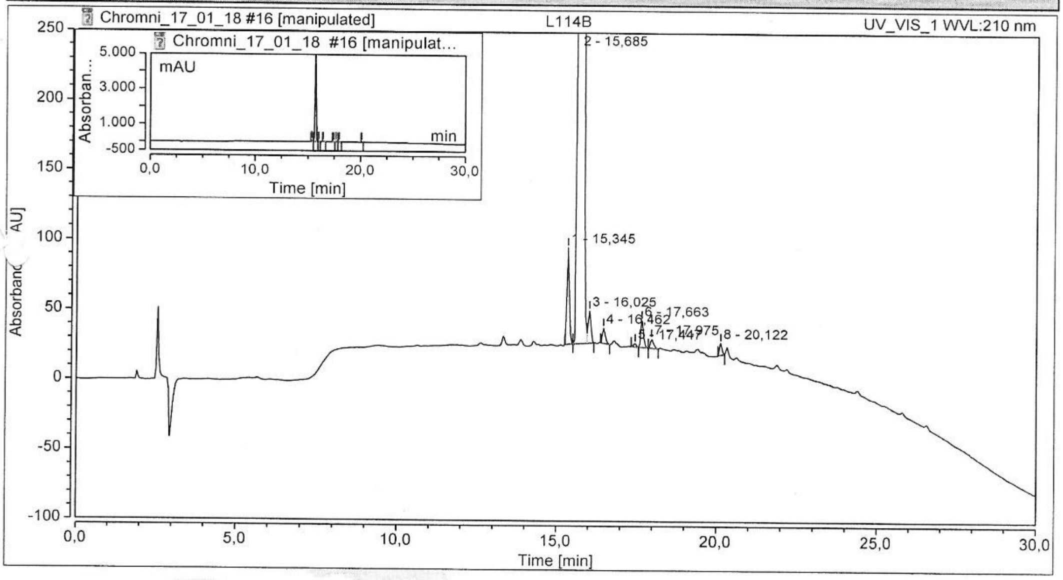

Integration Results

No.	Peak Name	Retention Time min	Area mAU*min	Height mAU	Relative Area %	Relative Height %	Amount n.a.
1		15,345	6,216	68,888	1,11	1,35	n.a.
2		15,685	545,147	4971,835	97,59	97,26	n.a.
3		16,025	2,294	23,210	0,41	0,45	n.a.
4		16,462	1,201	11,022	0,21	0,22	n.a.
5		17,447	0,268	2,463	0,05	0,05	n.a.
6		17,663	1,915	19,225	0,34	0,38	n.a.
7		17,975	0,774	6,634	0,14	0,13	n.a.
8		20,122	0,794	8,559	0,14	0,17	n.a.
**Total:**			**558,608**	**5111,836**	**100,00**	**100,00**	

HPLC analysis of compound **16**

Instrument:AKBW_CHROMNI2 Sequence:Chromni_17_02_08 Page 22 of 23

Chromatogram and Results

Injection Details

Injection Name:	**119A**	*Run Time (min):*	**30,00**
Vial Number:	**GC2**	*Injection Volume:*	**5,00**
Injection Type:	**Unknown**	*Channel:*	**UV_VIS_1**
Calibration Level:		*Wavelength:*	**210,0**
Instrument Method:	**Chromni**	*Bandwidth:*	**n.a.**
Processing Method:	**Chromni Processing Method**	*Dilution Factor:*	**1,0000**
Injection Date/Time:	**09.Feb.17 06:18**	*Sample Weight:*	**1,0000**

Chromatogram

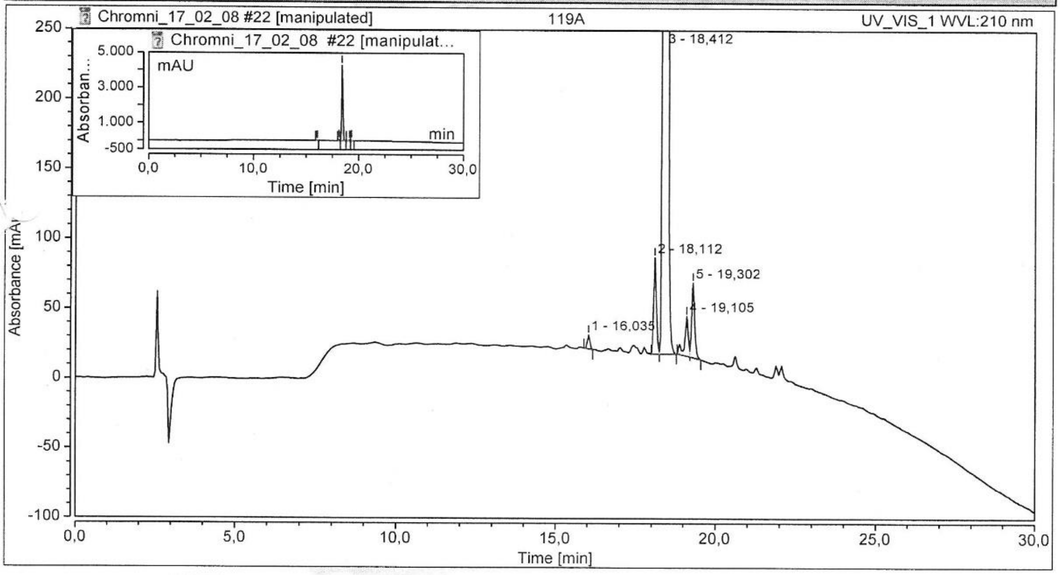

Integration Results

No.	Peak Name	Retention Time min	Area mAU*min	Height mAU	Relative Area %	Relative Height %	Amount n.a.
1		16,035	0,921	10,139	0,18	0,23	n.a.
2		18,112	6,789	69,152	1,33	1,56	n.a.
3		18,412	495,162	4276,604	96,65	96,36	n.a.
4		19,105	3,729	28,626	0,73	0,64	n.a.
5		19,302	5,722	53,801	1,12	1,21	n.a.
**Total:**			**512,323**	**4438,322**	**100,00**	**100,00**	

HPLC analysis of compound **17**

Instrument:AKBW_CHROMNI2 Sequence:Chromni_17_02_15

Page 32 of 33

Chromatogram and Results

Injection Details

Injection Name:	**124A**	*Run Time (min):*	**30,00**
Vial Number:	**GD4**	*Injection Volume:*	**5,00**
Injection Type:	**Unknown**	*Channel:*	**UV_VIS_1**
Calibration Level:		*Wavelength:*	**210,0**
Instrument Method:	**Chromni**	*Bandwidth:*	**n.a.**
Processing Method:	**Chromni Processing Method**	*Dilution Factor:*	**1,0000**
Injection Date/Time:	**16.Feb.17 13:23**	*Sample Weight:*	**1,0000**

Chromatogram

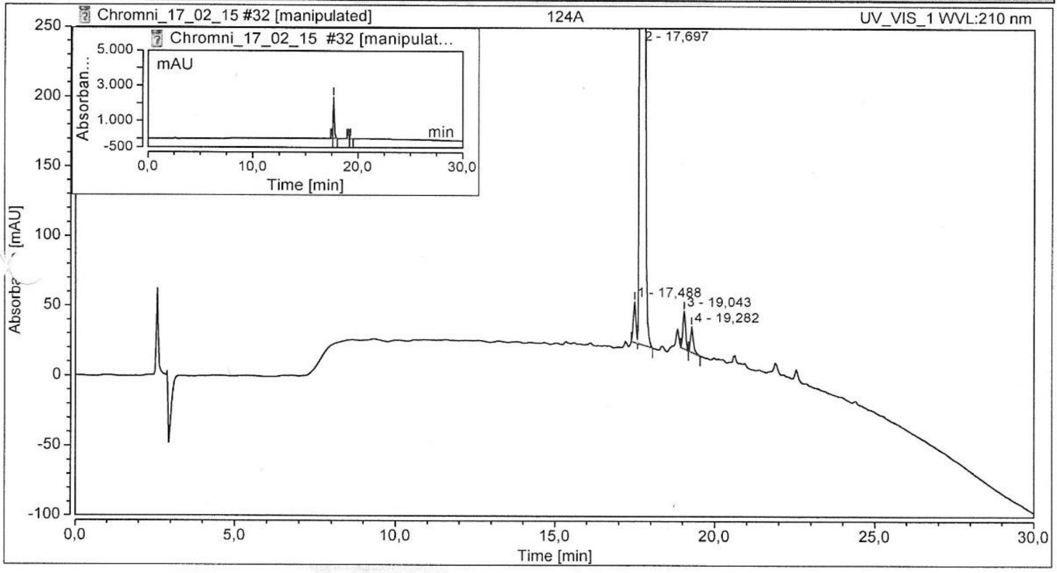

Integration Results

No.	Peak Name	Retention Time min	Area mAU*min	Height mAU	Relative Area %	Relative Height %	Amount n.a.
1		17,488	2,712	29,320	1,01	1,22	n.a.
2		17,697	261,819	2327,180	97,34	96,88	n.a.
[illegible]		19,043	2,528	27,147	0,94	1,13	n.a.
4		19,282	1,902	18,374	0,71	0,76	n.a.
**Total:**			**268,961**	**2402,021**	**100,00**	**100,00**	

HPLC analysis of compound **18a**

Chromatogram and Results

Injection Details

Injection Name:	**126A**	*Run Time (min):*	**30,00**
Vial Number:	**GC8**	*Injection Volume:*	**5,00**
Injection Type:	**Unknown**	*Channel:*	**UV_VIS_1**
Calibration Level:		*Wavelength:*	**210,0**
Instrument Method:	**Chromni**	*Bandwidth:*	**n.a.**
Processing Method:	**Chromni Processing Method**	*Dilution Factor:*	**1,0000**
Injection Date/Time:	**17.Mai.18 10:05**	*Sample Weight:*	**1,0000**

Chromatogram

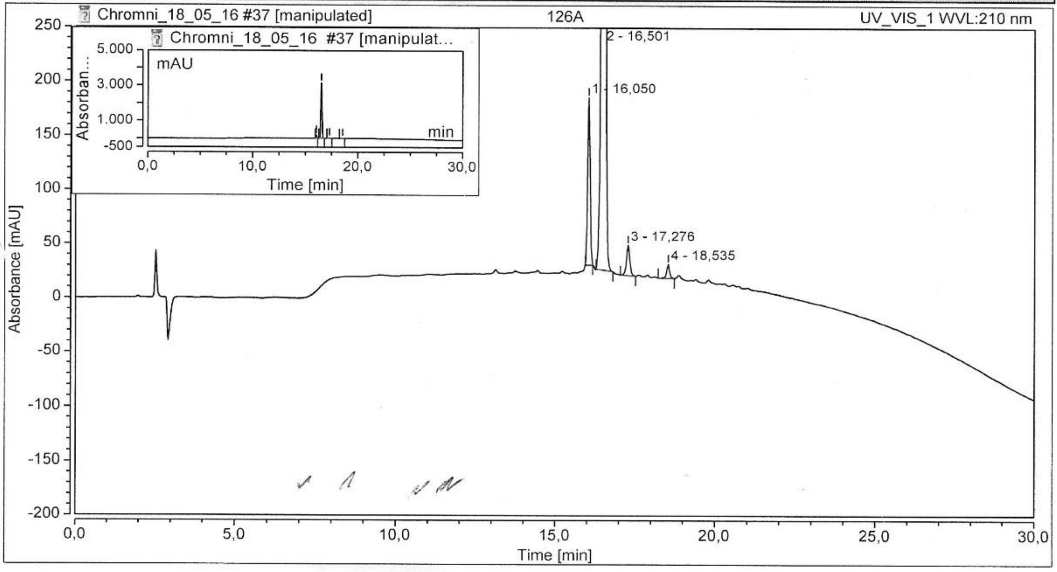

Integration Results

No.	Peak Name	Retention Time min	Area mAU*min	Height mAU	Relative Area %	Relative Height %	Amount n.a.
1		16,050	13,412	154,577	4,62	4,65	n.a.
2		16,501	272,111	3129,695	93,70	94,12	n.a.
3		17,276	3,476	28,072	1,20	0,84	n.a.
4		18,535	1,416	13,018	0,49	0,39	n.a.
**Total:**			**290,415**	**3325,362**	**100,00**	**100,00**	

HPLC analysis of compound **18b**

Instrument:AKBW_CHROMNI2 Sequence:Chromni_18_05_16

Chromatogram and Results

Injection Details

Injection Name:	**126B**	*Run Time (min):*	**30,00**
Vial Number:	**GD1**	*Injection Volume:*	**5,00**
Injection Type:	**Unknown**	*Channel:*	**UV_VIS_1**
Calibration Level:		*Wavelength:*	**210,0**
Instrument Method:	**Chromni**	*Bandwidth:*	**n.a.**
Processing Method:	**Chromni Processing Method**	*Dilution Factor:*	**1,0000**
Injection Date/Time:	**17.Mai.18 10:45**	*Sample Weight:*	**1,0000**

Chromatogram

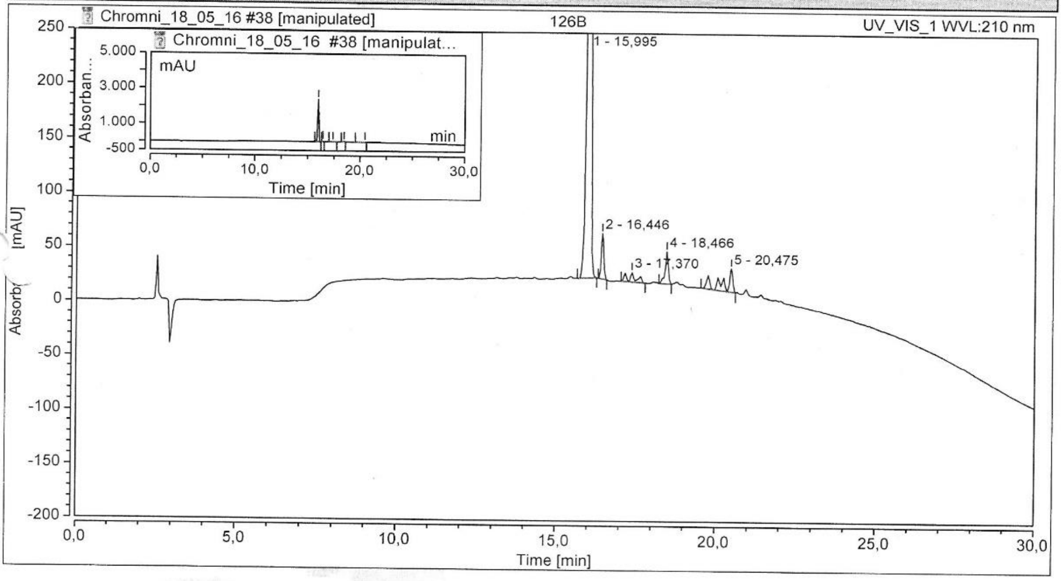

Integration Results

No.	Peak Name	Retention Time min	Area mAU*min	Height mAU	Relative Area %	Relative Height %	Amount n.a.
1		15,995	220,756	2387,363	93,26	95,89	n.a.
2		16,446	3,795	42,058	1,60	1,69	n.a.
3		17,370	2,434	9,087	1,03	0,36	n.a.
4		18,466	3,430	29,585	1,45	1,19	n.a.
5		20,475	6,303	21,683	2,66	0,87	n.a.
**Total:**			**236,719**	**2489,777**	**100,00**	**100,00**	

HPLC analysis of compound **19b**

Chromatogram and Results

Injection Details

Injection Name:	**130A**	*Run Time (min):*	**30,00**
Vial Number:	**GD4**	*Injection Volume:*	**5,00**
Injection Type:	**Unknown**	*Channel:*	**UV_VIS_1**
Calibration Level:		*Wavelength:*	**210,0**
Instrument Method:	**Chromni**	*Bandwidth:*	**n.a.**
Processing Method:	**Chromni Processing Method**	*Dilution Factor:*	**1,0000**
Injection Date/Time:	**02.Mrz.17 13:18**	*Sample Weight:*	**1,0000**

Chromatogram

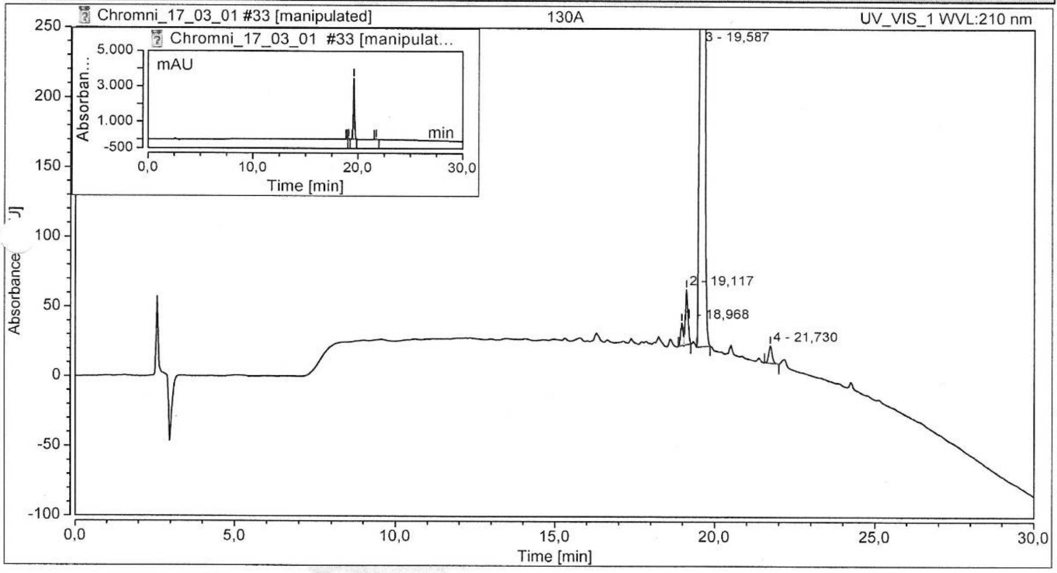

Integration Results

No.	Peak Name	Retention Time min	Area mAU*min	Height mAU	Relative Area %	Relative Height %	Amount n.a.
1		18,968	1,444	16,300	0,41	0,46	n.a.
2		19,117	3,798	39,490	1,07	1,13	n.a.
3		19,587	346,942	3437,632	98,08	98,05	n.a.
4		21,730	1,550	12,573	0,44	0,36	n.a.
**Total:**			**353,734**	**3505,995**	**100,00**	**100,00**	

Chromeleon (c) Dionex
Version 7.2.0.3765

HPLC analysis of compound **20a**

Chromatogram and Results

Injection Details

Injection Name:	**131HCl**	*Run Time (min):*	**30,00**
Vial Number:	**GB5**	*Injection Volume:*	**5,00**
Injection Type:	**Unknown**	*Channel:*	**UV_VIS_1**
Calibration Level:		*Wavelength:*	**210,0**
Instrument Method:	**Chromni**	*Bandwidth:*	**n.a.**
Processing Method:	**Chromni Processing Method**	*Dilution Factor:*	**1,0000**
Injection Date/Time:	**30.Mrz.17 02:38**	*Sample Weight:*	**1,0000**

Chromatogram

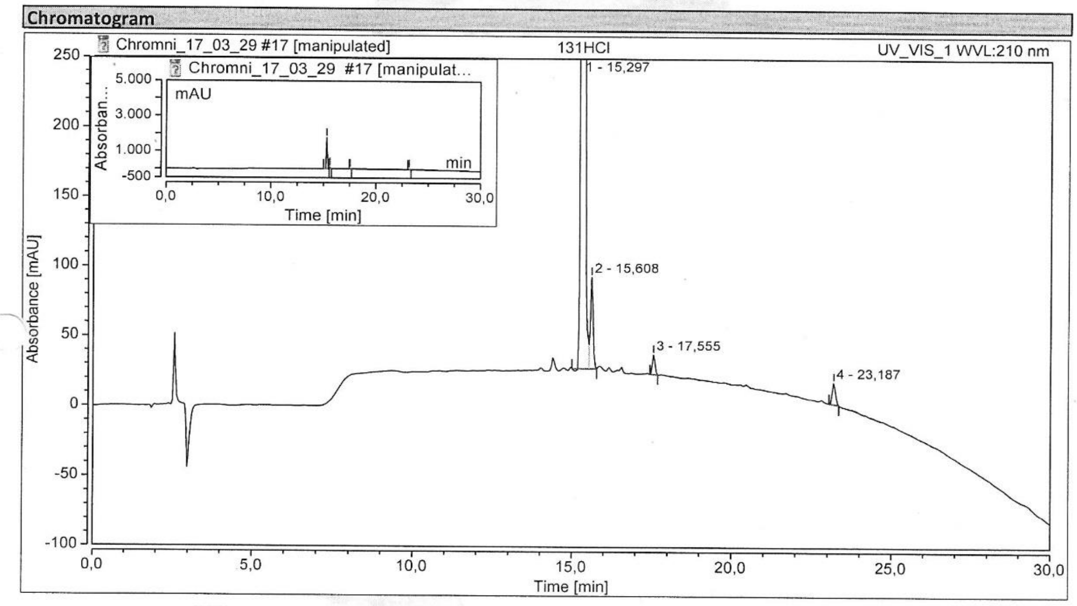

Integration Results

No.	Peak Name	Retention Time min	Area mAU*min	Height mAU	Relative Area %	Relative Height %	Amount n.a.
1		15,297	183,275	1736,283	95,01	94,79	n.a.
2		15,608	6,468	65,659	3,35	3,58	n.a.
3		17,555	1,367	14,252	0,71	0,78	n.a.
4		23,187	1,783	15,441	0,92	0,84	n.a.
**Total:**			**192,892**	**1831,635**	**100,00**	**100,00**	

HPLC analysis of compound **20b**

Instrument:AKBW_CHROMNI2 Sequence:Chromni_17_12_06 Page 13 of 40

Chromatogram and Results

Injection Details

Injection Name:	**165B**	*Run Time (min):*	**30,00**
Vial Number:	**GB2**	*Injection Volume:*	**5,00**
Injection Type:	**Unknown**	*Channel:*	**UV_VIS_1**
Calibration Level:		*Wavelength:*	**210,0**
Instrument Method:	**Chromni**	*Bandwidth:*	**n.a.**
Processing Method:	**Chromni Processing Method**	*Dilution Factor:*	**1,0000**
Injection Date/Time:	**06.Dez.17 23:54**	*Sample Weight:*	**1,0000**

Chromatogram

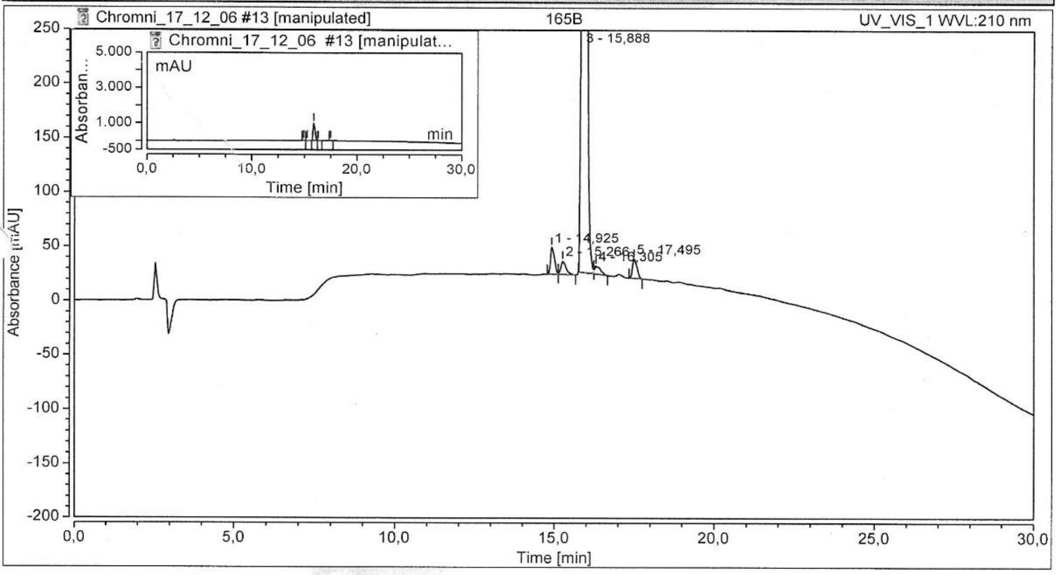

Integration Results

No.	Peak Name	Retention Time min	Area mAU*min	Height mAU	Relative Area %	Relative Height %	Amount n.a.
1		14,925	3,558	24,959	1,98	2,40	n.a.
[illegible]		15,266	2,335	12,563	1,30	1,21	n.a.
3		15,888	169,461	977,670	94,28	93,96	n.a.
4		16,305	1,564	7,478	0,87	0,72	n.a.
5		17,495	2,822	17,868	1,57	1,72	n.a.
**Total:**			**179,740**	**1040,538**	**100,00**	**100,00**	

Default/Integration

Chromeleon (c) Dionex
Version 7.2.0.3765

HPLC analysis of compound **21a**

Instrument:AKBW_CHROMNI2 Sequence:Chromni_16_12_14 Page 8 of 21

Chromatogram and Results

Injection Details

Injection Name:	**99L**	*Run Time (min):*	**30,00**
Vial Number:	**GA5**	*Injection Volume:*	**5,00**
Injection Type:	**Unknown**	*Channel:*	**UV_VIS_1**
Calibration Level:		*Wavelength:*	**210,0**
Instrument Method:	**Chromni**	*Bandwidth:*	**n.a.**
Processing Method:	**Chromni Processing Method**	*Dilution Factor:*	**1,0000**
Injection Date/Time:	**14.Dez.16 18:17**	*Sample Weight:*	**1,0000**

Chromatogram

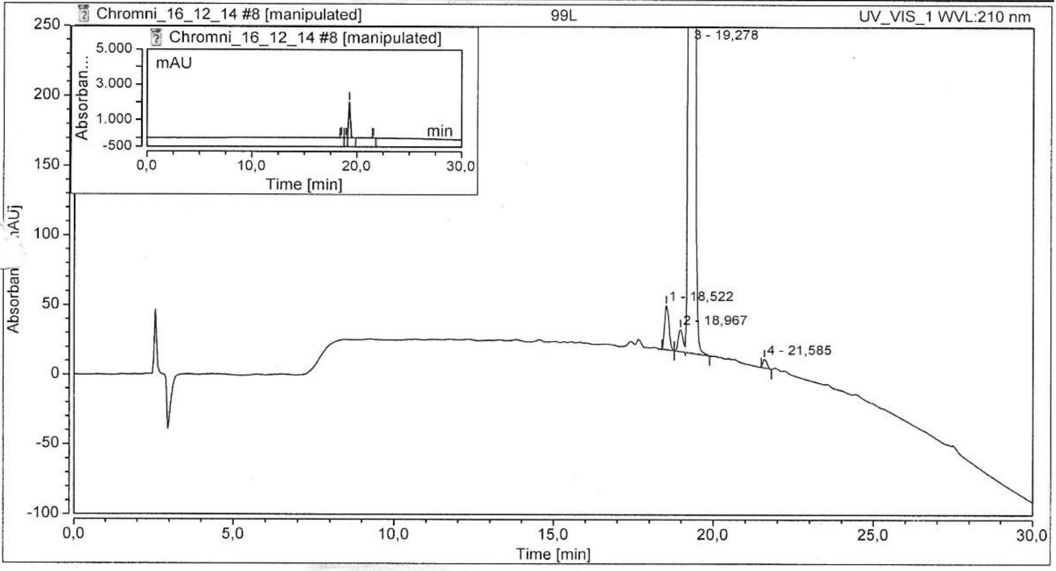

Integration Results

No.	Peak Name	Retention Time min	Area mAU*min	Height mAU	Relative Area %	Relative Height %	Amount n.a.
1		18,522	4,960	31,635	1,52	1,54	n.a.
2		18,967	2,738	15,965	0,84	0,78	n.a.
3		19,278	316,918	2002,559	97,35	97,38	n.a.
4		21,585	0,943	6,326	0,29	0,31	n.a.
**Total:**			**325,559**	**2056,486**	**100,00**	**100,00**	

Default/Integration

Chromeleon (c) Dionex
Version 7.2.0.3765

HPLC analysis of compound **21b**

Chromatogram and Results

Injection Details

Injection Name:	**E55A**	*Run Time (min):*	**30,00**
Vial Number:	**GA8**	*Injection Volume:*	**5,00**
Injection Type:	**Unknown**	*Channel:*	**UV_VIS_1**
Calibration Level:		*Wavelength:*	**210,0**
Instrument Method:	**Chromni**	*Bandwidth:*	**n.a.**
Processing Method:	**Chromni Processing Method**	*Dilution Factor:*	**1,0000**
Injection Date/Time:	**08.Mai.18 23:53**	*Sample Weight:*	**1,0000**

Chromatogram

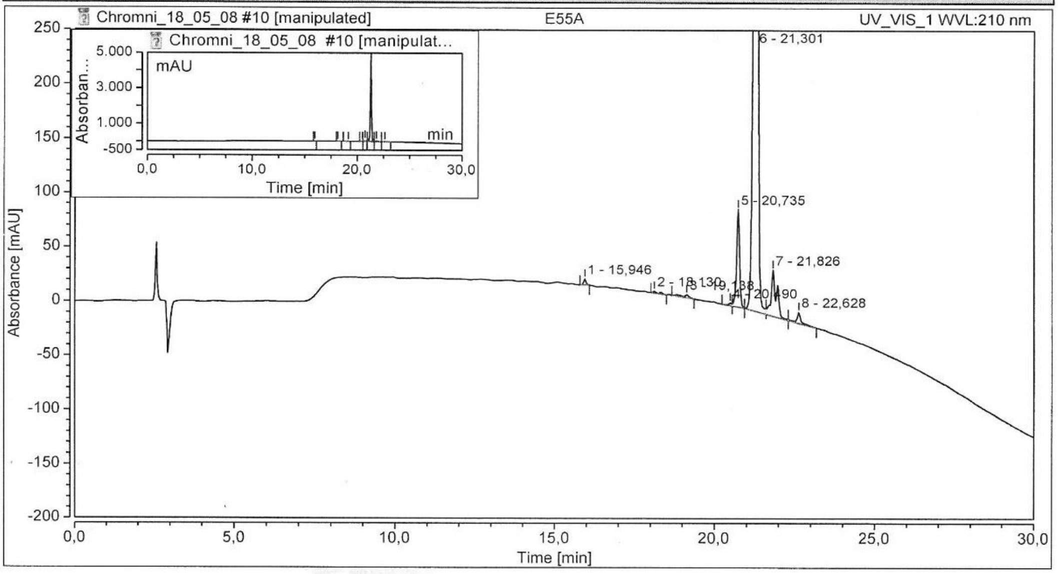

Integration Results

No.	Peak Name	Retention Time min	Area mAU*min	Height mAU	Relative Area %	Relative Height %	Amount n.a.
1		15,946	0,466	5,486	0,10	0,11	n.a.
2		18,130	0,387	1,982	0,09	0,04	n.a.
3		19,138	0,714	2,962	0,16	0,06	n.a.
4		20,490	0,261	2,558	0,06	0,05	n.a.
5		20,735	9,180	89,751	2,05	1,74	n.a.
6		21,301	427,319	5008,614	95,34	96,99	n.a.
7		21,826	8,303	42,355	1,85	0,82	n.a.
8		22,628	1,563	10,088	0,35	0,20	n.a.
**Total:**			**448,193**	**5163,796**	**100,00**	**100,00**	

HPLC analysis of compound **21c**

Instrument:AKBW_CHROMNI2 Sequence:Chromni_18_05_08 Page 19 of 28

Chromatogram and Results

Injection Details

Injection Name:	**192A**	*Run Time (min):*	**30,00**
Vial Number:	**GB8**	*Injection Volume:*	**5,00**
Injection Type:	**Unknown**	*Channel:*	**UV_VIS_1**
Calibration Level:		*Wavelength:*	**210,0**
Instrument Method:	**Chromni**	*Bandwidth:*	**n.a.**
Processing Method:	**Chromni Processing Method**	*Dilution Factor:*	**1,0000**
Injection Date/Time:	**09.Mai.18 05:59**	*Sample Weight:*	**1,0000**

Chromatogram

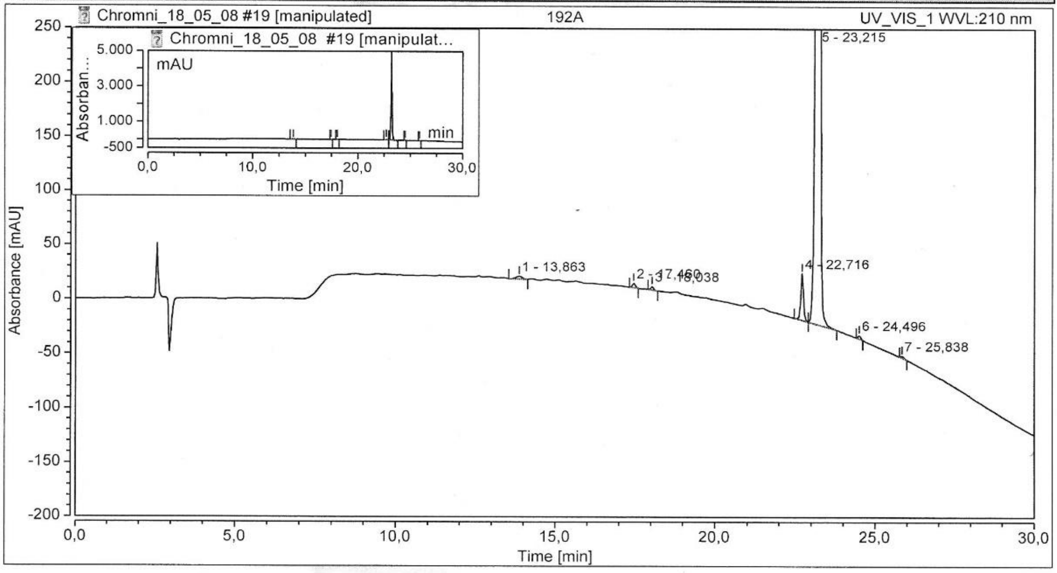

Integration Results

No.	Peak Name	Retention Time min	Area mAU*min	Height mAU	Relative Area %	Relative Height %	Amount n.a.
1		13,863	0,581	2,627	0,13	0,05	n.a.
2		17,460	0,433	4,314	0,10	0,08	n.a.
3		18,038	0,303	3,323	0,07	0,07	n.a.
4		22,716	4,173	42,813	0,94	0,84	n.a.
5		23,215	439,345	5022,576	98,66	98,85	n.a.
6		24,496	0,307	3,445	0,07	0,07	n.a.
7		25,838	0,186	1,832	0,04	0,04	n.a.
**Total:**			**445,329**	**5080,929**	**100,00**	**100,00**	

Chromeleon (c) Dionex
Version 7.2.0.3765

HPLC analysis of compound **22a**

Instrument:AKBW_CHROMNI2 Sequence:Chromni_18_02_07 Page 25 of 41

Chromatogram and Results

Injection Details

Injection Name:	E33A	*Run Time (min):*	30,00
Vial Number:	GC7	*Injection Volume:*	5,00
Injection Type:	Unknown	*Channel:*	UV_VIS_1
Calibration Level:		*Wavelength:*	210,0
Instrument Method:	Chromni	*Bandwidth:*	n.a.
Processing Method:	Chromni Processing Method	*Dilution Factor:*	1,0000
Injection Date/Time:	08.Feb.18 08:08	*Sample Weight:*	1,0000

Chromatogram

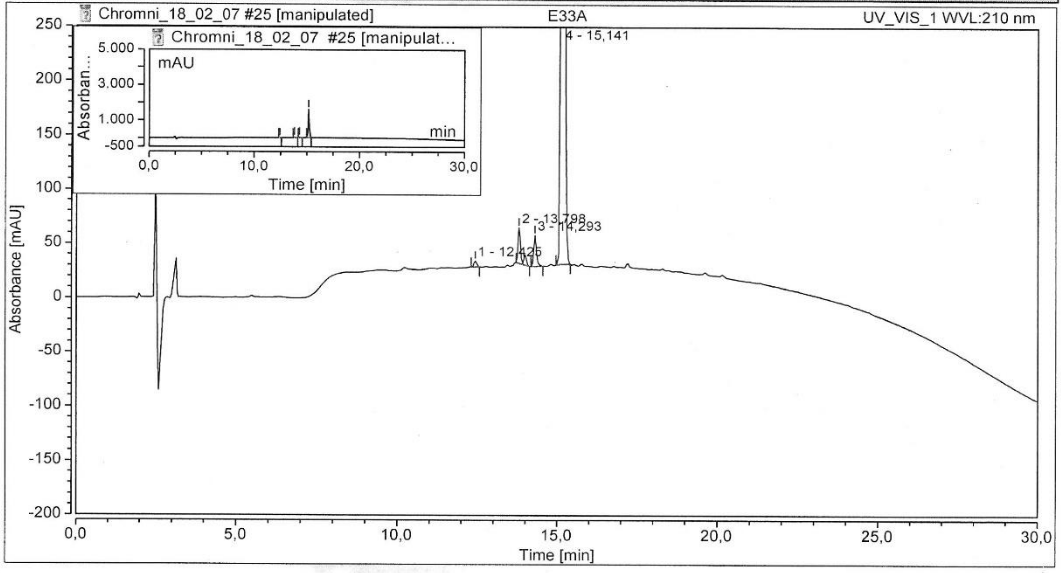

Integration Results

No.	Peak Name	Retention Time min	Area mAU*min	Height mAU	Relative Area %	Relative Height %	Amount n.a.
1		12,425	0,619	5,821	0,36	0,35	n.a.
2		13,798	3,705	32,837	2,14	1,99	n.a.
3		14,293	2,777	28,348	1,60	1,72	n.a.
4		15,141	165,974	1581,828	95,90	95,94	n.a.
**Total:**			**173,074**	**1648,834**	**100,00**	**100,00**	

HPLC analysis of compound **22c**

HPLC

Analyzed: 02.12.15 21:23

Reported: 03.12.15 14:21
Processed: 03.12.15 14:21

Data Path: D:\WIN32APP\HSM\Chromni\DATA\7193\
Application: Chromni
Sample Name: ~~29L2~~ 37L2
Injection from this vial: 1 of 1

Series:7193
Vial Number: 14
Vial Type: UNK
Volume: 5,0 ul

Chrom Type: HPLC Channel : 1

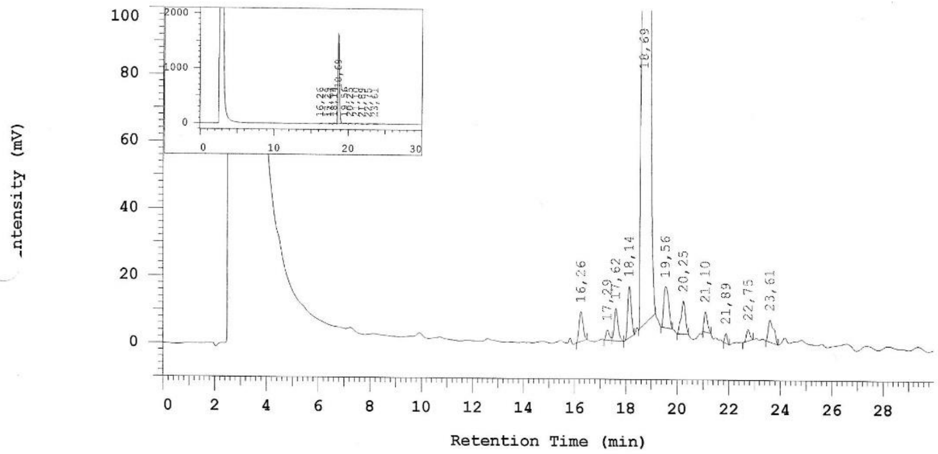

Acquisition Method: Chromni
Blank Subtr Sample Name: ACN
Column Type: 010
Solvent A: Wasser + 0,05%TFA
Developed by: Jens
Solvent B: ACN + 0,05%TFA

No.	RT	Area	Conc 1	BC
1	16,26	93974	0,459	MC
2	17,29	28251	0,138	BB
3	17,62	96680	0,472	BB
4	18,14	141571	0,691	MC
5	18,69	19647315	95,920	MC
6	19,56	160059	0,781	MC
7	20,25	114789	0,560	MC
8	21,10	53539	0,261	MC
9	21,89	19302	0,094	MC
10	22,75	31169	0,152	BB
11	23,61	96359	0,470	BB
		20483008	100,000	

Peak rejection level: 0

HPLC analysis of compound **23**

Instrument:AKBW_CHROMNI2 Sequence:Chromni_18_05_08 Page 21 of 28

Chromatogram and Results

Injection Details

Injection Name:	**046L**	*Run Time (min):*	**30,00**
Vial Number:	**GC1**	*Injection Volume:*	**5,00**
Injection Type:	**Unknown**	*Channel:*	**UV_VIS_1**
Calibration Level:		*Wavelength:*	**210,0**
Instrument Method:	**Chromni**	*Bandwidth:*	**n.a.**
Processing Method:	**Chromni Processing Method**	*Dilution Factor:*	**1,0000**
Injection Date/Time:	**09.Mai.18 07:21**	*Sample Weight:*	**1,0000**

Chromatogram

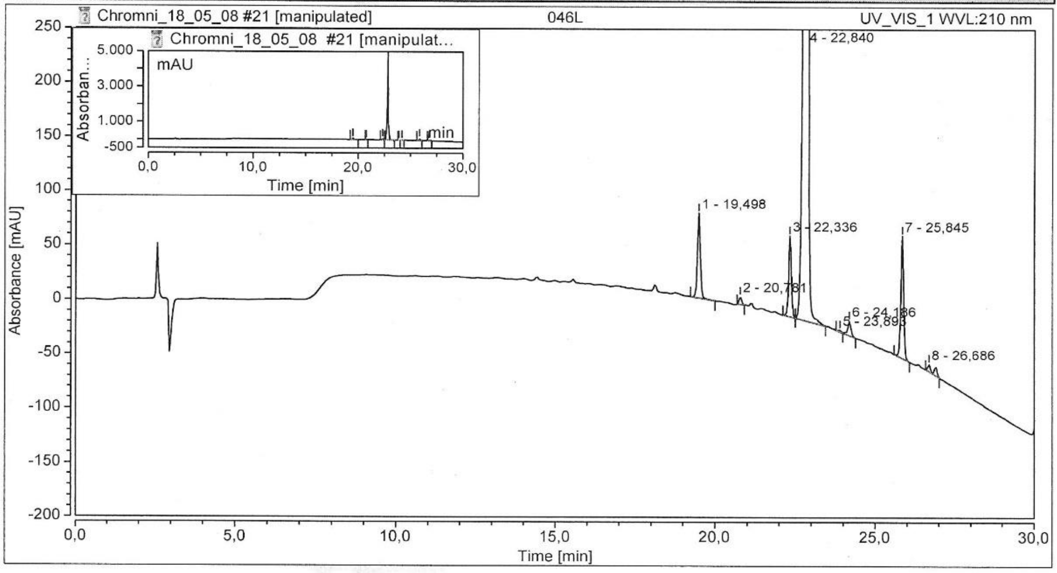

Integration Results

No.	Peak Name	Retention Time<br>min	Area<br>mAU*min	Height<br>mAU	Relative Area<br>%	Relative Height<br>%	Amount<br>n.a.
1		19,498	8,136	77,573	1,47	1,46	n.a.
2		20,781	0,663	7,119	0,12	0,13	n.a.
3		22,336	7,240	73,880	1,31	1,39	n.a.
4		22,840	521,677	5018,644	94,46	94,50	n.a.
5		23,893	0,133	1,383	0,02	0,03	n.a.
6		24,186	1,675	13,415	0,30	0,25	n.a.
7		25,845	11,242	112,201	2,04	2,11	n.a.
8		26,686	1,508	6,733	0,27	0,13	n.a.
**Total:**			**552,273**	**5310,949**	**100,00**	**100,00**	

Chromeleon (c) Dionex
Version 7.2.0.3765
